# Integrative multi-omics identifies *DOC2A* as a novel pharmacological target for bipolar disorder

**DOI:** 10.1017/S0033291726104565

**Published:** 2026-05-28

**Authors:** Chengsong Yuan, Bo Zhang, Yuanyuan Liang, Junze Ran, Shiyi Hu, Andi Liu, Yuran Liu, Fengqin Qin, Lingqi Jian, Yongji He, Feng Han, Chengcheng Zhang

**Affiliations:** 1 Mental Health Center and Psychiatric Laboratory, the State Key Laboratory of Biotherapy, Sichuan University West China Hospital Mental Health Center, China; 2 Department of Nephrology, Sixth People’s Hospital of Chengdu, China; 3 Department of Neurology, The 3rd Affiliated Hospital of Chengdu Medical College, China; 4 Clinical Trial Center, National Medical Products Administration Key Laboratory for Clinical Research and Evaluation of Innovative Drugs, West China Hospital of Sichuan University, China; 5 Department of Emergency Medicine, Hainan General Hospital, Hainan Affiliated Hospital of Hainan Medical University, China

**Keywords:** bipolar disorder, differential expression analysis, *DOC2A*, drug target, Mendelian randomization, PWAS

## Abstract

**Background:**

Current bipolar disorder (BD) therapies suffer from limited efficacy and adverse effects, necessitating mechanistically grounded targets.

**Methods:**

We integrated BD genome-wide association study data (158,036 cases; 2,796,499 controls) with brain proteomics (ROSMAP and Banner dorsolateral prefrontal cortex, *n* = 376 and 152) to perform proteome-wide association studies (PWAS). Bayesian colocalization and summary-data-based Mendelian randomization (SMR) prioritized causal genes. Cell-type-specific transcriptomics validated dysregulation in iPSC-derived neurons, astrocytes, and postmortem hippocampus/prefrontal cortex. Weighted gene co-expression networks (WGCNAs), functional enrichment, and molecular docking assessed functional pathways and druggability.

**Results:**

PWAS identified eight BD-associated genes (false discovery rate < 0.05), with *DOC2A* emerging as the top candidate. Colocalization (*H_4_* > 0.8) and SMR supported a causal association of *DOC2A* with BD, with no pleiotropy (heterogeneity in dependent instruments *P* > 0.01); *DOC2A* expression decreased in BD across neurons (*P* = 4.26 × 10^−2^), astrocytes (*P* = 2.09 × 10^−2^), hippocampus (*P* = 9.80 × 10^−3^, *t* = −2.738), and prefrontal cortex (*P* = 1.44 × 10^−2^, *t* = −2.580); WGCNA positioned *DOC2A* as a key regulator (module membership/gene significance *P* < 0.05) of co-expression networks enriched for BD-associated processes including neurotransmitter secretion and postsynaptic actin cytoskeleton organization (*P* < 0.05); molecular docking revealed favorable-affinity binding (ΔG < −4 kcal/mol) between DOC2A and BD-related drugs and neuroprotective compounds.

**Conclusions:**

Our convergent multi-omics framework highlights *DOC2A* dysregulation as a key contributor to synaptic dysfunction in BD and nominates it as a promising therapeutic target. The demonstrated interaction with existing neuroactive compounds provides immediate translational avenues.

## Introduction

Bipolar disorder (BD), a chronic psychiatric condition affecting over 60 million people globally, is characterized by debilitating oscillations between mania and depression (Anderson, Haddad, & Scott, 2012; Grande, Berk, Birmaher, & Vieta, 2016), with >70% of disease burden attributable to depressive episodes (Miller, Dell’Osso, & Ketter, 2014; Nierenberg et al., 2023) and a staggering 20- to 30-fold increased suicide risk (Miller & Black, 2020; Plans et al., 2019). Despite decades of pharmacological development, first-line therapies (e.g. lithium, antipsychotics) face critical limitations: up to 33% of patients exhibit treatment resistance, relapse rates approach 50% within 2 years, and metabolic side effects from second-generation antipsychotics exacerbate comorbidities (Elsayed, Ercis, Pahwa, & Singh, 2022; Hirsch et al., 2017; Perlis et al., 2006). This therapeutic crisis stems from a fundamental disconnect – while genome-wide association studies (GWASs) have identified >30 risk loci for BD (Cichon et al., 2011; Ferreira et al., 2008; Hou et al., 2016; Mühleisen et al., 2014; Stahl et al., 2019), translating these genetic signals into actionable targets has been hindered by incomplete proteomic validation and poor mechanistic mapping to neural circuits.

Recent multi-omics convergence provides a breakthrough path. Proteome-wide association studies (PWAS), by integrating brain-specific protein quantitative trait loci (pQTLs) with GWAS summary statistics, directly implicate disease-associated protein abundance shifts, overcoming the interpretative limitations of genomic associations alone (Brandes, Linial, & Linial, 2020). For instance, 2025 multi-omics analyses of energy metabolism pathways revealed mitochondrial complex I genes (e.g. *NDUFS2*) as cross-disorder risk factors in BD and schizophrenia (Zou et al., 2025). Similarly, graph convolutional networks fusing brain imaging and gut microbiome data demonstrated 84% accuracy in classifying psychiatric disorders (H. Wang et al., 2025), underscoring the power of multimodal integration. These advances highlight a paradigm shift: bridging molecular cascades from gene→protein→circuit is essential for target discovery in BD’s heterogeneous landscape.

Critically, synaptic dysfunction and bioenergetic deficits constitute converging pathological axes in BD (Cikankova et al., 2017; Schloesser, Huang, Klein, & Manji, 2008). Postmortem studies consistently show synaptic dysfunction in the dorsolateral prefrontal cortex (DLPFC) and hippocampus – key regions regulating emotional processing (Das et al., 2022; Vawter et al., 2002). Mitochondrial impairments disrupt ATP-dependent neurotransmission (Ly & Verstreken, 2006; Machado et al., 2016), while dysregulated Ca^2^⁺ signaling (e.g. via calcium sensor proteins) perturbs vesicle exocytosis and plasticity (Bello et al., 2018; Naoki, Sakumura, & Ishii, 2005). The *DOC2* family, particularly *DOC2A*, regulates spontaneous or asynchronous neurotransmitter release, integrating into presynaptic machinery to fine-tune release dynamics (Pang et al., 2011; Yao, Gaffaney, Kwon, & Chapman, 2011). However, prior genetic studies overlooked *DOC2A*’s protein-level dysregulation and its system-level impacts on BD-relevant pathways.

**Figure 1.**
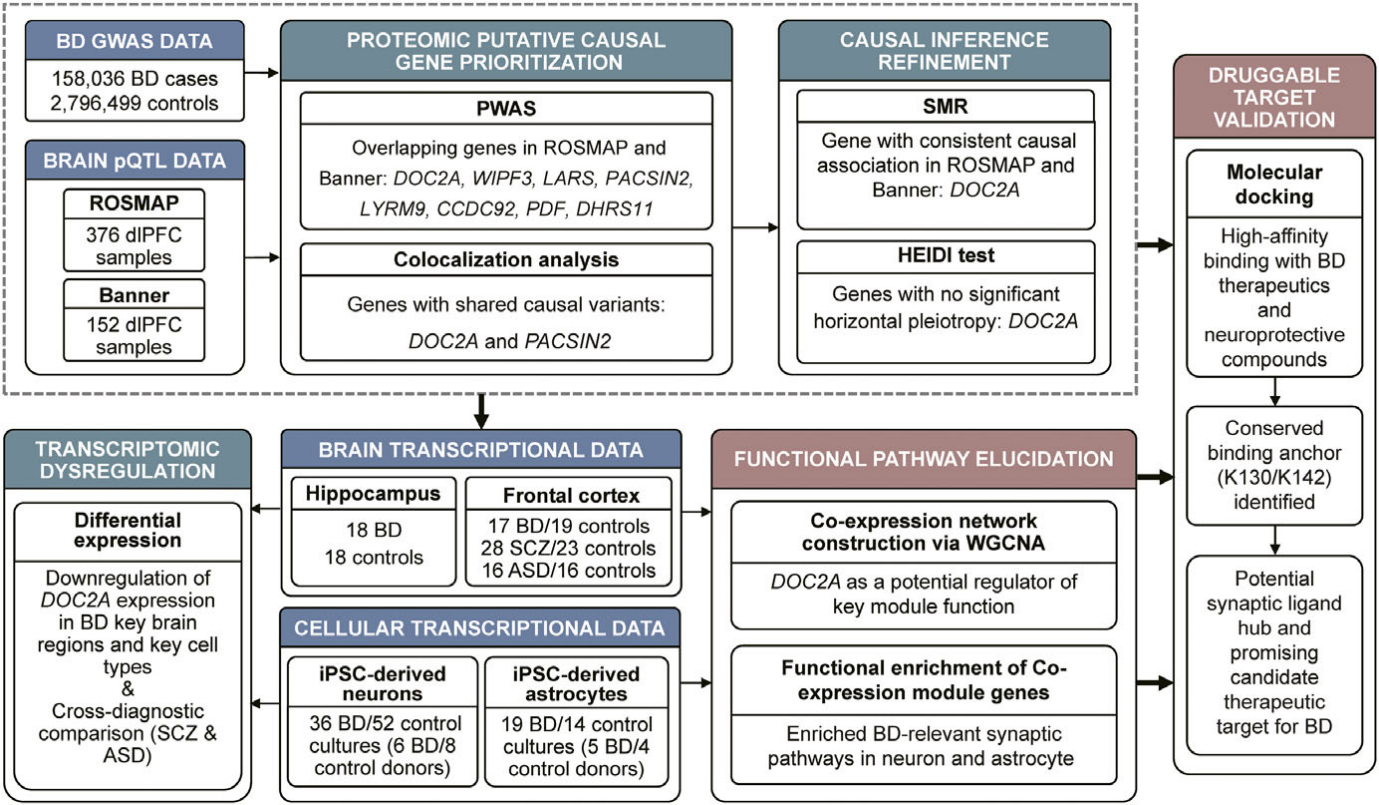
Integrated multi-omics workflow for identifying druggable synaptic targets in BD. Our study implemented a sequential causal inference framework grounded in brain proteomics. First, PWAS were performed by integrating GWAS summary statistics with brain-specific pQTL data from two independent cohorts (ROSMAP and Banner). Second, Bayesian colocalization and SMR were applied to prioritize putatively causal genes. Third, transcriptional dysregulation of the identified genes was evaluated in induced pluripotent stem cell (iPSC)-derived neurons and astrocytes, as well as in prefrontal cortex (PFC) and hippocampus tissues. Fourth, WGCNA was used to identify *DOC2A*-associated modules, followed by functional enrichment analyses (GO, KEGG) to elucidate biological relevance. Finally, molecular docking was performed to assess the binding affinity of DOC2A with FDA-approved and neuroprotective compounds relevant to BD. Figure 1. long description.

Here, we deploy a brain-anchored multi-omics framework to explore the molecular mechanisms underlying BD (Figure 1). We first perform PWAS leveraging pQTLs from DLPFC to prioritize protein-coding risk genes. Bayesian colocalization and summary-data-based Mendelian randomization (SMR) refine causality, followed by differential expression analysis in cell-type-specific contexts (neurons/astrocytes) and disease-vulnerable brain regions. Weighted gene co-expression network analysis (WGCNA) deciphers *DOC2A*-associated co-expression modules, while molecular docking evaluates its potential druggability. Our study nominates *DOC2A* as a synaptic-associated candidate gene in BD pathogenesis, offering a potential novel target for mechanism-based therapeutics.

### Materials and methods

This article integrates multi-omics data from several public resources, as summarized in Supplementary Table S1.

### GWAS data

Summary statistics from the largest BD GWAS (meta-analysis of 79 cohorts; 158,036 cases and 2,796,499 controls as effective samples) were analyzed, spanning European, East Asian, African American, and Latino populations (O’Connell et al., 2025). Downstream analyses in this study used these quality-controlled GWAS summary statistics without additional sample-level filtering performed. Quality control protocols and data preprocessing workflows are described in the original publication (O’Connell et al., 2025).

### Human brain pQTL data

Human brain proteomes from DLPFC were integrated from two cohorts: ROSMAP (*n* = 376) and Banner (*n* = 152) (Beach et al., 2015; Bennett et al., 2018). Cis-regulated pQTL data for 1,475 (ROSMAP) and 1,139 (Banner) proteins with significant pQTL associations were obtained from Wingo et al., and used as reference weights for PWAS. Complete processing protocols are available in the source study (Wingo et al., 2021).

### Proteome-wide association studies

Using the FUSION computational framework, we carried out PWAS to elucidate the role of genetically regulated protein abundance in BD pathogenesis (Gusev et al., 2016). Analyses adopted multiple predictive models (top1, blup, lasso, enet, and bslmm), under which the core PWAS statistic was computed by FUSION as the linear sum of the product of *z*‐score×weight for the independent SNPs at the locus. False discovery rate (FDR) correction was performed via the Benjamini–Hochberg (BH) procedure, with statistical significance defined at *P*_FDR_ < 0.05, ensuring robust associations between proteins and BD.

### Bayesian colocalization for shared causal variants

To evaluate whether protein-disease associations arise from shared causal mechanisms, we conducted Bayesian colocalization analysis integrating GWAS signals with pQTL data from the ROSMAP cohort. Using the ‘coloc’ R package (Giambartolomei et al., 2014), we computed posterior probabilities for five alternative hypotheses (*H*_0_–*H*_4_): no association with either trait (*H*_0_); association with GWAS only (*H*_1_); association with pQTL only (*H*_2_); two distinct causal variants for each trait (*H*_3_); or a single shared causal variant (*H*_4_), and focused on Hypothesis four (*H*_4_) which denotes a single causal variant driving both GWAS and pQTL associations. A threshold (*H*_4_ > 0.8) was adopted, ensuring empirical probability of true colocalization (Zheng et al., 2020).

### Summary-based Mendelian randomization

To validate the results from PWAS and colocalization analysis, SMR was employed (Zhu et al., 2016). This approach performed linear regression on large-scale GWAS loci and pQTLs from ROSMAP and Banner cohorts, estimating the causal relationship between genetic loci and disease with increased statistical power. To detect potential pleiotropy, we applied the heterogeneity in dependent instruments (HEIDI) test, with a *P*_HEIDI_ value of above 0.01 indicating no significant horizontal pleiotropy or linkage effects, thereby supporting the reliability of causal association inference (Chauquet et al., 2021).

### External validation of risk gene expression in iPSC-derived neuronal and astrocytic transcriptomes

To independently validate transcriptional dysregulation of BD risk genes, we analyzed bulk RNA-sequencing data from two independent datasets of iPSC-derived neural cell types. The neuronal dataset included dentate gyrus-like neuronal transcriptomes from six lithium-nonresponsive BD type I patients and eight healthy controls, yielding 36 BD and 52 control neuronal cultures (Santos et al., 2021). The astrocytic dataset included transcriptomes from five BD type I patients and four controls, yielding 19 BD and 14 control astrocyte cultures (Vadodaria et al., 2021). All samples met predefined quality criteria in the original studies, and were used in the present analyses.

Linear mixed models (LMMs) were implemented via the R package ‘lmerTest’ (v3.1–3) (Kuznetsova, Brockhoff, & Christensen, 2017), with target gene expression modeled as a function of the fixed effect *Disease* and random intercept *Donor*, which accounts for donors as the biological replication unit. Model assumptions (normality, homoscedasticity) were validated, with astrocyte gene expression log-transformed to meet distributional requirements. Both datasets were normalized per original study protocols (Santos et al., 2021; Vadodaria et al., 2021) to minimize technical variation.

### Differential expression analysis in postmortem brain tissues

Postmortem hippocampal and PFC tissues from 19 BD patients and 19 unaffected controls were obtained through the University of Pittsburgh Brain Tissue Donation Program (Glausier, Kimoto, Fish, & Lewis, 2015). Key demographic and tissue quality metrics (mean age, postmortem interval, pH, and RNA integrity number) showed no significant intergroup differences. Normalized microarray data, provided by Lanz et al., included 17 BD samples and 19 matched controls for the prefrontal cortex, as well as 18 BD samples and 18 matched controls for the hippocampus, which were utilized in our analysis (Glausier, Kimoto, Fish, & Lewis, 2015; Lanz et al., 2019). To evaluate the cross-diagnostic relevance of the identified risk genes, target gene expression was evaluated in independent postmortem brain tissue cohorts for SCZ (SCZ: *n* = 28; control: *n* = 23) (Maycox et al., 2009) and ASD (ASD: *n* = 16; control: *n* = 16) (Voineagu et al., 2011). Data were processed per original protocols, with identical tests applied as for BD samples. Effect sizes were quantified using Cohen’s *d* to estimate the magnitude of dysregulation independent of sample size.

Differential expression analysis was performed via two-sample, two-tailed *t* tests. For genes mapped to multiple probes, expression values were averaged prior to testing. Given the targeted evaluation of a prespecified candidate gene within independent validation datasets, genes with nominal *P* < 0.05 and consistent directional effects across iPSC-derived neural cell types and brain regions were considered biologically relevant.

### Identification of risk gene-associated modules in iPSC-derived neurons and astrocytes

To systematically identify risk gene-associated co-expression modules, we performed WGCNA on the expression profiles of iPSC-derived neurons and astrocytes using the ‘WGCNA’ R package (v1.73), which is designed for describing gene association patterns between different samples (Langfelder & Horvath, 2008). High-variability genes were selected by retaining transcripts above the first trough in the kernel density distribution of median absolute deviation values. To ensure sample quality control, quantitative outlier detection was employed based on hierarchical clustering, with outliers defined using Tukey’s fences method (Tukey, 1977). All samples were retained, and a soft-thresholding power was determined to achieve scale-free topology (*R* ^2^ > 0.8) while minimizing mean connectivity. An unsigned topological overlap matrix was constructed and subjected to dynamic hybrid tree-cutting with parameters: minModuleSize = 30, mergeCutHeight = 0.25, deepSplit = 2. To validate module robustness, network stability analysis was performed via the sampledBlockwiseModules framework with 10 independent resampling runs and 80% sample retention (Langfelder & Horvath, 2012). Module stability was quantified using the Jaccard index (0.5 as the threshold for preliminary stability) (Hennig, 2007) and a stability score (Monti, Tamayo, Mesirov, & Golub, 2003), defined as the average proportion of genes consistently assigned to the reference module across resampling runs. For target genes-containing modules, we calculated module membership (MM, correlation of gene expression with module eigengene) and gene significance (GS, correlation with BD phenotype). Hub genes were defined as the top 10 genes ranked by intramodular connectivity, followed by validation of their correlations with target genes. All correlations were FDR-corrected (BH method), with significance defined at *P*_FDR_ < 0.05, ensuring robust association signals.

### Functional characterization of risk-associated modules

Building on the WGCNA-derived co-expression modules, we extracted risk gene-containing ones for functional enrichment analysis to further elucidate the molecular mechanisms underlying BD. Specifically, GO (Gene Ontology Consortium, 2021) and KEGG (Kanehisa et al., 2023) pathway analyses were implemented via the ‘Clusterprofiler’ R package (v4.14.6) (Wu et al., 2021). As no custom background gene set was defined, default gene backgrounds comprising all annotated genes in the corresponding GO and KEGG databases were used. Similarly, significance was adjusted using the BH method, with terms retained at a threshold of *P*_FDR_ < 0.05.

### Molecular docking-based druggability screening for top-ranked risk proteins

To further interrogate the feasibility of risk genes and their encoded proteins as therapeutic targets to complement conventional drug-target association databases, we selected a set of representative small-molecule compounds including transcriptomically prioritized candidates, FDA-approved BD reference therapeutics, and pharmacological negative/comparative controls targeting non-BD neurological conditions, based on prior literature describing transcriptional and neurobiological relevance to the prioritized risk gene. Chemical structure files of risk proteins and small-molecule ligands were retrieved from the PDB and PubChem databases, and we executed cavity-detection guided blind docking using the CB-Dock2 tool (Liu et al., 2022). Briefly, a curvature-based cavity detection algorithm was applied to identify the binding pockets of target proteins (Cao & Li, 2014). Protein–ligand blind docking was performed using AutoDock Vina, yielding binding energy values and interaction sites. A threshold of binding energy <−4.0 kcal/mol was adopted to better capture potential pharmacological affinities (Kalasariya et al., 2022; Maiti, Banerjee, & Kanwar, 2022; Wei et al., 2025). Binding interfaces were visualized using Discovery Studio Visualizer and PyMOL. Structural and sequence data from UniProt were used to compare binding sites and define key interaction domains.

## Results

### Cross-dataset convergence identifies high-confidence BD risk genes

Integrative PWAS analysis of ROSMAP and Banner cohorts (*P*_FDR_ < 0.05) identified 73 and 59 brain cis-protein abundance associations with BD, respectively (Supplementary Table S2). Intersection of top-scoring genes revealed eight high-confidence risk genes conserved across both datasets: *DOC2A*, *WIPF3*, *LARS*, *PACSIN2*, *LYRM9*, *CCDC92*, *PDF*, and *DHRS11* (Supplementary Table S3). The consistent identification of these genes across independent cohorts supports their reproducible association with BD-related molecular signals despite cohort-specific differences.

### Colocalization analysis confirms shared causal variants for priority risk genes

To quantify the likelihood of shared causal variants underpinning both GWAS and pQTL signals in BD, we performed Bayesian colocalization analysis with the ROSMAP dataset. Applying a threshold of *H*_*4*_ > 0.8, we identified two prioritized genes – *DOC2A* (*H*_*4*_ = 0.927) and *PACSIN2* (*H*_*4*_ = 0.870) – which also exhibited overlapping support across both ROSMAP and Banner datasets in our PWAS framework. This dual-line evidence strongly reinforced their potential critical roles as BD risk genes.

### Integrative Mendelian randomization unveils *DOC2A* as a key causal gene for BD

**Figure 2.**
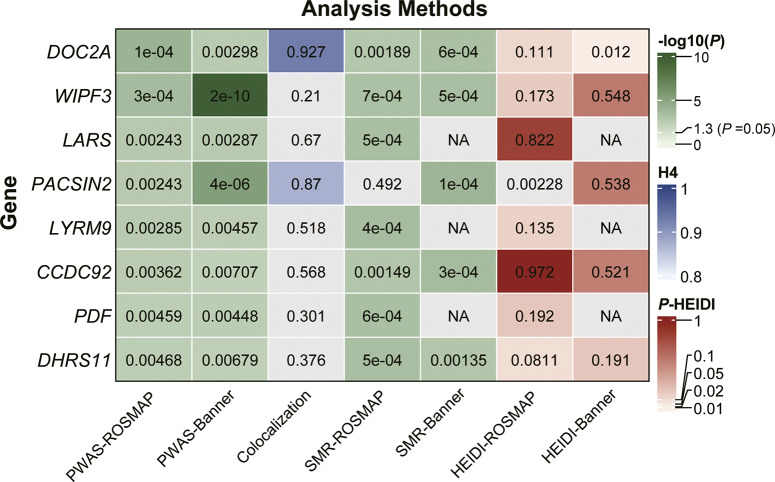
Prioritization of BD risk genes through integrated proteomic and genomic analyses. *DOC2A* emerges as a top-ranked risk gene with robust evidence across PWAS, Bayesian colocalization, SMR, and HEIDI refinement. ‘NA’ indicates nonapplicability of the gene/SNP to the specific analytical pipeline. Figure 2. long description.

We conducted SMR to filter overlapping genes across two datasets under a stringent significance threshold (*P*_SMR_ < 0.05), which were further cross-referenced with identified results derived from PWAS and colocalization analysis. Of particular note, *DOC2A* emerged with consistently supportive evidence across all analytical frameworks (SMR: *P*_ROSMAP_ = 1.89 × 10^−3^, *P*_Banner_ = 6.26 × 10^−4^), with no significant horizontal pleiotropy detected (HEIDI test: *P*_ROSMAP_ = 1.11 × 10^−1^, *P*_Banner_ = 1.20 × 10^−2^), collectively highlighting its pivotal contribution to the pathogenesis of BD (Figure 2).

### Cell- and region-specific dysregulation of *DOC2A* in BD pathophysiology

**Figure 3.**
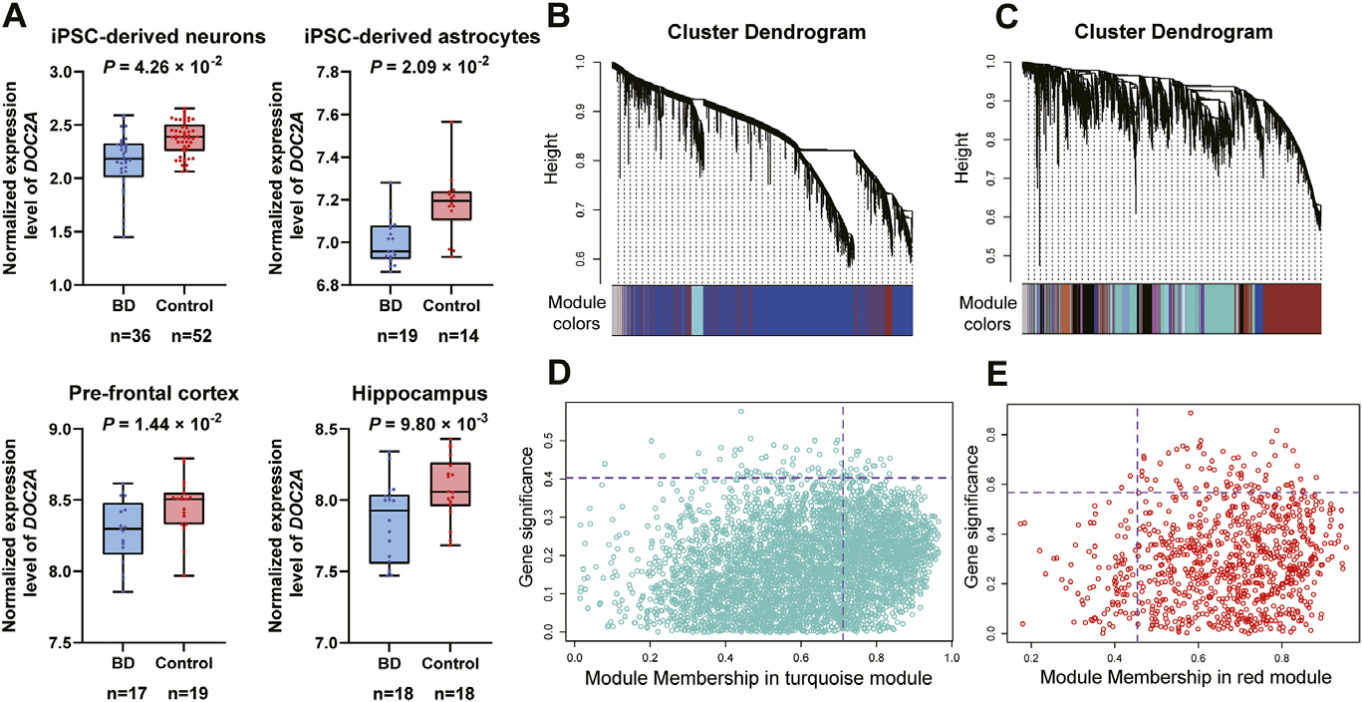
Disease-associated dysregulation and co-expression networks anchored by *DOC2A.* (A) Significant downregulation of *DOC2A* in BD-relevant cell types (iPSC-derived neurons/astrocytes) and brain regions (PFC/hippocampus). For iPSC data, statistical significance was determined using Linear Mixed Models. (B, C) Gene co-expression modules derived from neuronal (B) and astrocytic (C) transcriptomes. (D, E) *DOC2A* localizes within synaptic modules showing strongest disease/module association (D: neuronal; E: astrocytic). Figure 3. long description.

As neurons and astrocytes play pivotal roles in nervous system physiology, we integrated gene transcriptomic data across both cell types to evaluate the pathogenic contributions of risk genes in BD etiology. Using LMMs accounting for donor structure, *DOC2A* downregulation was detected in neurons (*P* = 4.26 × 10^−2^) and astrocytes (log-transformed *P* = 2.09 × 10^−2^) (Figure 3A; Supplementary Table S6). As supporting evidence, a similar downregulation pattern was observed in hippocampus (*P* = 9.80 × 10^−3^, *t* = −2.738, *d* = −0.91) and PFC (*P* = 1.44 × 10^−2^, *t* = −2.580, *d* = −0.86), suggesting transcriptional suppression of *DOC2A* as a putative critical contributor to BD through reduced protein abundance. In contrast, *DOC2A* showed no significant dysregulation in SCZ (*P* = 1.49 × 10^−1^, *t* = −1.467, *d* = −0.41) and ASD (*P* = 4.38 × 10^−1^, *t* = −0.786, *d* = −0.28) cohorts, highlighting that the magnitude of its downregulation is more pronounced in BD.

### Linking *DOC2A*-associated co-expression networks to synaptic pathways

To identify co-expression modules linked to risk genes, we performed WGCNA. Based on the average link hierarchical clustering and optimized threshold power (Supplementary Figure S4), we identified 47 modules (Figure 3B) in neuronal and 66 modules (Figure 3C) in astrocytic datasets, with *DOC2A*, respectively, located in the turquoise and red modules. In both contexts, *DOC2A* exhibited strong associations with the module and the BD phenotype (neuron: MM = 0.71, *P* = 1.90 × 10^−23^; GS = −0.40, *P* = 2.19 × 10^−5^; astrocyte: MM = 0.46, *P* = 2.90 × 10^−2^; GS = −0.57, *P* = 8.41 × 10^−3^) (Figure 3D, E). Moreover, *DOC2A* showed robust correlations with all hub genes (defined as the top 10 genes ranked by intramodular connectivity) in neuron (e.g. *STXBP1*) and five hub genes in astrocyte (e.g. *CD81*) (Supplementary Table S8), implying its potential regulatory relevance to core functional hubs within these modules. Stability analysis confirmed the *DOC2A*-associated Turquoise module in iPSC-derived neurons had relatively high stability (Jaccard Index = 0.549 > 0.5; Stability Score = 0.668), validating the reliability of core findings. By contrast, the Red module in astrocytes exhibited markedly lower stability (Jaccard Index = 0.254; Stability Score = 0.320), indicative of a less robust co-expression architecture.

**Figure 4.**
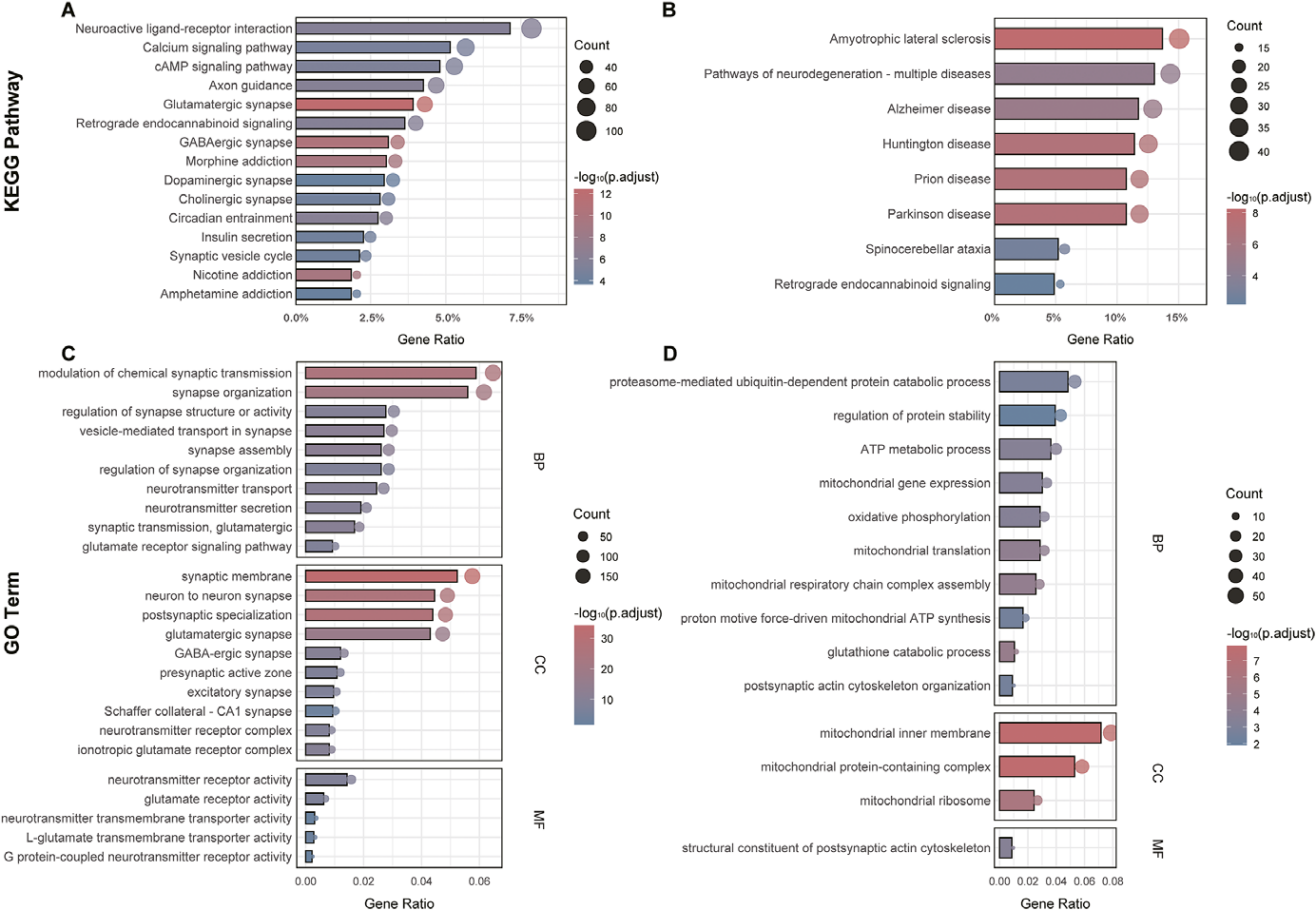
Functional enrichment of *DOC2A*-associated co-expression modules. KEGG pathways and Gene Ontology (GO) terms enriched for neuronal (A, C) and astrocytic (B, D) modules converge on synaptic function regulation. BP: Biological Processes; CC: Cellular Components; MF: Molecular Functions. Figure 4. long description.

KEGG and GO analyses further delineate molecular mechanisms underlying these modules, prioritizing functional terms closely relevant to neuropsychiatric pathophysiology (Supplementary Tables S9–S12). In terms of biological pathways, KEGG analysis highlighted pathways including neuroactive ligand–receptor interaction, glutamatergic synapse, calcium signaling pathway, dopaminergic synapse, and neurodegeneration pathways (Figure 4A, B). For functional attributes, GO analysis similarly identified neuropsychiatric-relevant biological processes, notably synaptic transmission, glutamatergic synapse, regulation of synapse organization, neurotransmitter secretion, postsynaptic actin cytoskeleton organization, and mitochondrial gene expression (Figure 4C, D). These results collectively advanced our understanding of the roles of *DOC2A* and its co-expressed genes in the pathophysiology of BD.

### DOC2A as a synaptic ligand hub links to BD pathology

**Figure 5.**
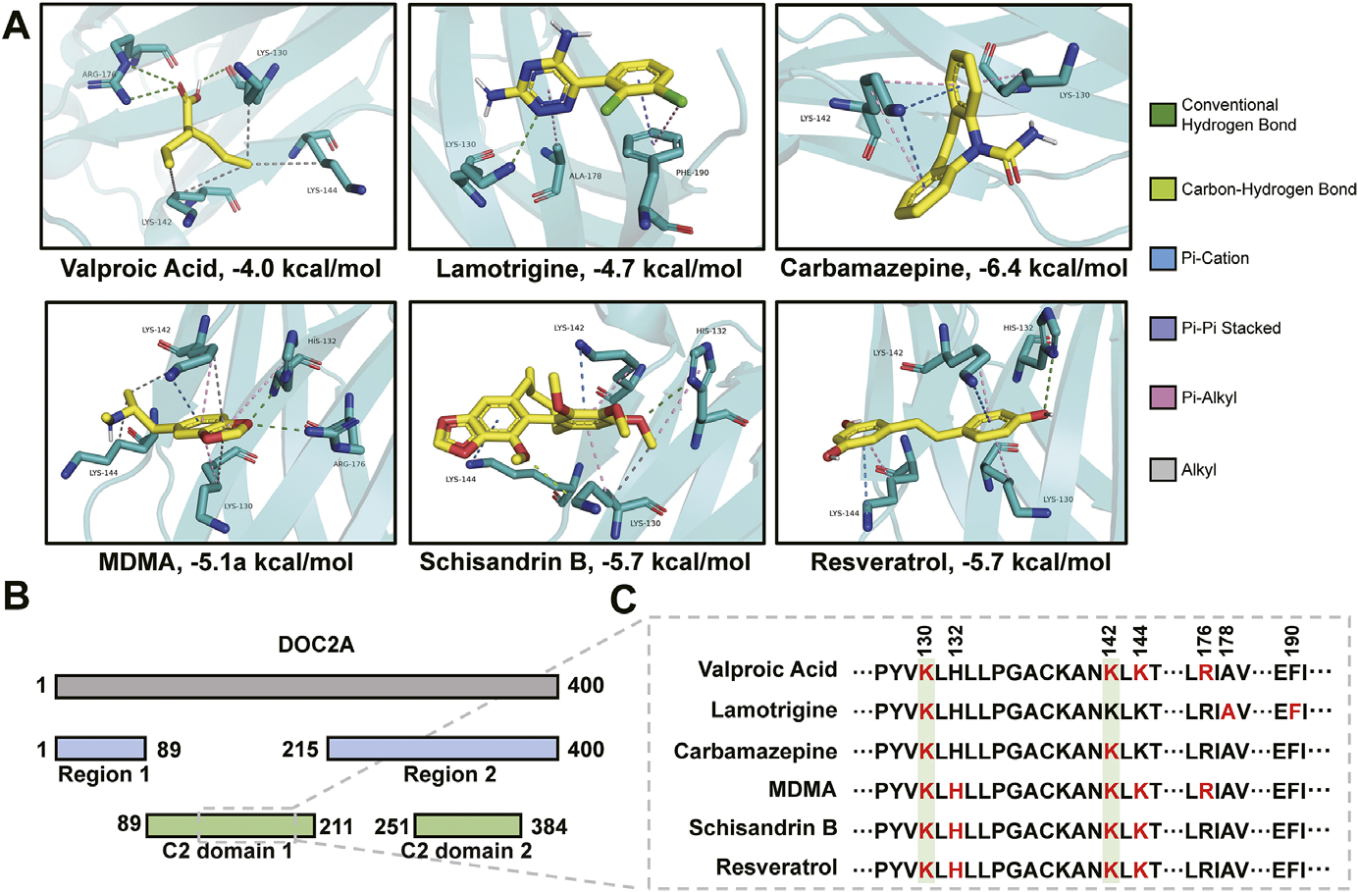
Molecular docking reveals potential binding affinity between DOC2A and multiple neuroactive compounds. (A) High-affinity binding of BD therapeutics and neuroprotective compounds to DOC2A, with the binding energy (kcal/mol) for each pair shown below the image. (B) Domain architecture of DOC2A: Main chain (gray), protein–protein interaction interfaces (blue), C2 domains (green). (C) Sequence alignment of interactive amino acid residues, with binding sites marked in red, conserved sites in green. Figure 5. long description.

Molecular docking revealed high-affinity binding of FDA-approved BD therapeutics – valproic acid, lamotrigine, and carbamazepine (Nierenberg et al., 2023; Ramadan et al., 2025) – to DOC2A (domain 1) (Figure 5A), suggesting that DOC2A may function as a potential ligand-interacting node within BD-relevant synaptic networks. This finding gains further support from the similarly strong binding of three psychoactive compounds – N-methyl-3,4-methylenedioxyamphetamine (MDMA), schisandrin B, and resveratrol – suggesting that DOC2A may represent a convergence point for diverse therapeutic classes with shared neuroprotective mechanisms. Notably, several of these agents (valproic acid, MDMA, schisandrin B, and resveratrol) have previously been shown to modulate *DOC2A* expression levels in neuronal systems (Eun et al., 2010; Schulpen, Pennings, & Piersma, 2015; Tomé-Carneiro et al., 2013; Zhang et al., 2019), reinforcing its putative functional relevance in BD pathology.

Binding site profiling further demonstrated striking spatial overlap: all ligands interacted with highly similar amino acid residues within DOC2A’s C2 domain 1 (Figure 5B, C), a critical functional module of interaction. In particular, lysine residues K130 and K142 emerged as conserved binding anchors across multiple ligand–receptor complexes. These residues may constitute regulatory switches of DOC2A activity, providing mechanistic insight into how pharmacological modulation of DOC2A might influence psychiatric disorders.

In contrast, the negative control (antiepileptic drug) levetiracetam showed negligible binding affinity to DOC2A in the same pocket (binding energy = −3.7 kcal/mol), while the comparative control topiramate (antiepileptic drug) (binding energy = −4.5 kcal/mol) bound to distinct residues (Y128, K142, and K144), which further identifies K130 as a conserved binding anchor (Supplementary Table S13).

## Discussion

Our study nominates *DOC2A* as a candidate risk gene for BD, supported by convergent genetic, proteomic and network-level associations. PWAS, Bayesian colocalization, and SMR analyses collectively provide statistical genetic evidence supporting the association between *DOC2A* and BD. Transcriptomic profiling revealed consistent *DOC2A* downregulation across iPSC-derived neurons/astrocytes and postmortem PFC/hippocampus, indicating cell-type and regional dysregulation. *DOC2A* is significantly associated with synaptic co-expression modules enriched for neurotransmitter release, postsynaptic organization, and mitochondrial metabolism – processes putatively central to BD. Critically, molecular docking reveals high-affinity binding of DOC2A to BD-related therapeutics and neuroprotective compounds (e.g. valproic acid), nominating it as a mechanistically grounded, druggable candidate target.

DOC2A, a brain-specific calcium sensor belonging to the dual C2-domain protein family, is enriched at synaptic vesicles (Orita et al., 1995; Verhage et al., 1997; Yao, Gaffaney, Kwon, & Chapman, 2011) and plays a central role in regulating neurotransmitter release, synaptic efficacy, and plasticity (Ramirez et al., 2017; Sakaguchi et al., 1999; Q.-W. Wang et al., 2023; Xiao et al., 2022) – processes tightly linked to BD pathophysiology (Elvsåshagen et al., 2012; Valstad et al., 2021). Although previously implicated in suicidality, epilepsy and other disorders (Hu, Tang, Lan, & Mi, 2019; Jin et al., 2024; Zhao et al., 2024; Zhou et al., 2023), DOC2A has received little attention in the context of BD. Notably, copy number variations in its genomic region are significantly enriched in BD cohorts (Steinberg et al., 2014), reinforcing its plausible but underexplored role in disease pathogenesis.

Mechanistically, DOC2A regulates neurotransmitter release through its interactions with Munc13-1 (UNC13A) and Munc18-1 (STXBP1), two core components of the synaptic vesicle priming and fusion machinery (Ma et al., 2013; Stepien & Rizo, 2021; X. Wang et al., 2020). DOC2A binds Munc13-1 to cooperatively regulate vesicle priming and synaptic function (Mochida et al., 1998; Orita et al., 1997), while also directly interacting with Munc18-1 to alleviate its inhibitory constraint on syntaxin, thereby facilitating vesicle docking and fusion (Verhage et al., 1997). Diacylglycerol (DAG), a lipid second messenger, has been shown to enhance DOC2A–Munc13-1 binding (Orita et al., 1997), activate Munc13-1, and phosphorylate Munc18-1 (Barclay et al., 2003; de Jong et al., 2016), amplifying the DOC2A–Munc13-1–Munc18-1 signaling axis. This is particularly relevant in BD, where lithium – a first-line mood stabilizer – increases DAG levels (Thiruvengadam, 2023), and DGKH, the major DAG-degrading enzyme, is upregulated (Moya, Murphy, McMahon, & Wendland, 2010). Together, these findings suggest that this synaptic axis may represent a key neurobiological mechanism through which DOC2A contributes to BD.

Our co-expression analysis in neurons corroborates this hypothesis: *DOC2A* was co-expressed with *STXBP1* and *UNC13A* within the same module, with strong correlations and consistent downregulation observed in BD samples (Supplementary Table S6). Functional enrichment analysis confirmed the module’s involvement in the synaptic vesicle cycle, neurotransmitter secretion, and chemical synaptic transmission. Notably, *STXBP1* emerged as a central hub, suggesting that *DOC2A* may exert its regulatory influence on synaptic function primarily via *STXBP1*-centered networks.

Building upon evidence linking *DOC2A* deficiency to disrupted dendritic morphology (Q.-W. Wang et al., 2023) – a synaptic structure critically dependent on the postsynaptic cytoskeleton (Cornelius, Rottner, Korte, & Michaelsen-Preusse, 2021; Sekino, Kojima, & Shirao, 2007) and regulated by astrocytes through their secretory and mitochondrial functions (Chen et al., 2023; Doliwa et al., 2025; Jackson & Robinson, 2018; Rose et al., 2020) – we investigated its broader synaptic regulatory role in iPSC-derived astrocytes: *DOC2A* showed strong correlations with hub genes (e.g. *CD81*, a BD-associated astrocyte-derived exosomal marker (Attili et al., 2020). The co-expression module was enriched in pathways, including postsynaptic actin cytoskeleton organization, ATP metabolic process, and mitochondrial gene expression. However, the astrocytic module showed lower network stability than the neuronal module in resampling validation. This may reflect inherent astrocytic state heterogeneity or a more transient regulatory role of *DOC2A* in astrocytes, so interpretations of specific astrocytic hub interactions require caution and further large-scale validation.

Collectively, these findings suggest that *DOC2A* may act as a potential regulator involved in dual processes relevant to BD pathogenesis: regulating synaptic vesicle trafficking in neurons and modulating astrocyte-mediated synaptic support through mitochondrial function and secretory regulation.

Notably, many of these biological processes identified above are broadly implicated in neuropsychiatric conditions featuring synaptic dysfunction. While our cross-diagnostic analysis revealed that *DOC2A*’s downregulation was more pronounced in BD within the examined cohorts, these preliminary observations should be interpreted with caution as the modest sample sizes of the SCZ and ASD groups may limit the statistical power to detect subtle dysregulation, and *DOC2A*-centered networks may still underpin shared endophenotypes across traditional diagnostic boundaries. Future studies with larger transdiagnostic cohorts and high-resolution single-cell data are needed.

Valproic acid, lamotrigine, and carbamazepine – first-line treatments for BD – are well established to modulate neurotransmitter release, particularly through GABAergic and glutamatergic systems (Z. Liu et al., 2015; Post, 2004; Schloesser, Martinowich, & Manji, 2012; Sitges, Chiu, & Reed, 2016; Sitges, Guarneros, & Nekrassov, 2007; Yoshida, Okada, Zhu, & Kaneko, 2007). Our molecular docking revealed high-affinity binding of all three to DOC2A within its C2 domain 1, a calcium-sensing region essential for vesicular trafficking (Groffen et al., 2006; Yao, Gaffaney, Kwon, & Chapman, 2011). This suggests that, beyond classical receptor-level modulation, these agents may converge upstream at the level of vesicle dynamics.

As an extension, several emerging neuropsychiatric compounds – MDMA, schisandrin B, and resveratrol – also exhibited strong binding to this domain, at sites overlapping those of FDA-approved mood stabilizers. These compounds have been shown to enhance mood, cognition or neurogenesis (Giridharan et al., 2011; Menegas et al., 2023; Mithoefer, Grob, & Brewerton, 2016; Shayganfard, 2020; Sun et al., 2014; Torrado Pacheco & Moghaddam, 2024), and are reported to modulate *DOC2A* expression (Bandiwadekar et al., 2025; Eun et al., 2010; Zhang et al., 2019), reinforcing its therapeutic relevance. This dual convergence of structural affinity and transcriptomic regulation indicates a mechanistically tractable path for therapeutic repurposing in BD. Particularly intriguing is the convergence at K130 and K142, which may represent conserved regulatory anchors for precision-targeted intervention. Unlike the minimal interaction of levetiracetam or the divergent binding pattern of topiramate, two antiepileptic agents widely used for non-BD neurological conditions, the unique convergence of prioritized BD-relevant agents at K130 further highlights K130 as a mechanistically relevant target site for future structure-guided drug design. These findings position DOC2A not only as a shared pharmacological interface, but also as a strategic entry point for next-generation drug development in BD and related psychiatric disorders. Nevertheless, broader comparative evaluation across expanded compound libraries remains an important direction. Future studies incorporating a broader range of pharmacologically unrelated compounds may help further delineate the specificity and generalizability of these interaction patterns.

This study has several strengths. First, we used a three-stage analytical framework (PWAS, colocalization, and SMR) to integrate multi-ancestry GWAS data with region-specific pQTL datasets, identifying high-confidence risk genes. Second, transcript validation across brain regions and cell types showed convergent *DOC2A* dysregulation, reinforcing its pathogenic relevance. Finally, mechanistic exploration characterized *DOC2A*-associated gene networks and functions, while molecular docking supported its druggability as a therapeutic target.

Our study has limitations. Despite including different GWAS cohorts, imbalanced distributions of ancestry, age, and sex may introduce residual confounding. The pQTL data are restricted by original sample sources, potentially biasing PWAS and differential expression analysis due to tissue-specific expression patterns. The WGCNA analyses were conducted as exploratory functional contextualization, and the present emphasis was placed on the potential regulatory influence of *DOC2A* on module organization, whereas its intrinsic centrality within co-expression networks warrants further systematic evaluation in future studies. In addition, the neuronal iPSC cohort used for external validation is limited to lithium-nonresponsive BD type I patients, a narrow clinical subgroup that does not represent the broader BD population. This means the observed *DOC2A* downregulation may be more closely linked to lithium nonresponse, thus restricting the generalizability of this finding. Finally, limited statistical power in large-scale analyses may have resulted in the omission of key proteins lacking cross-tier evidence, and further molecular mechanistic studies are needed.

In summary, we identify *DOC2A* as a promising risk gene for BD. Our findings reveal that *DOC2A* in neurons and astrocytes may exert neuroprotective effects by potentially mediating vesicle trafficking and neurotransmitter release, and support its potential as a novel pharmacological target. These findings offer new insights into the pathogenic mechanisms of BD and facilitate clinical translation for therapeutic development.

## Supporting information

10.1017/S0033291726104565.sm001 Yuan et al. supplementary material Yuan et al. supplementary material

## Data Availability

The data used in the study can be accessed and downloaded from original studies (Glausier, Kimoto, Fish, & Lewis, 2015; Lanz et al., 2019; Maycox et al., 2009; O’Connell et al., 2025; Santos et al., 2021; Vadodaria et al., 2021; Voineagu et al., 2011; Wingo et al., 2021). The computational scripts and analytical workflows of this study can be found in a publicly accessible repository: https://github.com/CY1479/BD_Project

## Supplementary material

The supplementary material for this article can be found at http://doi.org/10.1017/S0033291726104565.

## Figure 1. Long description

From the left, BD G W A S data (158,036 BD cases, 2,796,499 controls) and brain p Q T L data (R O S M A P: 376 d l P F C samples, Banner: 152 d l P F C samples) feed into proteomic putative causal gene prioritization. P W A S identifies overlapping genes in R O S M A P and Banner: D O C 2 A, W I P F 3, L A R S, P A C S I N 2, L Y R M 9, C C D C 92, P D F, D H R S 11. Colocalization analysis highlights genes with shared causal variants: D O C 2 A and P A C S I N 2. Causal inference refinement uses S M R (D O C 2 A) and H E I D I test (D O C 2 A, no significant horizontal pleiotropy). Transcriptomic dysregulation is assessed by differential expression, showing downregulation of D O C 2 A in BD brain regions and cell types, with cross-diagnostic comparison (S C Z, A S D). Brain transcriptional data includes hippocampus (18 BD, 18 controls) and frontal cortex (17 BD/19 controls, 28 S C Z/23 controls, 16 A S D/16 controls). Cellular transcriptional data covers i P S C-derived neurons (36 BD/52 controls, 6 BD/8 donors) and astrocytes (19 BD/14 controls, 5 BD/4 donors). Functional pathway elucidation involves co-expression network construction via W G C N A, identifying D O C 2 A as a potential regulator, and functional enrichment of co-expression module genes (BD-relevant synaptic pathways in neuron and astrocyte). Druggable target validation uses molecular docking, revealing high-affinity binding with BD therapeutics and neuroprotective compounds, conserved binding anchor (K130/K142), and a potential synaptic ligand hub and candidate therapeutic target for BD.

Navigate back to Figure 1..

## Figure 2. Long description

Starting at the top-left, the matrix lists genes vertically: D O C 2 A, W I P F 3, L A R S, P A C S I N 2, L Y R M 9, C C D C 92, P D F, D H R S 11. Analysis methods are labeled horizontally: P W A S dash R O S M A P, P W A S dash Banner, Colocalization, S M R dash R O S M A P, S M R dash Banner, H E I D I dash R O S M A P, H E I D I dash Banner. Each cell contains a numerical value, colored by three scales: green for negative log ten of P, blue for H four, red for P dash H E I D I. D O C 2 A shows strong evidence across all methods, with values: 1 e minus 4, 0.00298, 0.927, 0.00189, 6 e minus 4, 0.111, 0.012. W I P F 3 has a standout value of 2 e minus 10 in P W A S dash Banner. L A R S and P A C S I N 2 have N A in some columns. Color legends at the right indicate green for high negative log ten of P, blue for high H four, and red for high P dash H E I D I. N A denotes nonapplicability for certain gene-method combinations.

Navigate back to Figure 2..

## Figure 3. Long description

Panel A contains four box plots comparing normalized expression levels of D O C 2 A between B D and control groups in iPSC-derived neurons, iPSC-derived astrocytes, pre-frontal cortex, and hippocampus. Each plot includes sample sizes and p-values: neurons (n=36 B D, n=52 control, p=4.26 x 10 super -2), astrocytes (n=19 B D, n=14 control, p=2.09 x 10 super -2), pre-frontal cortex (n=17 B D, n=19 control, p=1.44 x 10 super -2), hippocampus (n=18 B D, n=18 control, p=9.80 x 10 super -3). Panel B shows a cluster dendrogram for neuronal transcriptomes with module colors below, mostly blue and turquoise. Panel C displays a cluster dendrogram for astrocytic transcriptomes with module colors, including blue, turquoise, and red. Panel D is a scatter plot of gene significance versus module membership in the turquoise module, with most points clustered below 0.5 gene significance and 0.8 module membership. Panel E is a scatter plot of gene significance versus module membership in the red module, with points distributed up to 0.8 gene significance and 0.8 module membership. D O C 2 A is localized within synaptic modules showing strong disease association.

Navigate back to Figure 3..

## Figure 4. Long description

Starting at the top-left, panel A displays KEGG pathway enrichment for neuronal modules, with horizontal bars for pathways such as neuroactive ligand-receptor interaction, calcium signaling, and c A M P signaling. The x-axis is gene ratio, ranging from zero percent to seven point five percent. Bar color intensity reflects negative log p adjust values, from four to twelve, and circle size indicates gene count, from forty to one hundred. Panel B, top-right, shows astrocytic module KEGG pathway enrichment, with bars for amyotrophic lateral sclerosis, pathways of neurodegeneration, Alzheimer disease, Huntington disease, prion disease, Parkinson disease, spinocerebellar ataxia, and retrograde endocannabinoid signaling. Gene ratio ranges from zero percent to fifteen percent, negative log p adjust from four to eight, and gene count from fifteen to forty. Panel C, bottom-left, presents Gene Ontology term enrichment for neuronal modules, divided into biological processes, cellular components, and molecular functions. Biological processes include modulation of chemical synaptic transmission, synapse organization, regulation of synapse structure or activity, vesicle-mediated transport in synapse, synapse assembly, regulation of synapse organization, neurotransmitter transport, neurotransmitter secretion, synaptic transmission, glutamatergic, and glutamate receptor signaling pathway. Cellular components feature synaptic membrane, neuron to neuron synapse, postsynaptic specialization, glutamatergic synapse, G A B A-ergic synapse, presynaptic active zone, excitatory synapse, Schaffer collateral C A one synapse, neurotransmitter receptor complex, ionotropic glutamate receptor complex. Molecular functions include neurotransmitter receptor activity, glutamate receptor activity, neurotransmitter transmembrane transporter activity, L-glutamate transmembrane transporter activity, and G protein-coupled neurotransmitter receptor activity. Gene ratio ranges from zero to zero point zero six, negative log p adjust from ten to thirty, and gene count from fifty to one hundred fifty. Panel D, bottom-right, shows astrocytic module Gene Ontology enrichment, with biological processes such as proteasome-mediated ubiquitin-dependent protein catabolic process, regulation of protein stability, A T P metabolic process, mitochondrial gene expression, oxidative phosphorylation, mitochondrial translation, mitochondrial respiratory chain complex assembly, proton motive force-driven mitochondrial A T P synthesis, glutathione catabolic process, postsynaptic actin cytoskeleton organization. Cellular components include mitochondrial inner membrane, mitochondrial protein-containing complex, mitochondrial ribosome. Molecular function is structural constituent of postsynaptic actin cytoskeleton. Gene ratio ranges from zero to zero point zero eight, negative log p adjust from two to seven, and gene count from ten to fifty. Color gradients and circle sizes consistently represent negative log p adjust and gene count across all panels.

Navigate back to Figure 4..

## Figure 5. Long description

Panel A contains six molecular docking visualizations arranged in two rows and three columns. Each subpanel shows a neuroactive compound (Valproic Acid, Lamotrigine, Carbamazepine, M D M A, Schisandrin B, Resveratrol) docked to DOC2A, with the ligand in yellow and interacting amino acids labeled (e.g., L Y S 130, L Y S 142, H I S 132, A R G 176). Interaction types are color-coded: green for conventional hydrogen bonds, yellow for carbon-hydrogen bonds, blue for pi-cation, purple for pi-alkyl, gray for alkyl, and light blue for pi-pi stacked. Binding energies are listed below each compound: Valproic Acid negative 4.0 kcal per mol, Lamotrigine negative 4.7 kcal per mol, Carbamazepine negative 6.4 kcal per mol, M D M A negative 5.1 kcal per mol, Schisandrin B negative 5.7 kcal per mol, Resveratrol negative 5.7 kcal per mol. Panel B shows the linear domain architecture of DOC2A from residue 1 to 400, with the main chain in gray, two protein-protein interaction regions in blue (Region 1: 1 to 89, Region 2: 215 to 400), and two C2 domains in green (C2 domain 1: 89 to 211, C2 domain 2: 251 to 384). Panel C presents a sequence alignment of interactive residues for each compound, highlighting binding sites in red and conserved sites in green (K at positions 130, 142). The alignment shows that all compounds interact with the same core motif P Y V K L H L L P G A C K A N K L K T.

Navigate back to Figure 5..
