# Supplementary material for: Integrative multi-omics identifies DOC2A as a novel pharmacological target for bipolar disorder
Source: Psychol Med. 2026 May 28;56:e162. doi: 10.1017/S0033291726104565 (PMC13234528; doi:10.1017/S0033291726104565)
Supplement: Yuan et al. supplementary material [file S0033291726104565sup001.docx]

**Contents**

**Figure S1 Median absolute deviation (MAD) distribution for gene selection in tissue-specific datasets**

**Figure S2 Hierarchical clustering of samples integrated with trait heatmaps**

**Figure S3 Soft-threshold power selection based on scale-free topology model fit and mean connectivity**

**Figure S4 Module-trait association heatmaps across phenotypes**

**Table S1 Data resources and analytical specifications for the multi-omics study**

**Table S2 Significant risk genes identified by PWAS**

**Table S3 Genetic information and PWAS results for overlapping genes**

**Table S4 Detailed information of colocalization analysis between variants and overlapping genes**

**Table S5 Genetic information and SMR results for overlapping genes**

**Table S6 Transcriptional data sources and results for differential expression analysis**

**Table S7 Co-expressed genes of *DOC2A* identified by WGCNA**

**Table S8 Correlation between hub genes of co-expression module and *DOC2A***

**Table S9 KEGG enrichment results in neuron dataset**

**Table S10 GO enrichment results in neuron dataset**

**Table S11 KEGG enrichment results in astrocyte dataset**

**Table S12 GO enrichment results in astrocyte dataset**

**Table S13 Molecular docking results of DOC2A with neuroactive compounds**

**Figure S1 Median Absolute Deviation (MAD) Distribution for Gene Selection in tissue-specific datasets**


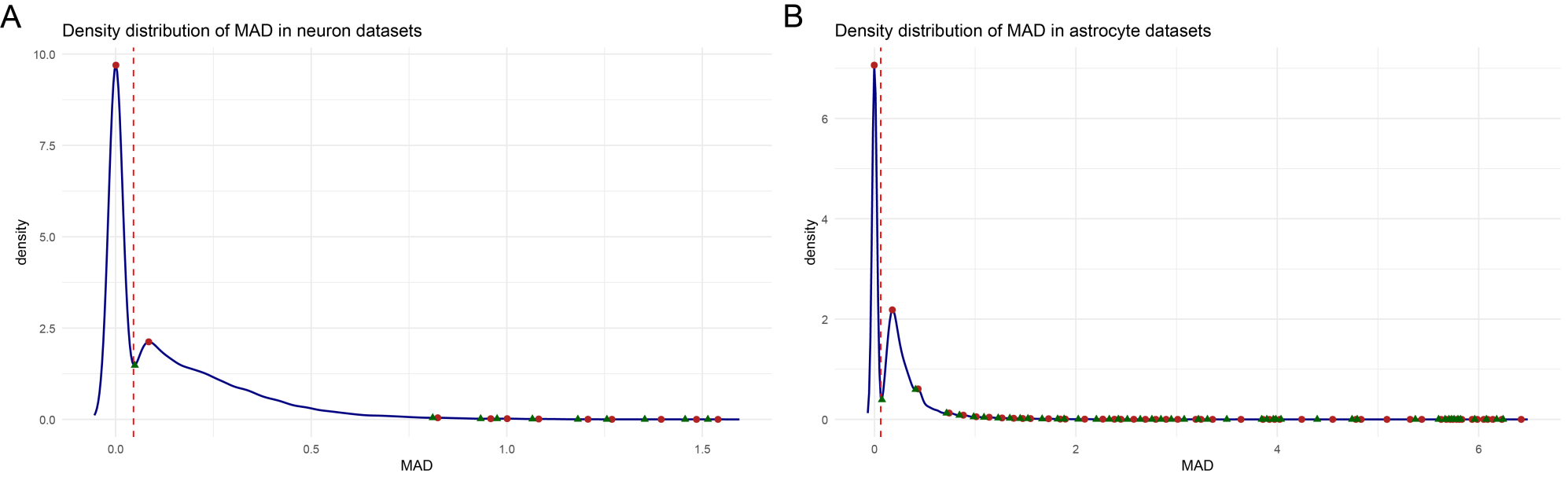


1. Density distribution of MAD in neuron datasets. (B) Density distribution of MAD in astrocyte datasets. The red dots represent peaks while the green triangles represent troughs. Vertical dashed lines indicate density trough-based cutoffs. Genes above the cutoff were retained for subsequent co-expression network analysis.

**Figure S2 Hierarchical Clustering of Samples Integrated with Trait Heatmaps**


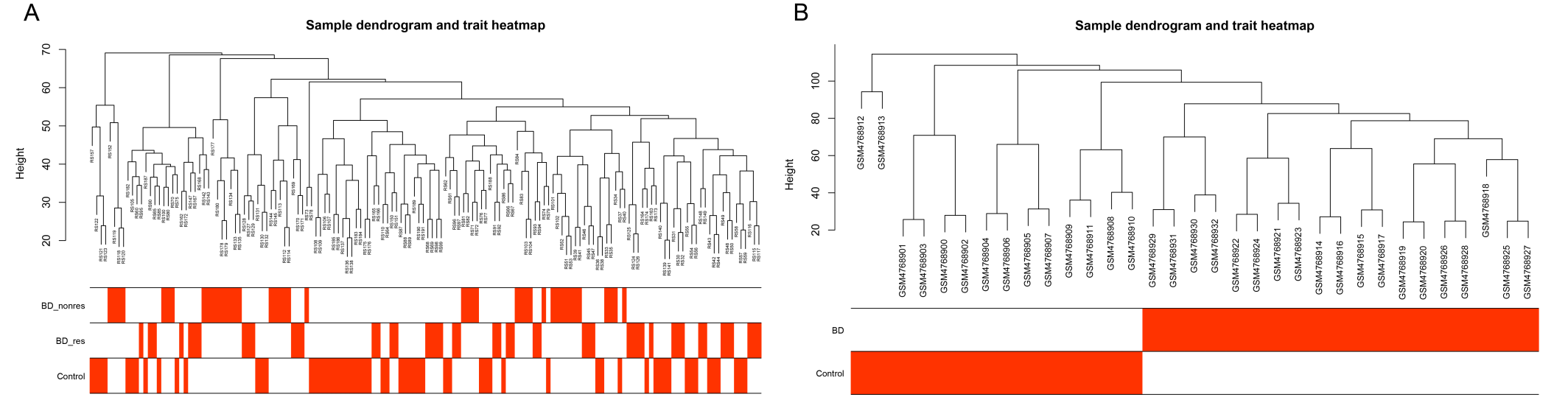


(A) Neuronal dataset. (B) Astrocytic dataset. Tukey’s fences method was used for outlier detection, with cut-off thresholds of 61.15 (A) and 136.62 (B). All samples were below the corresponding threshold. Dendrogram branches reflect sample clustering based on gene expression similarity. The trait heatmap reflects the different phenotypes corresponding to each sample.

**Figure S3 Soft-threshold power selection based on scale-free topology model fit (R²) and mean connectivity**


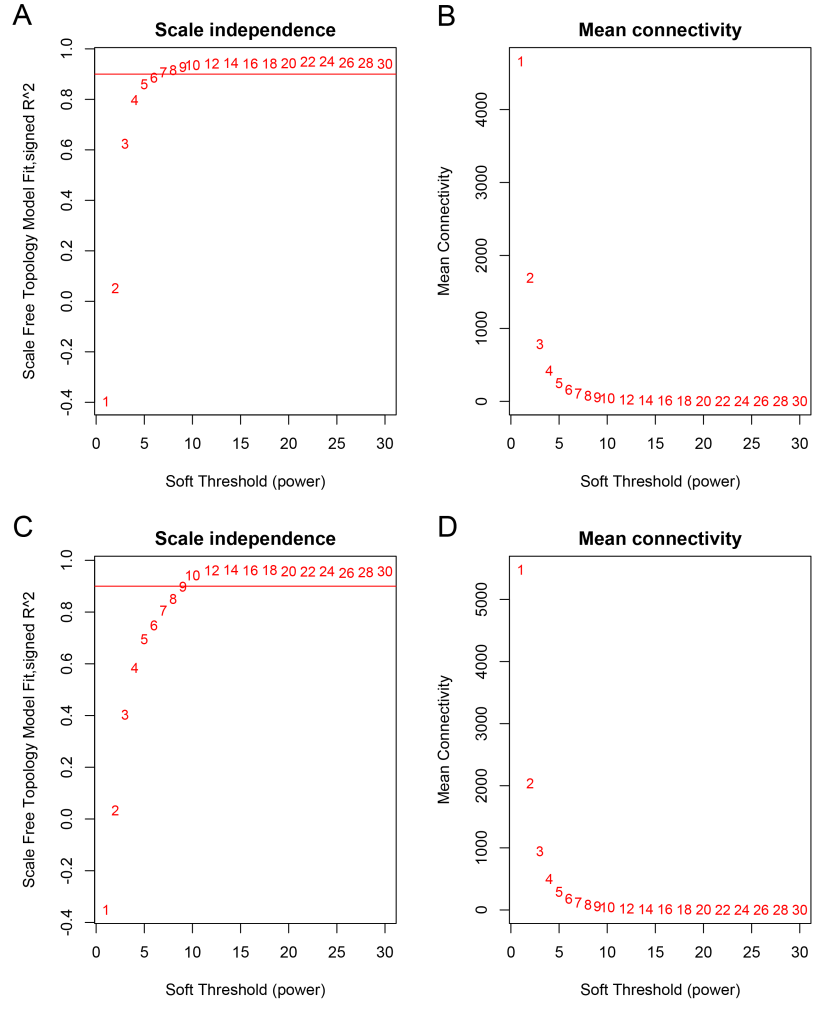


(a, b) represent results of the neuron dataset, with the power of 7 used, while (c, d) represent results of the astrocyte dataset, with the power of 9 used. (a, c) Scale independence plots showing the signed R² of the scale-free topology model fit against soft threshold (power). (b, d) Mean connectivity plots showing the mean connectivity against soft threshold (power).

**Figure S4 Module-Trait Association Heatmaps Across Phenotypes**


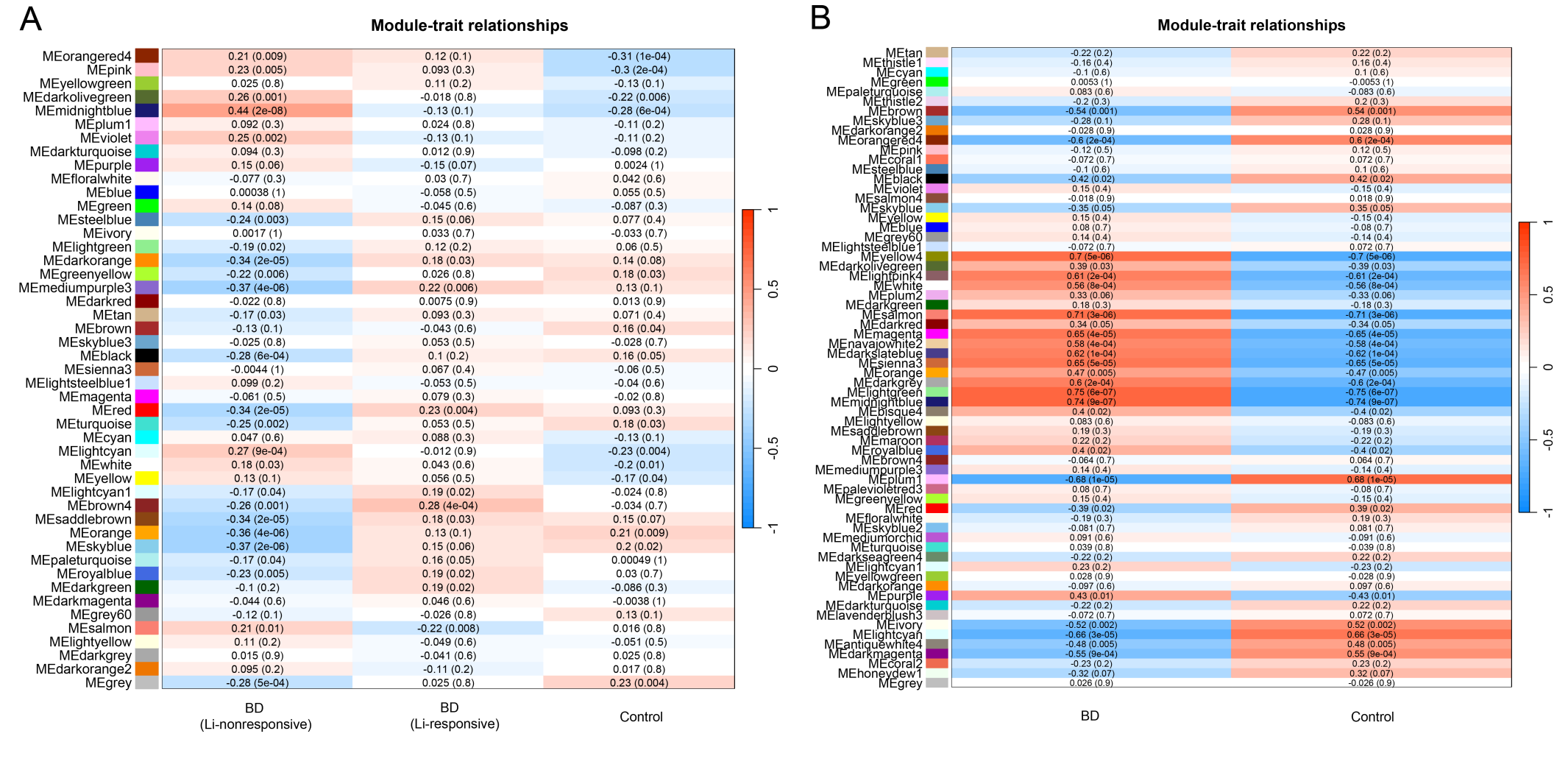


1. Neuronal co-expression modules. (b) Astrocytic co-expression modules. Each row represents a module, and each column represents a phenotype. The color indicates the correlation coefficient (red for positive, blue for negative), with correlation and *P*-values in each cell.

**Table S1 Data resources and analytical specifications for the multi-omics study**

| Data resource / Accession | Data modality | Analytical sample size | Role in current study | Original reference (PMID) |
| --- | --- | --- | --- | --- |
| BD GWAS meta-analysis | GWAS summary statistics | 158,036 cases; 2,796,499 controls (effective sample size) | Genetic association framework; input for PWAS, SMR and colocalization analyses | 39843750 |
| ROSMAP DLPFC brain proteome | Brain pQTL reference panel | n = 376 | Protein weight reference for PWAS | 29865057; 33510477 |
| Banner DLPFC brain proteome | Brain pQTL reference panel | n = 152 | Independent protein weight reference for PWAS replication | 25619230; 33510477 |
| iPSC-derived neuronal transcriptomes (dentate gyrus-like neurons) | Bulk RNA-seq | 36 BD cultures; 52 control cultures | Targeted transcriptional validation; WGCNA network analysis | 33398088 |
| iPSC-derived astrocytic transcriptomes | Bulk RNA-seq | 19 BD cultures; 14 control cultures | Targeted transcriptional validation; co-expression network analysis | 33667413 |
| Postmortem PFC microarray | Brain transcriptomics | 17 BD; 19 controls | Independent regional expression validation | BD: 31123247 |
|  |  | 28 SCZ; 23 controls |  | SCZ: 19255580 |
| Postmortem frontal cortex microarray | Brain transcriptomics | 16 ASD; 16 controls | Independent regional expression validation | 21614001 |
| Postmortem hippocampus microarray | Brain transcriptomics | 18 BD; 18 controls | Independent regional expression validation | 31123247 |

**Table S2 Significant risk genes identified by PWAS**

| Gene | ROSMAP | | | Banner | | |
| --- | --- | --- | --- | --- | --- | --- |
|  | Z-PWAS | P-PWAS | P-FDR | Z-PWAS | P-PWAS | P-FDR |
| ENSG00000122574.WIPF3 | 4.7926 | 0.00000165 | 0.000301 | 7.3733 | 1.66E-13 | 1.89E-10 |
| ENSG00000100266.PACSIN2 | 4.2004 | 0.0000266 | 0.002429 | 5.709 | 1.14E-08 | 0.00000434 |
| ENSG00000133706.LARS | -4.212 | 0.0000253 | 0.002429 | -4.293 | 0.0000176 | 0.002869 |
| ENSG00000149927.DOC2A | -5.0655 | 0.000000407 | 0.0000991 | -4.21 | 0.0000255 | 0.002978 |
| ENSG00000047932.GOPC | -3.3568 | 0.000789 | 0.022603 | -4.1288 | 0.0000365 | 0.003471 |
| ENSG00000258429.PDF | 4.002 | 0.0000628 | 0.004588 | 4.051 | 0.000051 | 0.004476 |
| ENSG00000136270.TBRG4 | 3.416 | 0.000635 | 0.021575 | 4.033 | 0.0000551 | 0.004491 |
| ENSG00000232859.LYRM9 | -4.1376 | 0.0000351 | 0.002849 | -4.0123 | 0.0000601 | 0.004572 |
| ENSG00000108272.DHRS11 | -3.979 | 0.0000692 | 0.004675 | -3.869 | 0.000109 | 0.006786 |
| ENSG00000119242.CCDC92 | -4.0694 | 0.0000471 | 0.003622 | -3.8383 | 0.000124 | 0.007074 |
| ENSG00000145982.FARS2 | 3.655 | 0.000257 | 0.011387 | 3.8063 | 0.000141 | 0.007313 |
| ENSG00000142453.CARM1 | -3.562 | 0.000368 | 0.014149 | -3.659 | 0.000253 | 0.011547 |
| ENSG00000066629.EML1 | 3.0647 | 0.00218 | 0.045474 | 3.613 | 0.000303 | 0.012805 |
| ENSG00000174939.ASPHD1 | -3.9751 | 0.0000704 | 0.004675 | -3.589 | 0.000332 | 0.013529 |
| ENSG00000148842.CNNM2 | -3.81525 | 0.000136 | 0.008495 | -3.57976 | 0.000344 | 0.013535 |
| ENSG00000116353.MECR | 3.1124 | 0.001856 | 0.041085 | 3.42666 | 0.000611 | 0.021126 |
| ENSG00000133606.MKRN1 | 3.1434 | 0.00167 | 0.039998 | 3.415 | 0.000638 | 0.021411 |
| ENSG00000168575.SLC20A2 | -3.363 | 0.000771 | 0.022603 | -3.393 | 0.000691 | 0.021772 |
| ENSG00000140945.CDH13 | -3.6282 | 0.000285 | 0.011897 | -3.3404 | 0.000837 | 0.024634 |
| ENSG00000089041.P2RX7 | -3.7795 | 0.000157 | 0.008495 | -3.3134 | 0.000922 | 0.025048 |
| ENSG00000147419.CCDC25 | -5.1555 | 0.000000253 | 0.0000739 | -3.2107 | 0.001324 | 0.033571 |
| ENSG00000132612.VPS4A | 3.5771 | 0.000347 | 0.013702 | 3.1063 | 0.00189 | 0.04169 |
| ENSG00000040933.INPP4A | -3.542 | 0.000397 | 0.014872 | -3.042 | 0.00235 | 0.047574 |
| ENSG00000136874.STX17 | -3.3219 | 0.000894 | 0.024644 | -3.028 | 0.00246 | 0.047574 |
| ENSG00000115073.ACTR1B | 3.1049 | 0.001903 | 0.041497 | 3.033 | 0.00242 | 0.047574 |
| ENSG00000125458.NT5C | -7.7771 | 7.42E-15 | 5.42E-12 | - | - | - |
| ENSG00000148700.ADD3 | 6.964 | 3.31E-12 | 1.61E-09 | - | - | - |
| ENSG00000114904.NEK4 | 5.51525 | 3.48E-08 | 0.0000127 | - | - | - |
| ENSG00000182732.RGS6 | -4.8123 | 0.00000149 | 0.000301 | - | - | - |
| ENSG00000167193.CRK | 4.5167 | 0.00000628 | 0.001019 | - | - | - |
| ENSG00000129353.SLC44A2 | -4.445 | 0.0000088 | 0.001286 | - | - | - |
| ENSG00000104497.SNX16 | 4.3673 | 0.0000126 | 0.001674 | - | - | - |
| ENSG00000134900.TPP2 | 4.326 | 0.0000152 | 0.001851 | - | - | - |
| ENSG00000105928.DFNA5 | 4.2949 | 0.0000175 | 0.001967 | - | - | - |
| ENSG00000180979.LRRC57 | 4.2617 | 0.0000203 | 0.002118 | - | - | - |
| ENSG00000175115.PACS1 | 4.18092 | 0.000029 | 0.002492 | - | - | - |
| ENSG00000143776.CDC42BPA | -3.7981 | 0.000146 | 0.008495 | - | - | - |
| ENSG00000214114.MYCBP | -3.789 | 0.000151 | 0.008495 | - | - | - |
| ENSG00000136111.TBC1D4 | -3.779 | 0.000157 | 0.008495 | - | - | - |
| ENSG00000175471.MCTP1 | -3.766 | 0.000166 | 0.008662 | - | - | - |
| ENSG00000162384.C1orf123 | 3.697 | 0.000218 | 0.010983 | - | - | - |
| ENSG00000138430.OLA1 | 3.6871 | 0.000227 | 0.011055 | - | - | - |
| ENSG00000214113.LYRM4 | 3.655 | 0.000257 | 0.011387 | - | - | - |
| ENSG00000196850.PPTC7 | -3.6542 | 0.000258 | 0.011387 | - | - | - |
| ENSG00000136295.TTYH3 | 3.6471 | 0.000265 | 0.011387 | - | - | - |
| ENSG00000189077.TMEM120A | 3.6105 | 0.000306 | 0.012419 | - | - | - |
| ENSG00000100288.CHKB | 3.5084 | 0.000451 | 0.016463 | - | - | - |
| ENSG00000058866.DGKG | -3.50211 | 0.000462 | 0.016463 | - | - | - |
| ENSG00000138777.PPA2 | 3.43166 | 0.0006 | 0.020871 | - | - | - |
| ENSG00000105963.ADAP1 | -3.3955 | 0.000685 | 0.022067 | - | - | - |
| ENSG00000185418.TARSL2 | -3.394 | 0.000689 | 0.022067 | - | - | - |
| ENSG00000081087.OSTM1 | -3.387 | 0.000707 | 0.022067 | - | - | - |
| ENSG00000152291.TGOLN2 | -3.384 | 0.000714 | 0.022067 | - | - | - |
| ENSG00000166501.PRKCB | 3.38 | 0.000725 | 0.022067 | - | - | - |
| ENSG00000188690.UROS | -3.35751 | 0.000786 | 0.022603 | - | - | - |
| ENSG00000169609.C15orf40 | 3.3293 | 0.000871 | 0.024472 | - | - | - |
| ENSG00000179889.PDXDC1 | -3.304 | 0.000953 | 0.025784 | - | - | - |
| ENSG00000125454.SLC25A19 | -3.2923 | 0.000994 | 0.026404 | - | - | - |
| ENSG00000086205.FOLH1 | -3.25436 | 0.001136 | 0.029637 | - | - | - |
| ENSG00000165118.C9orf64 | 3.225 | 0.00126 | 0.032296 | - | - | - |
| ENSG00000183605.SFXN4 | -3.20763 | 0.00134 | 0.033754 | - | - | - |
| ENSG00000110315.RNF141 | 3.2005 | 0.001372 | 0.033974 | - | - | - |
| ENSG00000197157.SND1 | -3.1783 | 0.00148 | 0.036038 | - | - | - |
| ENSG00000078269.SYNJ2 | -3.133 | 0.00173 | 0.040767 | - | - | - |
| ENSG00000066084.DIP2B | -3.116 | 0.00183 | 0.041085 | - | - | - |
| ENSG00000105705.SUGP1 | 3.116 | 0.00183 | 0.041085 | - | - | - |
| ENSG00000007402.CACNA2D2 | -3.116 | 0.00183 | 0.041085 | - | - | - |
| ENSG00000100150.DEPDC5 | 3.086 | 0.00203 | 0.043615 | - | - | - |
| ENSG00000145494.NDUFS6 | 3.0672 | 0.00216 | 0.045474 | - | - | - |
| ENSG00000172817.CYP7B1 | -3.0558 | 0.00224 | 0.045474 | - | - | - |
| ENSG00000151693.ASAP2 | -3.0563 | 0.002241 | 0.045474 | - | - | - |
| ENSG00000141295.SCRN2 | 3.0307 | 0.00244 | 0.048174 | - | - | - |
| ENSG00000141985.SH3GL1 | -3.031 | 0.00244 | 0.048174 | - | - | - |
| ENSG00000254986.DPP3 | - | - | - | -6.3186 | 2.64E-10 | 0.000000151 |
| ENSG00000138688.KIAA1109 | - | - | - | -5.1246 | 0.000000298 | 0.000085 |
| ENSG00000163346.PBXIP1 | - | - | - | -4.61768 | 0.00000388 | 0.000877 |
| ENSG00000132613.MTSS1L | - | - | - | 4.582 | 0.00000461 | 0.000877 |
| ENSG00000123106.CCDC91 | - | - | - | 4.2072 | 0.0000259 | 0.002978 |
| ENSG00000161904.LEMD2 | - | - | - | 4.205 | 0.0000261 | 0.002978 |
| ENSG00000145198.VWA5B2 | - | - | - | -4.1728 | 0.0000301 | 0.003122 |
| ENSG00000112659.CUL9 | - | - | - | 3.8983 | 0.0000969 | 0.006786 |
| ENSG00000166140.ZFYVE19 | - | - | - | 3.8766 | 0.000106 | 0.006786 |
| ENSG00000110514.MADD | - | - | - | 3.8611 | 0.000113 | 0.006786 |
| ENSG00000130429.ARPC1B | - | - | - | 3.811 | 0.000138 | 0.007313 |
| ENSG00000120451.SNX19 | - | - | - | -3.7414 | 0.000183 | 0.009078 |
| ENSG00000138175.ARL3 | - | - | - | 3.68924 | 0.000225 | 0.010697 |
| ENSG00000196663.TECPR2 | - | - | - | 3.628 | 0.000286 | 0.012551 |
| ENSG00000136193.SCRN1 | - | - | - | -3.5626 | 0.000367 | 0.013958 |
| ENSG00000112667.DNPH1 | - | - | - | 3.5073 | 0.000453 | 0.016473 |
| ENSG00000111325.OGFOD2 | - | - | - | 3.502 | 0.000462 | 0.016473 |
| ENSG00000068971.PPP2R5B | - | - | - | -3.3892 | 0.000701 | 0.021772 |
| ENSG00000197150.ABCB8 | - | - | - | -3.3873 | 0.000706 | 0.021772 |
| ENSG00000182149.IST1 | - | - | - | -3.3386 | 0.000842 | 0.024634 |
| ENSG00000041353.RAB27B | - | - | - | 3.325 | 0.000883 | 0.025048 |
| ENSG00000163510.CWC22 | - | - | - | 3.314 | 0.00092 | 0.025048 |
| ENSG00000196236.XPNPEP3 | - | - | - | -3.269 | 0.00108 | 0.028658 |
| ENSG00000079739.PGM1 | - | - | - | -3.2584 | 0.00112 | 0.029044 |
| ENSG00000075975.MKRN2 | - | - | - | 3.2 | 0.00137 | 0.033982 |
| ENSG00000146085.MUT | - | - | - | -3.1902 | 0.00142 | 0.034473 |
| ENSG00000160285.LSS | - | - | - | 3.176 | 0.00149 | 0.034929 |
| ENSG00000143549.TPM3 | - | - | - | -3.17373 | 0.0015 | 0.034929 |
| ENSG00000154229.PRKCA | - | - | - | 3.1403 | 0.00169 | 0.038566 |
| ENSG00000065609.SNAP91 | - | - | - | 3.1048 | 0.0019 | 0.04169 |
| ENSG00000173821.RNF213 | - | - | - | -3.0706 | 0.00214 | 0.046071 |
| ENSG00000170445.HARS | - | - | - | 3.047 | 0.00231 | 0.047574 |
| ENSG00000181915.ADO | - | - | - | -3.04516 | 0.002326 | 0.047574 |
| ENSG00000173221.GLRX | - | - | - | -3.035 | 0.00241 | 0.047574 |

**Table S3 Genetic information and PWAS results for overlapping genes**

| ID | CHR | P0 | P1 | HSQ | BEST.GWAS.ID | BEST.GWAS.Z | pQTL.ID | pQTL.R2 | pQTL.Z | pQTL.GWAS.Z | NSNP | NWGT | MODEL | MODELCV.R2 | MODELCV.PV | PWAS.Z | PWAS.P | FDR |
| --- | --- | --- | --- | --- | --- | --- | --- | --- | --- | --- | --- | --- | --- | --- | --- | --- | --- | --- |
| ROSMAP | | | | | | | | | | | | | | | | | | |
| ENSG00000149927.DOC2A | 16 | 30016830 | 30034591 | 0.0447 | rs12921996 | -5.52 | rs11642612 | 3.38E-02 | 4.56 | -4.306 | 74 | 74 | blup | 0.038 | 9.10E-05 | -5.0655 | 4.07E-07 | 9.91E-05 |
| ENSG00000122574.WIPF3 | 7 | 29846102 | 29956682 | 0.0758 | rs10488082 | 6.03 | rs174917 | 0.047611 | -5.37 | -4.496 | 135 | 2 | lasso | 0.054 | 3.20E-06 | 4.7926 | 1.65E-06 | 0.000301331 |
| ENSG00000133706.LARS | 5 | 145492601 | 145562223 | 0.044 | rs2962540 | 5.12 | rs12188581 | 0.069583 | -5.65 | 4.212 | 87 | 1 | top1 | 0.07 | 1.30E-07 | -4.212 | 2.53E-05 | 0.002428912 |
| ENSG00000100266.PACSIN2 | 22 | 43231418 | 43411151 | 0.1045 | rs5751372 | 6.08 | rs17415203 | 0.031901 | 5.57 | 1.036 | 163 | 18 | enet | 0.087 | 3.30E-09 | 4.2004 | 2.66E-05 | 0.002428912 |
| ENSG00000232859.LYRM9 | 17 | 26205340 | 26221778 | 0.1469 | rs4796190 | 4.25 | rs7218395 | 2.62E-01 | -10.27 | 4.145 | 75 | 3 | lasso | 0.29 | 2.60E-29 | -4.1376 | 3.51E-05 | 0.00284895 |
| ENSG00000119242.CCDC92 | 12 | 124403207 | 124457378 | 0.0507 | rs10846577 | 4.43 | rs12309481 | 0.058099 | -5.61 | 3.715 | 83 | 83 | blup | 0.075 | 3.90E-08 | -4.0694 | 4.71E-05 | 0.003621742 |
| ENSG00000258429.PDF | 16 | 69362524 | 69364498 | 0.078 | rs9939870 | 5.3 | rs877534 | 7.26E-02 | 5.68 | 4.002 | 65 | 1 | top1 | 0.073 | 6.70E-08 | 4.002 | 6.28E-05 | 0.00458754 |
| ENSG00000108272.DHRS11 | 17 | 34948228 | 34957235 | 0.1559 | rs11650008 | -5.24 | rs35712149 | 2.80E-01 | -10.45 | 3.979 | 101 | 1 | top1 | 0.28 | 1.00E-28 | -3.979 | 6.92E-05 | 0.0046752 |
| Banner | | | | | | | | | | | | | | | | | | |
| ENSG00000149927.DOC2A | 16 | 30016830 | 30034591 | 0.0664 | rs12921996 | -5.52 | rs35105141 | 0.053619 | 4.16 | -4.21 | 75 | 1 | top1 | 0.054 | 0.0024 | -4.21 | 2.55E-05 | 0.00297801 |
| ENSG00000122574.WIPF3 | 7 | 29846102 | 29956682 | 0.2121 | rs10488082 | 6.03 | rs17606308 | 0.10787 | -5.15 | -5.052 | 114 | 114 | blup | 0.13 | 3.50E-06 | 7.3733 | 1.66E-13 | 1.89E-10 |
| ENSG00000133706.LARS | 5 | 145492601 | 145562223 | 0.0981 | rs2962540 | 5.12 | rs13180255 | 0.08211 | -4.1 | 3.959 | 87 | 32 | enet | 0.086 | 0.00014 | -4.293 | 1.76E-05 | 0.0028688 |
| ENSG00000100266.PACSIN2 | 22 | 43231418 | 43411151 | 0.1145 | rs5751372 | 6.078 | rs2899365 | 0.12228 | 5.42 | 5.038 | 140 | 14 | enet | 0.13 | 2.20E-06 | 5.709 | 1.14E-08 | 4.34E-06 |
| ENSG00000232859.LYRM9 | 17 | 26205340 | 26221778 | 0.242 | rs4796190 | 4.245 | rs3751972 | 0.268594 | -7.25 | 3.847 | 69 | 3 | lasso | 0.32 | 2.40E-14 | -4.0123 | 6.01E-05 | 0.004571607 |
| ENSG00000119242.CCDC92 | 12 | 124403207 | 124457378 | 0.45 | rs10846577 | 4.43 | rs12311114 | 0.653431 | -10.02 | 3.764 | 79 | 7 | lasso | 0.66 | 4.80E-37 | -3.8383 | 1.24E-04 | 0.0070742 |
| ENSG00000258429.PDF | 16 | 69362524 | 69364498 | 0.0708 | rs9939870 | 5.3 | rs1127231 | 0.048438 | 3.72 | 4.051 | 61 | 1 | top1 | 0.048 | 0.0037 | 4.051 | 5.10E-05 | 0.004476231 |
| ENSG00000108272.DHRS11 | 17 | 34948228 | 34957235 | 0.2353 | rs11650008 | -5.241 | rs17619821 | 0.242179 | -6.5 | 3.869 | 87 | 1 | top1 | 0.24 | 7.20E-11 | -3.869 | 1.09E-04 | 0.006785947 |

**Table S4 Detailed information of colocalization analysis between variants and overlapping genes**

| ID | COLOC.PP0 | COLOC.PP1 (H_1_) | COLOC.PP2 (H_2_) | COLOC.PP3 (H_3_) | COLOC.PP4 (H_4_) |
| --- | --- | --- | --- | --- | --- |
| ENSG00000149927.DOC2A | 0.001 | 0.003 | 0.014 | 0.055 | 0.927 |
| ENSG00000100266.PACSIN2 | 0 | 0.002 | 0 | 0.128 | 0.87 |
| ENSG00000133706.LARS | 0.005 | 0.243 | 0.002 | 0.081 | 0.67 |
| ENSG00000119242.CCDC92 | 0.001 | 0.412 | 0 | 0.019 | 0.568 |
| ENSG00000232859.LYRM9 | 0 | 0.477 | 0 | 0.005 | 0.518 |
| ENSG00000108272.DHRS11 | 0 | 0.354 | 0 | 0.27 | 0.376 |
| ENSG00000258429.PDF | 0.004 | 0.259 | 0.007 | 0.428 | 0.301 |
| ENSG00000122574.WIPF3 | 0.001 | 0.018 | 0.037 | 0.735 | 0.21 |

**Table S5 Genetic information and SMR results for overlapping genes**

| probeID | ProbeChr | Gene | Probe_bp | topSNP | topSNP_chr | topSNP_bp | A1 | A2 | Freq | b_GWAS | se_GWAS | p_GWAS | b_pQTL | se_pQTL | p_pQTL | b_SMR | se_SMR | p_SMR | p_HEIDI | nsnp_HEIDI |
| --- | --- | --- | --- | --- | --- | --- | --- | --- | --- | --- | --- | --- | --- | --- | --- | --- | --- | --- | --- | --- |
| ROSMAP | | | | | | | | | | | | | | | | | | | | |
| Q53HC0 | 12 | *CCDC92* | 124528518 | rs12309481 | 12 | 124389150 | A | C | 0.379346 | 0.0313049 | 0.0082 | 1.206000e-04 | -0.02941 | 0.0051341 | 1.013999e-08 | -1.06443 | 0.335062 | 1.489034e-03 | 9.724044e-01 | 13 |
| Q6UWP2 | 17 | *DHRS11* | 35057217 | rs35712149 | 17 | 34960336 | T | G | 0.388548 | 0.0289964 | 0.0079 | 2.213000e-04 | -0.087 | 0.00767263 | 8.409059e-30 | -0.333292 | 0.0954434 | 4.793518e-04 | 8.110566e-02 | 15 |
| Q14183 | 16 | *DOC2A* | 30119525 | rs11642612 | 16 | 30030195 | C | A | 0.383436 | -0.0320024 | 0.0076 | 2.444000e-05 | 0.02928 | 0.00636041 | 4.155000e-06 | -1.09298 | 0.351772 | 1.889536e-03 | 1.108817e-01 | 7 |
| Q9P2J5 | 5 | *LARS* | 145653104 | rs12188581 | 5 | 145483359 | A | G | 0.250511 | 0.0361973 | 0.0083 | 1.389000e-05 | -0.03024 | 0.00526377 | 9.196001e-09 | -1.197 | 0.344597 | 5.134831e-04 | 8.221796e-01 | 5 |
| A8MSI8 | 17 | *LYRM9* | 26316814 | rs7218395 | 17 | 26241477 | G | A | 0.304703 | 0.0295005 | 0.0079 | 2.077000e-04 | -0.1642 | 0.0148168 | 1.534013e-28 | -0.179662 | 0.0507701 | 4.020448e-04 | 1.354629e-01 | 13 |
| Q9UNF0 | 22 | *PACSIN2* | 43510219 | rs17415203 | 22 | 43455592 | A | G | 0.108384 | 0.00790113 | 0.0114 | 4.886000e-01 | 0.04743 | 0.00841562 | 1.740998e-08 | 0.166585 | 0.242165 | 4.915160e-01 | 2.278100e-03 | 14 |
| Q9HBH1 | 16 | *PDF* | 69461367 | rs877534 | 16 | 69367996 | A | C | 0.281186 | 0.0338981 | 0.008 | 2.088000e-05 | 0.05684 | 0.00969962 | 4.627997e-09 | 0.596378 | 0.173686 | 5.954836e-04 | 1.915993e-01 | 8 |
| A6NGB9 | 7 | *WIPF3* | 30045414 | rs174917 | 7 | 29849897 | A | G | 0.097137 | -0.053 | 0.0122 | 1.435000e-05 | -0.09771 | 0.0179055 | 4.843003e-08 | 0.542421 | 0.159594 | 6.769029e-04 | 1.726455e-01 | 4 |
| Banner | | | | | | | | | | | | | | | | | | | | |
| Q53HC0 | 12 | *CCDC92* | 124549174 | rs2178663 | 12 | 124433905 | C | T | 0.652352 | -0.0296039 | 0.0079 | 1.870000e-04 | -0.134462 | 0.010841 | 2.512927e-35 | 0.220166 | 0.0613757 | 3.342698e-04 | 5.214072e-01 | 20 |
| Q6UWP2 | 17 | *DHRS11* | 35057217 | rs12936319 | 17 | 34959459 | A | G | 0.611452 | -0.028595 | 0.0078 | 2.620000e-04 | -0.07706 | 0.0116615 | 3.893335e-11 | 0.371074 | 0.115753 | 1.347161e-03 | 1.905392e-01 | 10 |
| Q14183 | 16 | *DOC2A* | 30119525 | rs11150581 | 16 | 30030699 | T | C | 0.616564 | 0.0321961 | 0.0076 | 2.212000e-05 | 0.0371306 | 0.00640762 | 6.842338e-09 | 0.867105 | 0.253547 | 6.264529e-04 | 1.203681e-02 | 8 |
| Q9P2J5 | 5 | *LARS* | - | - | - | - | - | - | - | - | - | - | - | - | - | - | - | - | - | - |
| A8MSI8 | 17 | *LYRM9* | - | - | - | - | - | - | - | - | - | - | - | - | - | - | - | - | - | - |
| Q9UNF0 | 22 | *PACSIN2* | 43510219 | rs5759012 | 22 | 43286876 | A | G | 0.538855 | -0.0372035 | 0.0074 | 5.442000e-07 | 0.0344882 | 0.00583188 | 3.344489e-09 | -1.07873 | 0.281625 | 1.279432e-04 | 5.379484e-01 | 15 |
| Q9HBH1 | 16 | *PDF* | - | - | - | - | - | - | - | - | - | - | - | - | - | - | - | - | - | - |
| A6NGB9 | 7 | *WIPF3* | 30045414 | rs174916 | 7 | 29849570 | A | G | 0.0746421 | -0.0593042 | 0.0131 | 5.918000e-06 | 0.0887772 | 0.016254 | 4.711068e-08 | -0.668012 | 0.191657 | 4.913028e-04 | 5.483668e-01 | 7 |

**Table S6 Transcriptional data sources and results for differential expression analysis**

| PMID | Species | Tissue types | Sample size of donors | Sample size of cultures | Experiment type | Gene | P-value | t | df | Estimate | Std. Error | Statistical Method |
| --- | --- | --- | --- | --- | --- | --- | --- | --- | --- | --- | --- | --- |
| 33398088. | Homo sapiens | Neuron | N_BD_ = 6 N_control_ = 8 | N_BD_ = 36, N_control_ = 52 | Expression profiling by high throughput sequencing | *DOC2A* | 4.26E-02 | - | - | -0.253 | 0.111 | LMM |
|  |  |  |  |  |  | *STXBP1* | 8.00E-03 | - | - | -0.178 | 0.062 | LMM |
|  |  |  |  |  |  | *UNC13A* | 7.02E-03 | - | - | -0.275 | 0.085 | LMM with 1000 parametric bootstrap simulations |
| 33667413 | Homo sapiens | Astrocyte | N_BD_ = 5, N_control_ = 4 | N_BD_ = 19, N_control_ = 14 | Expression profiling by high throughput sequencing | *DOC2A* | 2.09E-02 | -3.83 | 31 | -0.024 | 0.008 | LMM |
| 31123247 | Homo sapiens | Hippocampus | N_BD_ = 18, N_control_ = 18 | - | Expression profiling by array | *DOC2A* | 9.80E-03 | -2.74 | 34 | - | - | Student’s t-test |
|  |  | Pre-frontal cortex | N_BD_ = 17, N_control_ = 19 | - |  |  | 1.44E-02 | -2.58 | 34 | - | - | Student’s t-test |
| 19255580 | Homo sapiens | Pre-frontal cortex | N_SCZ_ = 28, N_control_ = 23 | - | Expression profiling by array | *DOC2A* | 1.49E-01 | -1.47 | 49 | - | - | Student’s t-test |
| 21614001 | Homo sapiens | Frontal cortex | N_ASD_ = 16, N_control_ = 16 | - | Expression profiling by array | *DOC2A* | 4.38E-01 | -0.79 | 30 | - | - | Student’s t-test |

**Table S7 Co-expressed genes of DOC2A identified by WGCNA**

| Neuron dataset- turquoise module | Astrocyte dataset- red module |
| --- | --- |
| *PTF1A/SNORD115-45/MIR6806/SNORD12C/RNU6-353P/ATP10B/NPNT/RNU6-137P/RASGRF1/MT3/GATA3/MIR2116/LINC01322/LINC00645/HOXB-AS1/SLC6A17/RFPL2/PRSS56/SLFN12/RIT2/RNU7-75P/SLCO1C1/SLC7A14/CHRNA6/DSCAM/RNU4-47P/SLC4A10/MATK/WIF1/CHD5/LINC00928/PLPPR4/MRPL9P1/GABRB1/PTPRR/SCGN/GABRA2/PRPH/CA10/SCARNA18/SNORD1B/SNORA63C/SNORD51/MIR181D/GRIN3A/PCDHB5/SNORA15/CSMD1/FOLH1/GRM1/ARFGEF3/PCP4L1/ZPLD1/LPAR6/LINC02520/ZNF471/SCARNA21/SCN2A/RNF43/KCNJ3/PPP2R2C/DAPL1/KSR2/RAB27B/CHL1-AS2/IRX4/ADRA1A/TNFRSF1B/ARPP21/SKOR1/JAKMIP1/ONECUT3/GPRIN3/ABLIM3/PCDHGB4/APELA/HPD/GOLGA7B/LRP1B/RASD2/PPP1R1B/GRIN1/SLITRK6/CHRM2/SERTM1/DCT/C22orf42/MAB21L1/CNTNAP4/FSTL4/MC4R/FOXB1/GRM7/TMEM229B/OR5AH1P/GLRA3/KCNK9/NPIPB15/FOXA1/LINC00643/ICAM5/CHRNB3/GABRG3/CACNG5/NR4A2/ESPN/APBA1/VWA5B2/STUM/SYT16/HS6ST3/SPOCK3/SNCB/CCDC198/KLHL1/THSD7B/CDH9/KCNC2/CNTN5/ATP2B3/B3GALT2/LINC02488/MIR1302-3/PDIA2/KCNA4/ACSL6/ZNF804A/MIR4787/SAMD3/HES2/RN7SL417P/KCND2/HOXB2/DUSP5P1/SLC17A8/EPHA10/GCK/PAK6/PACSIN1/TRIM7/SLITRK3/PLA2G3/RNU6-1099P/LY6H/SLC7A2/MIR4489/RBFOX1/LRRC3B/CDKL2/ENTPD2/CD8A/IFI44L/BRINP2/COX6CP13/RHOXF1/SLC24A2/VSNL1/TYRP1/GRM5/TFAP2B/ABI3BP/RYR2/C20orf203/CYP1B1/SHOX2/CNTN3/ITIH5/RLBP1/DDN/GABRG2/KCTD4/ADCYAP1/DISC1FP1/FST/CDH12/SLC35F4/PLXNA4/CBLN1/COL8A2/NDUFA3P4/TNR/TRANK1/CD36/B3GAT2/NYAP2/CNTNAP5/FSTL5/SFRP4/LUZP2/DOC2B/GPR83/LINC02223/ENPEP/EPHA5/ALOXE3/LRRC7/PCSK9/PLCXD3/PLXDC1/BRINP3/CACNA1I/CREG2/FAM78B/B3GNT9/HLA-DMA/GAL/NTS/CECR7/NALCN/TRH/MGAT4C/CCDC144NL/RN7SL585P/OPRM1/RMDN2-AS1/GPR39/NEXN/LINGO2/GALNT14/LGI3/ZIM2-AS1/LINC01833/PLXNB3/MIR3936/UNC13C/MIRLET7F1/LMO1/DNAJC22/KCNH3/RPSAP44/DMBX1/GPC5/COX6B2/CYP1A1/NID1/LDHD/SNRPFP1/DPY19L2P4/HAPLN2/PRRX2/IL10RA/APOC1/FAM19A1/NTSR1/PDZRN4/GPR45/ATP2B2/CTXN3/VAX2/NRG2/ASIC4/AKAP14/SAMD5/LHFPL3/RNY3P8/BCL11B/HCRTR2/GABRA3/HTRA3/CHRM4/RGS11/RETREG1/ENPP5/LRRC3-AS1/FBXL8/CAMK2B/IRF5/STRA6/MMD2/IGFBP6/RDH12/GALNT9/KCNS2/KCNQ3/KCTD8/FOSL1/ESYT3/LINC01586/PAPPA2/MIRLET7I/TCEAL2/NEUROG2/TRIM47/GRM4/LRFN5/NOVA1-AS1/FAM46B/SCML2P2/SYCE3/ZNF835/MMP10/ZSCAN1/P2RX7/GLRA2/LNX1/PCDH15/SNORA73B/THSD4-AS1/EEF1DP4/CACNG2/FAM163B/C1orf61/TMEM266/ITGA1/SYNPR/CALB2/APLN/AMER3/SLC18A3/CACNA1E/CACNA2D3/EPAS1/C8orf34/GPR61/GPR12/ADCY8/LINC00565/ADGRA1-AS1/MT1F/SNORA2B/SORCS3/NXPH3/CSMD3/PABPC1L2A/VN1R110P/SSUH2/TRADD/EPHA6/STXBP5-AS1/LINC02387/SLC17A7/DNM3/4-Mar/HMGN2P30/HSF4/RIMS1/GABRA5/RBP4/NCAM2/SCN9A/CACNA1D/PPFIA4/C6orf141/KCNJ9/NXPH4/SNORD14E/SSTR1/NPY5R/CALN1/LRRC36/SEMA3B-AS1/ZNF571-AS1/CHRM3/ARHGEF28/PROCR/RASGEF1A/KDR/CSMD2/UNC5A/CCNA1/SV2C/LINC02195/ADAM29/TPTE2P1/NEGR1/CAPN14/GRID2/LINC02356/LINC01801/PDE4C/MMP24/NR3C2/OCSTAMP/HS3ST5/OPLAH/GABBR2/SCN2B/ATP2B1-AS1/DIO2/ULBP2/SHC4/ZNF385B/ZNF726/LINC01122/PLK5/MCF2L2/RN7SL688P/CKS1BP3/DCTN1-AS1/CRISPLD2/TMEM220-AS1/CRYM/PRR16/ANKRD24/MORN5/IQSEC3/CNTN1/PRSS12/PDK4/RUNDC3B/WFIKKN2/GPR158/GRIK1/RGS6/GLI1/PLPPR1/GRB14/SLC17A6/ALPK1/ESRRG/KIAA0319/MIR137HG/LINC02200/GBGT1/SIAH3/SPHKAP/SNORA79B/DIRC3/CLEC2L/REPS2/GRIA2/ACE/ANKRD1/SEPT12/MAPT/GATA3-AS1/SLC24A3/SNORD20/LURAP1L-AS1/TMEM151A/TH/LINC00652/CYYR1/GCKR/HIST1H3F/CLDN5/RGS7/HAS1/IRX6/SOWAHA/LAMA2/ELFN2/SNORA80D/GAD1/STOM/LINC02487/CHODL/RPL12P30/PRCD/TFR2/MLN/CNGB1/CRLF1/RPS15AP17/CDH5/PRDM1/APOBEC3B/CCDC85A/LINC00884/RPL39P40/MIR7-3HG/CCDC92B/LINC00957/MIR4322/LRFN2/CCDC169/ATP5PBP5/IL13RA2/RANP2/LINC02134/RERG/PCDHGB1/SNRPGP9/POU6F2/PPFIA2/SGCZ/CDH22/PDE11A/PLCL1/SCRT2/SPTBN4/VIPR1/TNFRSF10D/LYN/MAOA/SCML4/GRIK3/MYT1L/PRKCH/PAK5/LMX1B/WFDC21P/NRN1/C19orf66/STEAP1/FOXC1/TCTEX1D1/BEX5/NDUFA4L2/MIR6807/CPEB1/NGEF/FNDC9/TPD52L1/COL13A1/FGF11/FBLN5/PSCA/CBLN2/HPCAL4/RTP1/GALNTL6/ENTPD3/PENK/FAM196B/SNORD17/SNORA80E/PEG13/FGF14/ROPN1L/ADAP1/THAP5P1/CPEB3/ISLR2/EIF3KP1/CNTNAP2/MISP/NGB/TMEM37/ZFYVE28/DIRAS2/ZNF365/MIR4500HG/RAB6C-AS1/SLC26A1/KCNB2/BDNF/BRD9P2/DGCR10/CHSY3/SLC12A5/PPP1R16B/LDLRAD4/SNHG26/AHNAK2/HMGN1P17/RPL10P19/CLEC3A/ADAMTS1/CCNI2/CDH18/MAOB/LINC01821/GHET1/MPZL3/P2RX5/CHL1/KRT18P48/RIN3/RBFOX3/SNORA71A/RNA5SP508/GABRA1/CPNE7/HECW1/STAMBPL1/GRIP2/TSPOAP1-AS1/DYNC1I1/SCARNA8/CABCOCO1/NPY1R/LINC01515/VSTM2A/LAMB3/HIST1H2BO/TNFRSF10A/LYSMD2/GLB1L3/LINC02556/CALY/RGS10/CACNA1C/HHIPL1/FAM155A/SNAI1/DUSP8/HLF/SLC46A3/PSD2/HS3ST4/FSCN2/CASC9/JAKMIP3/FGF19/KCNK3/CAVIN1/PLP2/NPM2/LMO2/FGFBP1/LINC01668/RBMS3-AS3/SERPINB1/HNRNPA1P1/PCSK1/GRIN2A/RXRG/KCNIP4/HIST1H4D/FAM181B/RPSAP52/SLC6A1/MYOM1/HMGN2P27/VN1R20P/GRID1/CGREF1/KRT8P30/IDUA/CERNA1/VSTM2A-OT1/CDK5R2/GFOD1/MCF2L-AS1/RTL1/MAB21L2/RAB20/TTC29/FOXO6/DOCK2/TAC1/ZBTB7C/UBE2QL1/OCA2/DOK6/SLC13A5/TFAMP1/POLR2F/HPX/KCNS3/CSGALNACT1/LAMC2/ATP8A2/GPR85/CCNL2P1/RUNDC3A-AS1/MID2/RNU6-703P/XKR4/PRKCQ-AS1/BAALC-AS2/HPCA/LINC01844/LRRC26/GPR155/FAM71F1/UNC79/LINC01976/F12/VN1R108P/ENPP3/MESP1/C4orf50/ABCG4/CORO2A/CCSER1/HIST1H2BE/TTLL9/DNAH2/TMEM26/TMEM191A/PLA2G4C/HAR1A/FBXO27/KIAA1024L/SQOR/IL4R/ECHDC2/EWSAT1/RFPL1S/GUCY1A1/CCNB3/RARRES2P1/PCDHA1/ST8SIA3/ARHGEF26-AS1/PCDH10/RASGEF1B/ALKAL2/C17orf50/CHDH/TYMP/RUNX1T1/ABCB1/LINC02238/SYTL5/DDC/NTNG1/MEF2C/NOL4/GRIK2/TMIE/CYP4X1/THSD4/RALYL/COX7A2P1/KCNK15-AS1/LINC01546/GLTPD2/RAB3IL1/HRK/PPIAP67/SLC41A2/NID2/TSPAN1/C2orf27A/ZMYND12/SLC1A1/MSANTD1/PCDHA12/PTPRH/CELF4/SHISA8/SLC9A9/RGS9/SLC8A3/B3GNT4/DRC1/OCM/SNORD101/LINC02361/BASP1-AS1/ISM1/PPARGC1A/LINC01220/SCX/TFP1/NPHS1/MIR7111/BMPER/SNX18P7/ARMC4/TSHR/SSTR2/CCL25/PLCB2/TDRD12/GABRQ/MAP10/FRRS1L/LANCL3/DHDH/PLCH2/BEST1/CHGA/ARHGAP20/SYTL3/URAHP/F3/KRT222/MGARP/VWC2L/CATSPERG/SPATA6L/CACNB2/SNORD12B/TNFAIP8L3/COL20A1/HMGN2P25/IPCEF1/EPCAM/MTUS2/ROR2/BSN-AS2/ITPRIPL2/SPRY4-AS1/AJM1/EIF1P6/PTPN3/SNRPFP2/MAP7D2/TAPBP/MPZ/NDST3/GRM8/FAM228A/OMG/KCNA2/ANK1/DACT3-AS1/C2orf16/HK3/SGSM1/PRRT4/PTPRE/GHRHR/NABP1/SLC30A4/HGFAC/CES4A/PLAGL1/FXYD2/CYGB/LINC01102/GALNT5/RAI2/KCNH5/SYT13/ERAS/WIPF3/FRRS1/GTF2H2/HPGD/RTN4RL1/NKAIN2/C1orf232/CLSTN2/TM6SF2/NAV3/CACNA1A/CKMT1B/SERPINI1/HMGCLL1/PRSS53/XKR8/RN7SL181P/CLVS2/USP12-AS2/SMIM22/ELN/TESC/RNU6-45P/FAAH2/BCRP8/SLC22A20P/DPH6-AS1/PCDHB6/FAM212A/PPIAP4/SAMD12/NKAIN3/BHLHE41/TRMT112P3/LINC00488/OTOL1/CYB5R2/LRRTM3/FOXD4/SUSD4/TF/C5AR1/KLHL32/IGFL4/LINC01864/P3H2/LRRTM1/RHOV/HERC5/SPART-AS1/SYT3/DYNLRB2/HIVEP3/ZNF843/KCNMB2-AS1/SLC5A4-AS1/ELMOD1/CYP1B1-AS1/RAB9B/OPRL1/NAT16/CKMT1A/CAPS2/HS3ST1/LINC02298/VWC2/RASSF5/HR/ADAP2/DNAJC6/PDE8B/C1QL4/TPH2/LINC00304/BEAN1/EPB41L4B/RAP1GAP2/ZNF30-AS1/SGIP1/RIMBP2/RASA4/RPL23AP80/DYNLT3P2/NIM1K/HAPLN3/IL12A/SCGB2B2/SERTAD4-AS1/SOGA3/FAM19A2/PCDHA2/ODAPH/HCRTR1/LINC-PINT/NRXN1/GLUD1P2/FAM66D/HIST1H3B/RTL9/DUSP26/PABPC5-AS1/AJAP1/PPIC/INHBB/STYK1/SHBG/PDZD7/CYP2E1/NWD2/SLC30A10/CCDC33/NRSN1/TINCR/APOA1/LINC01771/ANGPT2/PATJ/UNC5D/MAP3K5/PTCH2/RELN/RNF112/HMGN2P28/RPS12P31/SHISA7/JHY/XK/HSPE1P5/SLITRK1/STK32A/PCDHB9/HIST1H2BL/ITGA10/C3orf52/BRINP1/RGS8/HIST1H2AL/NR1I3/TBC1D30/CA3/DGCR9/LINC02427/DBH/F11R/FAM241A/HIVEP2/CHST9/CREB3L1/DACH2/STEAP1B/C2orf73/UPK2/SNHG28/ARHGDIG/TRIM6/GRIN2D/MFSD4A/GPR68/MEF2C-AS2/SYNJ2/RASGRP2/DNAJC27-AS1/PACRG/TMEM272/DRP2/PNMA6A/ENKUR/PNRC2P1/MRVI1-AS1/FMNL1/UTS2B/S100A1/UTS2/SOX14/SAMD11/NPM1P40/GRIA3/LEFTY1/PLSCR1/NOXA1/THEM5/SH3BP5/STX17-AS1/CTSS/SUGT1P3/LHFPL4/IGSF22/ARSE/DPPA2/ID2-AS1/SNTG1/TLE6/LGALS4/DNM1P51/MESTIT1/TDRD7/ACHE/HCN2/PTMAP8/CCK/C20orf204/SALL1/CLDN1/GGN/GNG8/LHFPL5/NTNG2/PCDHB16/MST1R/LMOD1/CABYR/SOX9-AS1/NFKBIZ/PAQR9-AS1/EML6/HIF1A-AS1/PLBD1/PAICSP1/RPS11P6/DISP2/TMEM200A/NANOS3/CMAHP/EIF1AXP1/SLC45A2/ADGRA1/WDR11-AS1/NLRX1/C5orf49/MT-TT/SLC44A3-AS1/RHCG/MOAP1/TRIM67/SNAP91/FAIM2/CDH3/HYI-AS1/TMOD2/SCGB1B2P/LINC00452/ORAOV1P1/OXCT1-AS1/RPRM/LYNX1/MMP1/LINC00368/MYO15A/SPINT1-AS1/GOLT1A/ERICH5/HMGN2P15/CHST1/PLA2G1B/TBC1D2/SRCIN1/SYN1/TMEM139/PABPC1L2B/LGSN/DMTN/TG/CFAP61/P3H2-AS1/ABCC9/CAMK2N2/SMTNL1/TTC9B/TMCC3/SCG2/LINGO1-AS1/LINC02552/ZFHX4-AS1/FUT2/LINC01978/HTR2A/CTSLP2/C3orf70/LINC01664/PRELID2/PIGZ/ZMYND10/TNNI3/ECEL1/F8A1/PID1/NRG3/HOMER3-AS1/DUX4L50/ARHGEF7-AS1/HMGN1P37/C1RL-AS1/GDA/ADCY1/SEZ6L/MYORG/NKG7/LRRC43/PTPRO/SCN3B/FAM13C/C4orf45/RN7SL388P/NEURL1/C2orf50/SCRT1/H1FX-AS1/SHANK1/BNIP3P1/DERL3/TFAP2E/POU5F1B/SNORA55/SLC8A2/AFF2/SLC44A3/HIST2H2BF/LINC01013/SYT5/OBSCN-AS1/DLGAP3/WFDC3/CABP1/KCNG2/CPSF1P1/TNIP3/PTPN5/RPS15AP36/RAMP2-AS1/DUSP10/IRAK2/AGBL4/ADAMTSL1/HNRNPA1P67/ADGRB3/GNAS-AS1/AK5/TAS1R3/NDST4/FAM189A1/TMEM179/ATP8A1/OR6E1P/KCNH1-IT1/TUBB2BP1/DSCR9/BICDL1/HMSD/C1QL1/FTH1P19/SNRPEP2/NTM/TMEM196/SLC8A1/C8G/LINC00910/NPM1P6/HIST1H4E/PCDHB12/SNAI1P1/DGKI/GUCA1B/ABALON/SHANK2/TTC36/SPATA4/CHST8/CHRM5/MYLK2/RIMKLA/SLC9A7/SCN3A/ITIH3/CRB1/PPP1R42/MUC12/DQX1/CARMIL2/RCAN2/MIR193BHG/PHYHIP/ATXN8OS/TACR2/IZUMO1/MYO15B/EXOC3L1/MASP1/PCAT1/LINC01881/SHB/PNMA8B/SEPT4/CDH13/TMEM225B/EPHA5-AS1/TEX45/CYP2W1/XKR7/COMTD1/NELL1/OSTF1/APOE/TTC9/HIST1H2AH/CBR3-AS1/ZNF488/LMTK3/PPIAP51/NEK11/ANKFN1/CACNA1B/GPR156/TGFA/SNORD3A/KCNIP1/CDIPT-AS1/HAUS7/BCL11A/IQSEC2/LINC01621/ADGRG3/GRIN2C/MITF/PARVB/KLHL2P1/MIR25/DAO/PFN4/DNAAF1/RASGRF2/SLC22A18/TMEM74B/SPOCK2/EXTL1/DPF3/CBFA2T3/LINC01166/ADAM11/B4GALT1-AS1/CMTM8/PXDNL/ACRBP/AGBL5-AS1/TRPC6/KDELC2/CHGB/IFNWP19/SSH3/TMEM255B/PRKAR2A-AS1/PTPRN/FAM66A/FPGT/SSSCA1-AS1/ENOX1/KIRREL3/DCLK3/SH3RF2/HMGB3P22/SH3GL3/SMIM10L2B/SLCO5A1/RLN1/TMEM102/CYP27C1/SYNGR3/COLEC11/VAV3/FBXO15/PCDHA11/SH3GL2/GZMM/HIST1H4C/LAMA3/IL2RB/FAM212B-AS1/FBXO41/GLS2/EPX/SHISAL2A/NMRK1/LRRC73/METTL11B/LINC01977/SPINT2/FZD9/SETD6/LINC01424/SEMA4A/PKIA-AS1/FAR2P2/MYH7/ISCA2P1/FTH1P25/ITFG2-AS1/DPP10-AS1/SUMO1P3/JPH3/C1orf229/AFP/ARHGEF4/SBK3/IL34/DUSP18/EGLN3/TOB1-AS1/PDE4A/RPL5P30/DEPP1/AFAP1L2/GLRB/SERTAD4/FAM129C/TNK1/MIR4737/DLK2/VWCE/NFATC1/CFAP100/MTMR7/CBS/C8orf89/TTBK1/SMN2/EPS8L2/RBPMS/ARHGAP19-SLIT1/CNR1/PCDHB7/MORN3/PPEF1/SLC45A1/NDUFA6-AS1/SH2D7/ADCY5/FZD2/CBX3P4/MYLK/TNNI3K/FAM161B/MPP7/PLEKHN1/BEGAIN/LINC02193/BPIFB9P/LINC00578/LINC00622/HMGA1P1/CFAP53/PGAM1P5/KNCN/RBP5/MAP3K14-AS1/PNMA5/PRRT3-AS1/FRY/TMEFF2/CFAP97D2/SSC5D/CCDC13-AS1/PTTG2/FHIT/ZNF157/PPIAP54/DCLRE1CP1/SNPH/WAS/DUX4L26/SLC16A14/LRRC4C/HIST1H2BC/MYADML2/NPTXR/EFNA1/TCAF2/CAPN3/MAP1LC3B2/TMEM232/SH2D6/C1QTNF4/CYP2T1P/PDCD6IPP2/VWA8-AS1/FGF18/ZMIZ1-AS1/LOH12CR2/CFP/LINC02449/GEMIN7-AS1/LINC00449/SNCA/MAGEL2/SLC1A2/SMIM18/PTENP1/CCNE1/TRAM1L1/SPATA1/SVOP/CFHR1/FRMPD4/PPIL6/GDF9/TRIM69/TCP10L/CNGA3/PDZD9/KLHL29/TMEM108/SUGT1P1/PRRT1/STOML1/SNAP25-AS1/HIST3H2BB/PNCK/RPSAP14/CES3/DRAIC/PCOLCE-AS1/NPM1P21/IQCJ-SCHIP1/FBN2/TCTE1/TSSK1A/ZNF836/PLA2G16/EIF4E1B/CEACAM19/NIPAL3/ROBO2/SPON2/H2AFZP3/ZC3H12A/HIST1H3J/DOCK3/HERC3/SMKR1/SMIM10L2A/FBXO2/LINC02518/SCARNA12/BAG2/IRX1/MYO16/F7/PNMA8C/BMP8A/NKILA/PHKA1/ROM1/DNASE2B/DCLK1/TMC3-AS1/SMAGP/MYOZ3/RRAD/LRRTM4/PRKN/ELOVL2-AS1/RAET1G/RNFT1P3/LINC02056/AOC2/CYP4F26P/LINC00960/HSPA12A/HMGB1P31/MEGF11/ACKR1/ARHGAP44/JAZF1/TLCD1/TMEM243/PRICKLE2/ANKRD34A/ANKS1B/KIF26A/LINC00337/IGSF1/MATN3/HTR1E/RAP1GAP/SMPD3/MAGEE1/CPLX1/AK8/DUSP6/LGI2/FAM84A/AOAH/LINC02265/LINC01503/ETFB/AZU1/DAPK2/C3orf67-AS1/MIR9-3HG/FAM222A/PCDHGB2/CYP2B7P/SGCG/RAB3C/SLC9B1/KCNB1/CPEB4/PPP1R13B/CASP16P/CLCN4/TMEM184A/TMEM130/GPR35/PPIAP49/FAM86B2/SNORD100/SLC43A1/SMAD9/ZNF534/INSL5/C19orf73/A1BG/SERPINH1P1/ASPDH/NUDT19P3/XKR9/CHRD/LINC02340/GRIK4/LINC01690/SLC22A14/RASA4DP/XKR5/CRYL1/ADPGK-AS1/CA15P1/NPM1P19/HMGB1P5/PABPN1L/TMEM163/CHRNA4/AHSG/PRSS23/ATG9B/TMEM9B-AS1/SLC6A15/BTK/LINC01208/EBF1/DNAJA4/UNC13D/PHACTR1/SNORD104/TRPA1/PCDHGA4/CAMK2A/REEP1/THSD7A/CFAP57/GCHFR/MYCBP/LINC01465/RASA4CP/DNASE1L2/LSM11/PODNL1/PCDHGA1/CCDC3/GPR27/SLC7A9/ZCCHC12/ZNF385C/PAQR9/BTF3P5/TDRKH-AS1/UBE2E1-AS1/ABLIM2/NPTX1/PLSCR5/TSSK5P/PIK3CD/SOX2-OT/CHRNA7/PANX2/NOX4/RPL22P16/DDX25/LRRC71/VLDLR-AS1/MCHR1/CFC1/IPO4/ACBD4/IFI27/GAS2L2/MAST4/CCR10/TEX14/PCNPP1/RASGRF2-AS1/GMFG/ERC2/CFAP157/IGLL1/LINC00634/SNORA33/FAM69A/C17orf113/CAMSAP3/VPS33B-DT/BLVRB/ETFBKMT/SEMA6A-AS2/ABCG2/PMEPA1/TCP11L2/MYO3B/ADCY2/LINC01852/MTFP1/LDLRAP1/UNC5CL/FBLL1/RNF224/ACTL6B/RAB29/SLC22A7/CMPK2/ARHGEF3/C22orf23/ABCA17P/IQCN/PTGES3L/CSDC2/AGBL5-IT1/AQP11/C10orf25/SNX22/STX1B/TMEM72-AS1/C19orf71/CTGF/SLC35D2/C11orf94/EHD4/UPP2/SGK1/RPL19P20/SOCS1/SNX32/SMCR2/GIPR/FRMPD2/LIN7A/FGF22/ACOT12/LINC01956/VN2R17P/LINC00205/VWA7/BCDIN3D-AS1/SUCLG2/PCDHB10/FAM85B/UNC13A/LRRC10B/RPL34-AS1/TPO/KCNJ11/LRFN1/SYT7/P2RX6/NLRC3/YIPF7/AATK/HLA-E/KIAA0513/GNG3/TPPP2/TMEM51-AS1/RELL2/RASSF3/ACAP1/FCRLB/SLC4A3/POU2F2/ZNF555/TRAT1/ISG20/PAXBP1-AS1/RPL9P29/CCDC13/FLVCR2/ANKRD53/NMBR/HNRNPUP1/PHBP4/RNF207/LYPLAL1-AS1/TTC39A/SIX5/GPR88/MIR4435-2HG/SMIM2-AS1/ZNF467/ARHGAP27/MIR181A2HG/FOXD4L1/YBX2/SH3BP1/CYP2D6/ARHGAP23/PGBD4P3/PKNOX2-AS1/CORT/AKR1C2/ACBD7/FAAH/ZNF774/CRLF2/FBXL2/CRB3/NPR2/MAGI2/SLC25A34/RAB30/PNMA6F/HAUS4/ASPHD1/PCK2/NAP1L3/NANOS1/ENDOD1/KIFC2/OSBP2/SYP/SPNS3/HNRNPA3P6/C9orf47/HCST/SFTPD/GPR149/OGDHL/TAOK3/BOLA3-AS1/TMEM253/LINC00663/CHAC2/LINC01679/ZNF883/TRAPPC5/DUOX1/PHACTR3/TMEM221/TMEM161B-AS1/ETF1P1/PPP1R14C/NPM1P27/SYT15/SP140/PCDHB14/TPRG1LP1/LIN7B/LINC01719/KCNMA1-AS1/JAG2/SYT4/RAB17/DUBR/LRRTM2/TMEM35A/TMEM63C/RPARP-AS1/GNAL/GPD1L/FOXQ1/PRKAR1B/SLC25A21-AS1/KCNK7/STPG1/RREB1/UBASH3B/CYP51A1/MROH1/TCAP/PPP1R18/MT-TI/NAP1L1P3/FBLN7/MYCNOS/ABHD1/LRRC75A/WDR31/NTRK3/FLI1/RAB33A/DUSP15/PROB1/IGFLR1/PPP1R8P1/PDE2A/NACAP1/OGFOD1P1/NAPRT/RBMXP4/PFKFB1/FUT8-AS1/PLCXD2/CTBS/WASHC1/ENPP7P2/LIPM/KCNN3/TMEM92/UPB1/LINC01711/TMEM254-AS1/OAF/RPSAP43/SIRT4/TPPP/METRNL/HNRNPA1P62/SFTPC/LINC01206/LINC00334/SNORA26/FTH1P16/PHLDA1/CLIC5/FUOM/ZNF815P/TMPRSS2/DNAJC12/TMPOP2/HHIPL2/CDKN2D/ZFHX4/NUDT3/CARMIL3/RPL23AP49/FTH1P11/NSG2/SDCBP2/ATP10D/MIR7515HG/SORBS1/KIAA2012/CGA/SPNS2/NRXN2/RSG1/TMEM178B/DNM1P35/PCDHA8/NYAP1/MC1R/RENBP/PCDHB17P/KIF9-AS1/LINC01534/PRKAR2B/LINC00326/CDH4/NOL3/ZHX1-C8orf76/FNDC10/SAMD13/NKD2/SPRED3/RAB3A/ARHGAP4/CTSC/HTRA1/SPDYE12P/RNF208/AKR1C1/PGK1P2/MTRNR2L8/C15orf59/C2orf80/BACE2/SOWAHB/TTLL11/ZDHHC15/JAZF1-AS1/NUS1P1/PRR5L/PMAIP1/IL16/HNRNPCP2/TOR4A/ZNF582-AS1/RTN3P1/C4orf33/GJA5/PCSK1N/SYDE1/DOCK9-AS2/COLEC10/CLNS1AP1/ASCL1/POU6F1/RHBDL2/MAP3K9/DAGLA/CISH/GRAMD1B/SERP2/SFRP1/MMP25-AS1/DHH/FUCA1/GTSE1-AS1/EEF1A1P34/C4A/LINC02580/FRS3/TMEM74/NPFFR2/ABCB9/CADPS/SLC1A5/ADAMTS12/CETN4P/MYH7B/LINC01405/SORCS2/MPP3/HIST1H1B/SH2D5/SCG3/PILRA/TCEA1P2/GDPD5/PFKP/RUSC2/HIST1H3G/PKD1L1/LRRC3/NPM1P35/SLC25A5-AS1/NUDT18/CACNA2D2/MIR4697HG/CDHR4/GACAT3/LINC01232/SLC6A7/THBS1/PMS2P2/TMEM59L/LINC01301/ZCCHC18/EXD1/CELF5/PHYHIPL/ATOH7/GALNT8/DPP10/CSTA/LYRM9/AVPI1/TUBB4A/LINC01186/GRIK5/RDH16/RALGPS1/HIST1H2AC/CCDC92/TEX38/LHFPL1/RSPH14/STX1A/ASTN1/NPM1P25/OR13J1/OR2B6/HSPD1P12/VMAC/DLEU1/LINC02018/KCNG1/SATL1/ATP9A/LINC01865/SPSB3/ERICH3/SNORD14A/SSR4P1/WIPI1/RPL23AP3/PRNP/RIIAD1/DHRS11/RASD1/TNS3/TPPP3/KALRN/NPM1P33/ZNF737/OVCH2/CADM3/PPIAP8/NAPB/SETP3/HIST1H2BN/RDH13/KIAA1549L/FAM213AP2/TRIM9/NEURL2/INCA1/GPR146/EPB41L1/NAP1L5/MEIS2/RBKS/KCNV1/EML2/SCARA3/OGFRP1/SEMA6D/AMN1/TPTE2P3/PITPNM2/C18orf32/CHST14/PQLC3/RASGEF1C/C21orf62-AS1/TNFRSF9/TMPRSS13/LINC01732/NPM1P24/LACTB2/OPHN1/HAGHL/CCDC65/CCDC151/KIFAP3/FXYD7/SMIM13/SNORD83A/FAM57B/CD99P1/KLF15/TFAP4/PCDHGB6/AKAP6/JPH4/CFAP97D1/RDH10/CD9/SPATA25/ADPRH/KCTD13/LINC00881/PRKCG/ABTB1/ZDHHC14/RNF182/HNRNPDP1/CLN3/FBXW11P1/MAST1/NPM1P8/HMGB1P40/SFRP5/SMARCA2/AFAP1-AS1/AGAP2/ADARB1/KHK/VASH2/CDCA7L/INPP5J/SNTA1/TPSB2/TMEM240/TRIM36/COL17A1/IL36G/PXK/STXBP1/DRC3/SNORD99/LINC01393/EPHB4/PIP5KL1/BTN3A1/HNRNPA3P5/FAM86EP/PLEKHG3/RPL7P26/EBF3/BDH1/HSD17B1P1/PDSS1P2/SEZ6L2/MID1IP1-AS1/PROKR1/PCDH12/TNFRSF19/CEND1/HIST1H2AG/OLFM1/SLC2A11/SLC25A30-AS1/DPF1/GTF2IRD1P1/TMEM121/ZNF697/DENND3/SRRM3/OR8T1P/NSG1/ASPRV1/DOC2A/SNAP25/MKLN1-AS/SMAD6/RTN1/EPB41L3/IGBP1P4/HIST1H2BD/MYO10/CAMKK1/ANK2/CYSRT1/ENHO/LINC01426/NPM1P46/KIAA1614/PPFIA3/PRKG1-AS1/KRT18P59/GNPTAB/RUNDC3A/EAF2/TMEM268/PRRT3/PRKCE/GNRHR2/CHN2/PHF19/ANKRD20A10P/PIK3IP1/SFXN3/NTRK2/CT62/LZTS3/GYPC/TBC1D24/MYRFL/TMEM246/ZNF806/DBF4P1/NPM1P5/CENPS/INA/KIF21B/JDP2/NPM1P12/LINC01144/TSPAN7/RIMS4/PMS2P4/REEP4/DUSP14/HAL/OR1F2P/AVIL/CNKSR1/NR0B1/ELAVL4/TMEM158/LIPE-AS1/HMGB1P11/EHD3/GUCY1B1/C11orf70/EPHA1/ACKR2/DTX1/KANK2/PKDCC/CDK5R1/RAB41/MOV10/SARS2/NKAPP1/ASGR1/CERS3/DEPDC7/FITM2/NPM1P48/TNFSF4/UCN/SAMD14/LGALS3BP/MAP3K20-AS1/HPCAL1/WNK2/MTURN/TMEM51/ANTXR2/TMEM86A/SETP14/HSPG2/PAK3/SHD/NME9/KCNIP2/ASTN2/TRNP1/HIST2H2BE/PITPNC1/WDR88/HIST3H2A/USHBP1/METTL25/HLA-L/KNDC1/BORCS6/CASP7/SCEL/PLAUR/RPS6KL1/KDM4C/CACNB1/SAMD10/PPP3CC/DNAH1/DNAJC9-AS1/FAM20C/RPS10/PDP1/MIR181A1HG/ARSD/KLHDC9/CCDC113/AFAP1/IQCD/STMN4/FAM21EP/TAGAP/VSTM2L/SPEM2/EDIL3/SLC37A1/FBXO36/TRAPPC3L/MAST3/SVOPL/C2CD2L/LINC00987/PTPRG-AS1/MISP3/CMBL/CAPS/GLIS2/ST3GAL5/THBS2/IRF7/ABHD15/CCDC183/PSMG3-AS1/LINC01132/OPTN/MANSC1/SULT1C4/STKLD1/ALDH5A1/PZP/ATL1/KCNH2/ITPK1/ADCK2/CD3EAP/RPL7AP14/RAB11FIP4/LITAF/LINC01901/PGM2/CAMK1D/MCMDC2/SRRM4/PTGER2/KIAA1324/SLC1A4/LINC01128/GMDS-AS1/CYP21A1P/AP5B1/FBXL16/LINC01165/PARD6A/VPS37D/MXD1/DACT3/TRIM3/SNHG3/MMP28/C18orf65/LINC01670/LRRC23/PLEKHM3/ZC2HC1C/DNER/FNDC11/IFI6/GDAP1L1/LINC00921/TSPAN12/ANP32D/PCDHA7/TLN2/MRPL45P2/GNG7/CEP170B/FAM124A/DNLZ/NDUFA7/WDR66/OLMALINC/GIPC2/SUMO2P17/CMTM2/EIF2S2P4/TOLLIP-AS1/TRIM73/WHRN/TIGIT/PLEKHG5/HIPK1-AS1/C11orf71/NKD1/SIGLEC16/CDH17/PSD/LRRC55/KIF3C/MANSC4/MDGA1/SMCO4/TRPC5/SUGCT/PSMD5/GDPGP1/ARL4D/IL10RB-AS1/VWA5A/CYB561/OMA1/DOCK6/EFR3B/FAM228B/RPL7AP31/SEPT5/LINC01135/SOX9/INO80B/SPRYD3/ABHD8/SPDYE18/ARL2BP/DOK4/VSIG10L/LMO3/NRG1/NACAD/ADAM8/LINC01134/ADAMTS10/ATP1A3/TEX30/DLK1/ANKEF1/BCL9L/THRA/PKIA/PDE4D/SOBP/TTC6/FDPSP8/PLEKHA4/SNX19P2/GARNL3/ZNF793-AS1/HID1/ERC1/MAN2B2/HNRNPA1P39/HNRNPA1P55/NOS1AP/POPDC2/SLC25A18/A1BG-AS1/ABTB2/AMIGO1/OLFM2/RCL1/FAM110B/LIN37/C17orf100/IDS/RPL7AP26/MTHFD2P7/SYT1/PLIN1/PDE4DIP/SAMHD1/FAM110D/C15orf41/ZNF98/LINC02133/CKS2/ARG2/LINC01829/FAM86C1/HSPD1P1/TRPM4/NFASC/MYOSLID/NECAP1/FAM241B/HSBP1L1/AP3B2/DCX/TMCC2/PINK1-AS/APC2/THAP7-AS1/CCDC24/IGBP1P1/SEMA4G/TSPOAP1/LINC00458/ABHD6/CTIF/TECTA/UGT2B7/DIRAS1/RBPMS-AS1/LINC02389/PIRT/LINC00473/RRAGD/LINC00616/SH3BGR/FBXO44/PACRG-AS2/CEP19/PPP2R5B/DICER1-AS1/LINC01003/PRR36/MAPK8IP2/PSTPIP2/MAPRE3/RTN4RL2/EEF1A2/DRAXIN/HNRNPA3P3/IL20RB/ANXA9/GAPDHP33/MTSS1/IL5RA/SPATA2L/ABHD14A/NOMO2/C1orf50/NCS1/YBX3/SLC9A6/ATP1B2/LCMT1-AS1/C16orf95/GAP43/COCH/TMEM156/FAM174B/SLC43A2/RARG/KRTAP5-9/SIAE/SYBU/IQCH/KLHL13/CELF3/CBX7/USP2/ELAVL2/PDE3A/GPR26/OAZ3/AGAP1/DOCK1/ABCA3/LINC00899/CRB2/GABRG1/STAB2/EFCAB11/MAP3K10/LINC01483/MICALL2/PGAM1P8/LUARIS/ZFR2/MANEAL/SH2D1A/WDR47/TOX2/PLXNA2/DENND2D/BOLA1/GPSM3/ASS1/SERBP1P1/TRIM74/H1FNT/ATCAY/PRR7/PINK1/SH2D3C/RHOG/LINC00924/PSPN/C9orf43/PLPPR2/CHST15/DACH1/HNRNPA1P50/OXSM/YPEL3/C16orf45/KCNAB2/ATP6V1C2/RHEX/PIP4K2B/HNRNPA1P60/CBX6/FSCN1P1/LEMD1/CDC42EP2/WASH3P/PLA2G12A/IRGQ/SULT4A1/BAIAP3/INF2/WISP2/SMAP2/GPM6A/RHBDF1/TCF7L1/NRROS/LRRC56/MAGED4/GNB5/RAPGEF4-AS1/SLC35E4/MLNR/PRSS27/ABCG1/NFKBIL1/MAFF/COX11P1/HADHAP1/RHPN2/PPP1R3D/AIG1/SLC9A2/MICAL3/ASPHD2/UBR7/SDCCAG3P2/DPH6/ZBTB7B/AK9/NEURL1B/LINC01185/TMEM150C/IL27RA/RAB6B/WWC2-AS2/ZFPM1/TMEM45A/FRMD5/HSD17B1/SEC14L2/ABCA7/RFK/BEX2/SPEG/TRIM46/RUBCN/SETP12/HDAC5/MON1A/FBXW4P1/ALDH16A1/NAPA-AS1/CYSLTR2/ITGA5/WBP1LP2/TMC4/EFNA3/LINGO1/GPRASP2/MCOLN1/PPP1R11/ZSCAN12P1/IGLON5/GABRA6/CHST3/GABRA4/LYPD6/CCND1/PRPF40B/MACC1/GABRB3/SLC12A6/HTR5A/KCNE1/DUSP1/TTTY15/SNAP23/ETNK2/L1CAM/MRAS/HNRNPA1P9/ZNF536/AVEN/TEX19/BTBD9/OR2W6P/ANP32AP1/ADD2/LINC01470/IRGM/KIF1A/FN3K/WDR86/PNPLA3/GPR52/HMOX2/KRTAP5-7/TP53INP2/SLC38A3/NDUFV2-AS1/ABCD1/FHOD1/TUB/PPP1R12B/OTC/SMARCA5-AS1/ZNF890P/CLU/FDXR/BRSK1/HECTD4/GRK4/TNFRSF17/PLLP/B3GLCT/TTLL11-IT1/C2orf91/NUDT8/SPANXA2-OT1/DIAPH2-AS1/SV2A/RAD23BP1/SOX2/TMEM81/CHD3/LRFN4/YPEL1/OR2C1/MEGF9/FNDC8/CHIT1/DDX11/PGPEP1/ACVR2A/EVI5L/AKAP3/KIF5A/SHPK/PBX4/SCAMP5/OR2K2/SNCAIP/TMEM175/RAB6D/SLC9B2/TRIM16L/FAM86FP/TMEM109/SNHG16/ZDHHC1/CMTM6/DENND6B/ATP1B1/NFIL3/GLCE/EIF4EBP1/KCTD7/FGGY/GLRX/WDR4/GNAO1/TEAD2/NOXO1/RIDA/NCAM1/ATAT1/RAB15/COQ2/ATP13A2/DCC/RPL13P5/NMNAT2/TREX2/C2CD4C/TMEM143/MLKL/EXD3/RTN2/ASIC1/CAMTA2/GAR1/C9orf129/SNX33/LHPP/GLB1L2/ZNF236-DT/TSNAXIP1/MLLT6/MAML3/FEM1A/FAM227B/PPIAP46/C11orf72/RBM15-AS1/NYNRIN/APEX2/PRELID3A/SLC27A1/KLF1/C20orf194/MAFK/UCP2/PARVA/FBXO25/SCHIP1/CLIP2/C10orf95/RNF128/SARM1/LINC01006/C4orf36/SNX30/PIANP/GPR150/GRID1-AS1/PRSS55/CCDC184/MMP17/HNRNPA3P10/EPHB1/AIFM2/SLC46A1/ZNF554/SLC22A23/MVB12B/PELI3/LINC02210/RWDD2A/PPRC1/BACH2/BCRP2/PET117/STK32C/MIEF2/DOK5/JMJD6/CICP22/TMC3/ARSA/NT5M/LINC01094/SLC35F6/CNOT6LP1/PRKCZ/OSER1-AS1/RP9P/TAF1A-AS1/ZFAND4/CDK20/FRG1BP/HNRNPA1P32/LBH/MGAT5B/TMEM192/ADGRB2/IER5/TOMM40L/KIAA1211/GBA/STEAP3-AS1/SCARF2/MACO1/ORAI1/GNAZ/GPR162/GALNS/STARD10/ADGRL1/SYNGR1/IGSF3/SLC35G2/ZADH2/NXF4/SNX5P1/RAVER1/CSPG5/ALLC/CPLX2/STRCP1/PMM2/IRF2BPL/UBE2D4/VAMP2/SPACA9/NIFK/MYO18A/ELAVL3/EIF2S2P3/RIMS3/VPS9D1/GNAI1/TFB2M/TENM4/ACOX1/PACS1/NEDD4L/SPRYD7/LPCAT4/ATP6V1A/EIF4A1/RPL23AP7/SNHG17/CD200/CRYM-AS1/IQCE/MCF2L/RXFP2/CRTC1/CYFIP1/ZDHHC24/DGKA/CTNNA2/GABARAP/MOK/C3orf14/U2AF1L4/AEN/ZNF497/FXN/ATG4A/DLX3/LINC00528/CCDC120/ATP6V0E2/FAM185A/CXorf40A/GAS8/ZNF324B/MINDY1/MEF2D/SLC25A13/RPP40/TOM1L2/DNASE2/GTF2IP4/FUT10/SLC4A8/PCDHB2/DBNDD1/ARMC5/FIG4/SYT17/GRK5/SMCR5/C19orf57/FAM131B/RIN2/C14orf39/SLC22A17/ZNF511/NCDN/LINC02395/MGAT5/MT-TL1/PAQR7/SLC16A2/CBY3/DNAAF5/ZNF34/DUSP7/NKX6-3/AMZ2P1/CMTM3/NR2C2AP/SCD5/PA2G4P1/PNP/LINC00323/KIF3B/BORCS5/MPP2/PEX6/TMED8/CMIP/ULK4/VLDLR/SSH2/PAICSP4/FCHO1/MTHFD2/SMN1/CAB39L/DKK3/ANKH/GMIP/RPL17/ZAR1L/LYSMD1/GNG2/SLC17A5/HSD11B1L/SDK1/DTNB/AK1/NDUFB2-AS1/FAM219A/CCDC58/NKIRAS1/MROH8/DNAJC5/FGF20/GRAP/HNRNPCL1/ARHGAP1/VN1R5/PPP1R3F/MAP3K13/FDPSP1/LINC02048/ARHGEF9/NFKB2/GDPD1/SERPINE2/RNF19B/HNF1B/LINC01562/CTDSPL/ZHX3/TPT1-AS1/TANGO2/B3GAT1/SRP14-AS1/C9orf3/MEGF8/SDCCAG8/MARS2/9-Mar/REEP2/SLC26A8/NSF/HNRNPA1P45/MGAT3/TCEANC/TMEM14A/MBOAT7/PWWP2B/ABCA2/BTBD6/CLTB/CASP10/PXN-AS1/CNTNAP1/MAPRE2/LINC00963/TMEM38A/ELMO1/RRAGB/TBC1D25/ESPNP/SLC36A1/LINC00602/MAPK8IP1/TMEM8B/TAT/EID2B/DCHS1/C21orf2/MTHFR/NPM3/CLSTN3/LINC00294/MAPK4/NF2/NUTM2HP/LPO/SLC25A4/SDC1/ST6GALNAC1/SLIT1/BIN1/SEPT3/SPATA20/ZNF529-AS1/FMN2/MPPE1/LINC02458/KCNK15/VAV1/CCDC63/ARHGEF18/ANKRD46/VPS26B/PLEKHH3/IL11RA/CHST11/FNDC4/IGSF8/DDB2/ZNF579/GC/CCDC159/MORN4/NDRG4/FAAP24/TMEM62/ATP6V0A1/CYSTM1/STIM1/NBEAL2/LINC02346/ARHGAP39/GALK1/LRP1/ROBO1/FAM171A2/GSTO1/C1QTNF8/DLG4/PNMA8A/SLC29A1/SLCO3A1/GAB2/NOVA2/SLC48A1/LRRC4B/NSMF/GNG4/JAM2/E2F5/OCRL/SEMA6B/MAPKBP1/DSTNP2/FAM49A/ZNF324/C6orf136/PTK2B/LINC01612/ZNF837/SOX4/THOC3/FGD1/KREMEN1/FAM120B/FKBP1B/ZBTB46/TMEM127/MLLT11/FAHD2P1/CXCR4/SMAP1/C12orf75/LGR6/KDM8/ZNF653/HDAC11/NAT8L/AHDC1/GAS6/CCNB1IP1/MICALL1/CASP6/ENO2/CELSR3/EME2/UBXN8/USP35/EXOSC5/CBLB/SPAG16/CPTP/WSCD1/GCAT/PHLDB3/RAB11FIP5/EEF1D/TMEM63B/SLC25A12/FADD/FBXO31/FAM177A1/PAXIP1-AS1/ALDH1B1/EXOC3L2/TMEM120B/MFSD13A/CTNND2/FOXRED2/APBB1/KCNMB4/BUD13P1/PA2G4P2/HIP1R/SLC35E1/MYLKP1/DEPDC1-AS1/PLG/ESRP1/ZNF208/MFAP2/TIGAR/NOP14/KCNQ2/SMARCD2/NOP14-AS1/DGKD/WEE2-AS1/LRSAM1/WSB2/FKBP4P1/ABI3/ARMCX1/SUSD6/RHOBTB2/RGS2/ZNF783/PORCN/AZIN2/STMN2/CHPF2/MINDY3/LINC00167/CERS1/UBA6-AS1/TMEM25/GPR153/PPIP5K1/EEF1A1P28/PPP1R15A/CBARP/HORMAD1/ZNF436/NIPSNAP3A/ATP2B4/PPP1R21/PLPPR3/PBX3/SRD5A1/GAPDHP68/R3HDM2/SZT2/SULT1E1/LETM1P2/C3orf18/SNHG10/LARP6/TRIB1/TMCC1/RPS6KA2/MAST2/CRMP1/CASTOR3/PRKCZ-AS1/DGLUCY/THAP8/KIAA0930/OSBPL5/CRY2/KSR1/LRFN3/CCDC71L/RFFL/CTXN2/IBSP/WDR37/DGKQ/CLUHP3/WBP1L/HDAC4/PLD2/ERLIN1/PPP1R9B/BBS9/CCDC32/PIM1/ZNF319/CA11/LINC01558/DUSP28/BRD7P2/SEMA4F/ARL8A/EMID1/APRT/GNG5/MFGE8/RPTOR/ORC5/BDH2/KIAA0895L/GSDME/TUBB2A/BCL2L11/GPN3/ACAT2/AKAP17BP/C20orf196/TIMM8A/URGCP/DAB2IP/VOPP1/SERINC1/CADM1/RHBDD1/TBC1D22B/SCPEP1/SLC25A33/NLRP1/UVRAG/SLC26A11/ECE1/AMY1B/PNO1/FAM109B/SLC39A14/FBXL7/FLYWCH1/ADA/CBX8/LINC01990/ZKSCAN7/JAK1/IQSEC1/MSH5/RTL6/SLC14A2/C14orf132/B4GALT4/SLC35C1/DNAJB2/LINC00347/TTLL1/APBB2/NUDCD2/ZNF446/NCAN/SEPT10P1/CUL9/TMED1/BRF1/ATXN7L2/ATN1/TBC1D19/DEUP1/BET1/MAT1A/PACS2/TLE1/SMTN/KLC4/RAPGEF1/RFX2/ORAI2/KIRREL1/PTRHD1/SLC26A6/UBE2E2/SPTAN1/APLP1/LDLRAD2/HDDC3/CPE/RGL2/CNIH2/APIP/LHFPL6/COMMD10/LPA/TOMM34/MRPL45/ZSWIM8/CETN3/RAB34/ANKRD13B/ZFP36L1/SMO/ROGDI/IL10RB/LINC01800/TMEM39A/HK1/HCAR2/MYT1L-AS1/PLXNA3/VSIG1/SMYD2/ALKBH6/PICK1/PIGV/TERF2IP/IL9RP3/IPO7P2/PNRC1/CDK7/LINC02144/TMCO6/TJAP1/COTL1/WDR24/KLHL25/PRDX6/ANKRD39/PXYLP1/MTHFD1/PINCR/MAP3K12/NALCN-AS1/PPP3CB/STX12/NR2F6/ZNF865/HHLA1/MTHFD2P1/OCEL1/RPL36AL/LRP3/ZER1/MZT2A/P2RY11/GJB3/HIBADH/FAHD2CP/ZMIZ2/NFS1/AKTIP/TSEN15/TTC27/CISD1/PIP5K1C/PUSL1/PIGF/FUK/CXXC5/SYT11/ITGB1/SIAH2/EBPL/KIF7/MARK4/FAM160B2/FMNL3/ASPA/MMP15/THG1L/FRMD7/EFCAB6-AS1/PFKFB4/BCL2L1/PLA2G6/TCEAL3/MAPK8/TMEM182/TSPAN17/KDELC1/ZDHHC9/ZBED1/NAGK/TFB1M/MROH2A/BEX1/ZFYVE27/LATS2/SLC25A44/MTHFD1L/TNK2/UPRT/LINC01182/SLC18B1/ALOX12-AS1/ANKS6/FAM3B/ABCC10/DPH2/FAM122A/NCOA4P2/PITPNM1/RXRA/FAR2P3/ANGPTL5/CRIP2/CHST12/GPR6/AMBN/CIC/LINC01278/ENC1/MCOLN3/STRADBP1/RPIA/PXDN/STAT3/PDXDC1/ZNF500/C14orf37/LAP3/SRF/PAX1/SEMA6C/HDAC7/ZFYVE19/GPR173/DPYSL3/RPS27L/GSTA4/GM2A/GRAMD4/FAM109A/ST7/SHC2/TBC1D1/PABPC3/ZRANB1/LINC00940/MYO1C/ARHGEF17/RNF11/PAK4/TECPR1/ZNF517/IFNGR2/SLC29A2/RND1/CLIP3/ABCB8/GSTM3/KCTD21/ANKRA2/TUFT1/TPRKB/FBXL20/PALM/GRAMD1A/PI4KA/OTOA/MTHFD1P1/GTF2H5/SMPD2/RERE/MGRN1/CLDN15/SLC25A25/CTC1/PPP1R26/PDE4DIPP1/MTMR4/KCNJ1/ZNF627/PHF2/VASH1/PSEN2/ERF/WWC1/NOB1/MAP2K5/PDXK/CDADC1/DGKZ/BPIFC/LDHAL6B/HGSNAT/NUMBL/CITED2/EPN1/ALG8/SRSF8/ERBB2/MOB3A/THTPA/SLC29A4/CLDN23/LRRC63/IRF2BP1/LRRC20/ARMCX5-GPRASP2/SARAF/MT-RNR1/OBSL1/PDLIM7/HHLA2/NOP16/SLAMF8/LPXN/MAP4K2/ST20-AS1/C6orf89/C12orf76/PNPLA6/ATG2A/FAM222B/PROSER2-AS1/WDR13/OAT/HMBS/CRAT/CYP20A1/NAT6/RAB6A/IMPDH1P6/ZNF250/TRIM65/EFNB3/ZNF561/SLC26A9/C7orf43/SMIM30/YARS2/ZNF219/PTPN18/NANOGP1/TASP1/GPATCH4/2-Mar/GALK2/CACFD1/TOP1MT/PLEKHO2/ACTL8/ZNF414/CCDC190/DNAJB5/INPPL1/LINC01567/MNT/ACAA2/DHRS1/DTX3/RNF123/MKL1/PFN1/PITRM1/EPN2/SBF1/RTL5/MFSD12/TRAF3/SLC20A1/CCDC115/AHCY/RIPOR1/TWNK/ARHGEF10L/ALKBH2/FAM131A/PDCD10/RAB2A/CYB561D1/DNAJC4/LRRC28/CCDC137/RNF26/MFSD9/MXRA7/RAB40B/NDRG3/POLR1E/ALG9/ZC3H3/RTN4/NUTM2B-AS1/AKT1S1/SIDT2/MPHOSPH6/RAB11B/ADGRG1/CELF6/FXYD6/CAMK2G/RALGDS/CLCN7/BASP1/DHRS4-AS1/AP2A1/GIT1/MNAT1/EXT1/UBE2F-SCLY/ENDOV/CERS4/SMYD3/AHCYL2/MVK/LRRC53/ECI1/CASP9/TUBB2B/ACOX3/RAB6C/ARRDC1/TM2D3/HMGN3/IDI1/TBKBP1/ACTN1/CBFA2T2/GNS/HSDL1/RFNG/CPT1C/DEDD2/RMC1/DEAF1/WRB/DUS1L/C8orf59/SLC25A42/CHMP1B/INAFM2/NLRP11/LINC01936/SHMT2/PACRG-AS1/TRAPPC12/GSTZ1/RAC3/HDGF/FAM90A26/ACOT8/TADA2B/GNA11/SLC50A1/SMAD3/CDAN1/ARHGEF25/SOX12/CTSB/OSTC/AP1B1/ATIC/CHMP1B2P/NSA2/ELMOD3/PIK3R3/TMPRSS3/C8orf86/EXD2/ZNF205/LINC01616/C1orf131/KLHL22/DALRD3/GAREM2/TMEM185B/NOTCH3/ST3GAL3/CTSL/MINPP1/ZSCAN18/ZNF358/NUDCD3/AMBRA1/CRX/PTRH2/SGSM2/BAIAP2-AS1/PPP2R3C/DDX31/PHF1/ZC3H7B/FGFR1/NME4/C8orf82/FAM214B/ABHD17A/ACO2/WDR91/BCAS3/TIMM10/VAT1/MAP1LC3B/PTPN23/C12orf73/MIF4GD/DDX54/NIF3L1/ST6GALNAC6/C18orf21/KDM4B/FAM210B/GABARAPL1/STMN3/RBM18/GCDH/TATDN2/C1orf35/DHODH/RPP30/FSD1/NSL1/POMT1/RNF14/TBC1D10B/ASB8/KLC1/PPARD/BRD7P5/TPRN/PPP1R37/PRR29-AS1/HSF5/PLEKHB2/SORT1/LRRN4/MPC2/RAP1GDS1/SLC9A1/ADGRE4P/ARHGAP17/SDR42E2/SLC25A22/FANCG/LZTR1/FAAP100/COA4/TMEM132A/EIF2D/TYRO3/RNF187/ST3GAL2/TCEAL4/CAPN1/NARF/EPC1/CAND2/RBM3/ICMT/ZNF316/DHCR24/AMFR/GALNT11/SLC38A10/NDUFAF5/ZNF804B/PRKACA/SEC61A2/GLO1/CLNK/LRRC27/CHCHD4/CCDC127/TATDN3/SLC39A13/HSP90B1/ARL4C/MAGI1-IT1/KHNYN/MAP1S/RTN3/GTF2F2/CD163/SEPT9/COQ6/FAM220A/FAM224B/FBRSL1/TMEM75/ASB6/RBM19/PBDC1/ARMC9/PLN/VPS16/NEURL4/LIMK1/SNRPA1/MYCT1/DCTD/SIRT3/SRC/DPH1/DVL1/TNPO2/RABIF/ADGRF1/FBXO8/PIGH/PAM/FAM160A2/TTI2/FREM3/TRIB2/RUNDC1/TMEM189/CES2/NPFFR1/ABAT/ANAPC10/IP6K1/NDRG2/MFSD10/RAB40C/TMTC4/IPO13/BTBD10/PSMA2/PEX11B/INPP5E/UNC119/ELL/SCRN1/ARL6IP5/BPGM/LINC01123/RNF215/CYB5B/LTA4H/FBXO34/MEAF6/PHKB/ZCCHC3/PPP2R2D/TRAK1/DDX39A/TMEM138/INPP5A/SLC25A23/FEZ1/FOXD4L4/USP30/TTC13/SMS/SLC19A1/RFT1/ZNF18/ZNF830/TOR1B/FAM136A/WASHC3/SMARCC2/WDR74/PRMT2/AP1M1/TXNDC11/UTP18/MLLT1/MTX1/SYF2/PLBD2/NGRN/NT5C/TYW1/SRR/CAD/SPATA2/TKT/SPPL3/TBC1D2B/GRM5-AS1/NDUFA9/LMBR1L/HMGB1/METTL17/DENND2A/TKTL2/MRPL16/QSOX2/AKAP2/KATNA1/OGFR/TM2D1/ATG16L1/SIN3B/HIGD1A/SCAP/FDPS/SPATA5L1/VPS18/EEF1B2/GPC1/USP20/MAPK7/C11orf49/NEU3/C7orf26/GNA12/MINK1/SSBP2/BCDIN3D/CUL7/DMP1/ISCA1/DGCR2/TRABD/PIGQ/MEIS3/FAF1/RPUSD4/PPP3R2/POLB/MXI1/PYGB/SH2B1/KDM5B/FGD5P1/MAPK8IP3/SCP2/GGT7/GOT1/OSBPL2/ZNF2/MRPS34/NISCH/TRIM8/CPXM1/GRK2/EZR/GDI1/HEXA/ARAF/LINC02338/FBXL12/ZNF580/CFAP36/PABPC1P4/YPEL5/RGS13/ARMCX6/MALSU1/SHISA5/C1QTNF3-AMACR/CNOT3/PKN1/TCEA2/STRIP1/UXS1/C9orf85/CLSTN1/ZDHHC13/RMDN1/COA5/PPT1/PSMC1/SERINC3/ZFAND2B/TRO/DDX24/GSTP1/FBRS/SCD/TLR10/USP22/ADCY6/GSR/AES/PI4KB/TTC8/ABCF1/DFFB/E2F4/MMAB/GLDCP1/METAP1/STAT5B/DMWD/PCMT1/GABARAPL2/TMEM259/TTYH3/PTPRD-AS2/KAT7/TSC2/PCA3/SH3BP5L/DOLPP1/CALCOCO1/TCF3/PARP2/ITIH6/MRPS7/SCAMP2/THOP1/LAMTOR3/WIPF2/CNOT11/TRAF7/SH3GLB2/FBXW5/CSTF2/RGS12/IFT140/CERS5/ASCC1/GUCD1/SUN1/STK11IP/LCT/MESD/DENND4B/TOB2/TRAPPC2/RABGGTB/STARD3NL/SCAF1/INO80C/ANGEL1/SELENOO/SLC28A1/MROH3P/PPP6R2/SLC25A26/CNN3/DDX56/COG1/DDR1/METTL3/PRDX3/PCYOX1L/EXOC7/CTNNBIP1/MAP3K11/SLBP/B4GALNT4/ITM2C/BSDC1/NCK2/USP11/DARS/FZR1/GMEB2/UNC50/AKAP8L/WRNIP1/PGP/ANKS3/CACNG7/RPP38/MOSPD1/LINC01516/SPIDR/ZBTB48/TEX13D/DUT/STAT2/EIF3M/TTC38/ATP6V1B2/RARS/SF3A2/MKRN1/MGAT4B/WDR12/CLNS1A/SYVN1/CTDSP2/SULT1B1/PECR/PML/CCDC9/PDIA3/ZNF584/PITPNA/SCYL1/CAMLG/PAAF1/ATP13A1/MAPK3/NCALD/TINAG/CYHR1/JUND/SLC41A3/MRPS10/RCN1/CHFR/ADD1/MRPS27/PFKL/RCC1L/TRIM28/VIPAS39/RIMBP3/ARPC5/PLXNB2/PIK3C2G/PPP6R1/RNF10/PCBP1/RSL24D1/CHMP7/RNF216/AKAP8/SNX19/CSK/ZNF330/AP3D1/SAMD1/ZNF394/ZSCAN4/ZNF346/SMPD4/RRP36/RAB35/EPYC/VPS33B/ABL1/CALM1/TUBA1A/SH3PXD2B/DCTN6/SEPT2/SLC9A4/ADPGK/GAK/ANAPC16/VPS11/ACSF3/ELAC2/PITHD1/KBTBD12/FHL1/MLH1/LRRC47/TRMT1/YWHAB/CPSF4/FAM120AOS/RBM14/ERGIC1/SF3A1/MRPL3/RTP5/MS4A7/MRFAP1L1/CDC42/LINC01109/KRT18P33/NAXD/CSNK1E/ZBTB17/GANAB/STRN4/RBCK1/NDEL1/ZNF174/TMEM59/IL22RA1/COQ7/CHMP1A/RANBP10/CDC42SE1/PLEKHM2/PPIE/CACYBP/PABPC4/PCBP1-AS1/PPME1/CSNK1D/NOL7/SUPT5H/NLGN2/TMEM184B/VAC14/TNFAIP1/CSTF1/CALM3/RMND5B/CCT5/TIMM17A/FNTB/PAPSS1/CPSF1/HGS/SHANK2-AS1/CNPY3/MPI/CD93/DCTN2/CHMP5/INTS14/U2AF2/SNN/MTIF3/PTPRA/PRMT1/RAB4A/ZNF282/CYTH3/DVL3/CCT4/GLG1/AP3M2/EFTUD2/RAB1B/TPM3/BTBD2/VPS39/HECTD3/NCMAP/PPM1G/GLUD1/NECAP2/DMAP1/MRPS22/PARP1P1/NMT1/TMCO3/TSN/ZC3H18/SDCCAG3/NPHP3-ACAD11/GBA2/TMEM183A/KLHDC2/HSD17B4/RMDN3/RSAD1/TIMM44/CHMP4B/MED15/RABL6/ALAS1/CDK16/PPIL2/KIAA1191/NDUFA10/PEX14/PNMA1/DBNL/KRTAP5-8/EIF4A2/FBXO21/IMP4/DDX19A/CCR4/MRPS14/CENPBD1P1/PGD/GOLGA2/DVL2/ANAPC7/DEF8/ATP1B3/OGFOD1/AAAS/ECD/METTL13/MBTPS1/ATG13/AKIRIN2/PHF13/PSMD11/SART1/COG7/YWHAQ/TAB1/ELMO2/TRAF2/ZFAND3/KARS/PSMD10/EIF2B1/HNRNPA1P54/EXOC3/INTS11/COPG1/KLHL12/RAB5B/PKM/ATG7/STARD3/HYDIN2/VAPA/HSP90AB1/MFF/FAM71E2/RDH11/PGS1* | *PAX8-AS1/TRPV2/LRRN3/CYTL1/LINC01139/PCOLCE2/CACNA1H/COL8A2/NHSL2/GABRE/CDH6/ADAM12/DNAJC22/S1PR5/LINC02998/AGTR1/KCNK1/CYP2S1/EPHA4/LOC105377896/ELFN1/PAK3/MACROH2A2/MANBA/ABLIM3/PLXNA4/KCNN2/NAAA/JMY/EPHB1/JCAD/NTF3/ZNF717/PLA1A/GRIP2/SRSF12/MFGE8/C19orf33/SLC6A6/URB1-AS1/SNTA1/SCARF2/MGLL/RBPMS2/ITGB1BP2/PORCN/LOC105374317/SLC4A7/NPTXR/LINC02381/CD93/ALDH1A3-AS1/IRX2/PTPN3/KCNIP3/LINC01004/CCDC68/LINC00623/PITPNM3/ECM1/CARMIL1/TIE1/LOC105370670/CACNB2/STAC/GGT1/GYPC/ARHGAP28/GSDME/VAT1/PABPC1L/EPB41/NALCN/ZNF521/NTNG1/RNF207/KHDC1/RDH5/CYP27C1/IDUA/MERTK/KCNQ3/TCEA3/GRIP1/FOXP4/PALM/CAVIN4/METTL7B/MEGF10/CORIN/PRSS12/ZNF677/C1QL3/LOC105371654/MAPK12/CCDC188/PRKAA2/RNASET2/ETNK2/STK26/CASP9/EMX2/KCNH1-IT1/WFIKKN2/FAM184B/GOLT1A/OPN4/FAM169A/ADGRF2/CST3/MYO1B/GGTA1/MOXD1/GPR157/LOC105375980/HAGLROS/RGS1/SMLR1/SSH3/STEAP3/LOC105375124/LOC101927078/MBNL3/RNF180/TFEB/HSD17B2/LOC105370965/TNS3/ARRB1/MT3/LINC00869/CMTM8/CEACAM22P/LOC105376214/TREM1/HTATIP2/GALNT10/CLK4/C10orf90/TMEM54/TALDO1/KDM7A/NDUFA12/POMT1/HSPB1/ZDHHC11/GPER1/URB2/MSRB2/ALG10B/LOC105371874/FXYD5/ZNF568/LOC105376039/C10orf105/ZNF829/XKR6/LINC02511/SPOCK1/LINC01605/LYSMD2/IGSF9/ZNF778/CYB5R3/RHBDF1/P4HTM/IGSF8/FCGRT/UBXN2B/ZSCAN31/STAG3L4/COX17/SLC27A1/ZNF286B/CCDC112/TMTC2/RNPC3-DT/RPH3AL/PNPLA7/PALS2/IL17RD/KCNAB3/LOC100130872/ADRA1B/SURF1/CAPN5/ATP2A3/EBP/STEAP3-AS1/SUGT1P4-STRA6LP/LOC105379241/LOC105373581/CATSPERE/MTLN/LOC105377861/STYX/LOC107986537/NDUFA4/LOC728158/IQSEC2/SUGT1P4-STRA6LP-CCDC180/MGST2/ICMT-DT/GLMP/LOC107986697/VAMP8/ARNT2/ARHGAP25/MIRLET7F1/TXNDC15/MARCHF1/GGTLC2/TRR-TCT1-1/RPE65/LOC107985404/LOC105377871/RBPMS-AS1/SASS6/ROM1/ARMCX3/LOC107985563/SEC11C/DHRSX/PRDX6/LOC107985594/LOC105373570/MYL6/MATCAP2/XRRA1/SCAND3/RIMS3/GAPDH/LOC646762/FSD2/SLC9A3-AS1/ITFG1-AS1/LRGUK/PHPT1/VPS29/PARVG/PDE4DIPP6/LINC02044/KIAA0319/TRIM52/BMS1P23/AP5M1/HECA/LINC01515/ERI1/TRAPPC5/ZFP69B/CREB5/ZNF594/POTEF/CYP2B7P/LOC100506124/HCFC1R1/DYNLT2/PPP1R3G/SLC44A2/ZDHHC12/BLVRB/EPN1/SLC2A4RG/PIP5KL1/LOC105375457/DPEP1/RNASEK-C17orf49/PGAP6/FN3K/FAM88B/FDX2/RIMKLB/SELENON/IL13RA1/VKORC1/SNX11/GPSM3/CAMTA2-AS1/LINP1/SLC25A27/CFAP90/SUMF2/CTSA/ARSA/CBX8/EXOC3L2/STX8/MIR4668/ANXA6/CBFB/SLC2A14/MOCS2-DT/SERF1A/CERS6/AK1/TMEM14C/KLF7/FAM114A1/LRRC34/LOC105376204/LOC105377873/CCDC180/CCDC159/TPST1/LOC112267914/LOC105378542/ZNF879/GALNS/BRSK1/MED15P9/NAPEPLD/SNORD140/SLC15A2/RGL2/LOC105377929/SHOC1/YIF1B/GALK1/SEMA6A-AS1/CALML4/CACUL1/SCARB2/COX7A2/ZNF26/LINC01315/PRMT6/ECI2/LOC100130331/LOC107987373/MEAK7/DCAF4/ABCA2/SERF1B/ZNF302/MIR4453/LOC107984243/SHKBP1/HIPK3/DYNLT1/B3GALT1/LOC101060553/OGT/CHCHD5/COASY/GGT7/LOC100129534/LOC105369812/ZNF888/TWNK/ACTA1/C6orf226/HSD17B8/DPP9/MRPL58/ACTB/B3GNTL1/FAM167B/DNAJC4/CD99/GINM1/PFKP/KLHL23/CCDC13/C9orf163/ABCD1/HEATR1/VARS2/CERS6-AS1/SMDT1/RAD23A/LOC105373690/REN/RSRC1/RAB9B/LOC105371058/MIB1/ATF6/COSMOC/PHOSPHO2-KLHL23/ARL3/NALCN-AS1/ADAMTS13/LOC107985258/TRAPPC2L/TMEM92-AS1/ZNF664/DPP9-AS1/VMA21/ZNF133/LOC107987256/DGKH/WASHC3/ATRAID/NDUFB1/ZNF765/CERK/HPCA/HEG1/TMEM234/MRPL21/DOK4/LOC105369391/FERMT3/GPI/MUC12/THAP9/LOC105376483/STAM-DT/RABAC1/LYG2/UQCC2/SELENOW/LOC107985599/STUB1-DT/HINT1/GPAT4/TMEM161A/MDN1/CYP1B1-AS1/EBLN3P/DDHD1/LY6S/ACTG1/KANTR/SLC10A3/DAZAP2/CYP2D6/ZFYVE19/BCL7B/TLE5/LINC01356/GGT2P/POTEM/LOC101928816/ZNF431/FAM131C/TMEM106C/ASIC4/TMCO3/SUCLG2/PHKG1/LOC107984833/MRPL53/LOC100289230/GEMIN8P4/RRAS/MIR181A1HG/TMEM107/MORN3/AP3B2/ZNF621/ST6GALNAC6/LOC105374868/LOC105376577/LOC107987290/POLR2I/CHMP2A/LOC107984745/LINC01270/C1QTNF9B/LOC105373950/SIX4/CDKN2B-AS1/TEX56P/PSMB7/KATNAL2/LGALS1/CAPNS1/RFT1/MRPL23/PGA5/CCDC61/NT5C3B/LOC105369915/OR5K2/POTEJ/SREBF2/SH3BP5-AS1/DCAF13/TMEM98/ADCY10P1/PSMB4/SCGB2B2/CCDC124/PCID2/MRPL54/SPAST/TBCB/RNASE4/PRKDC/WFDC10B/SCPEP1/ZEB1-AS1/LOC105369180/LOC105369549/PSMA2/ALDH9A1/EEF1AKMT1/LOC102724197/DAP3/SHANK3/CCS/LOC105378541/UBXN6/ZNF419/PPP1R35/HIBADH/ATP5F1C/C17orf113/NRSN2/NEPRO/ACTR1B/FOXD4L5/LOC105376146/ALG11/CDC42-AS1/CISD3/MIR3064/LANCL1-AS1/MPND/POTEI/CTAGE11P/TMEM256/SMYD5/NDUFAB1/MIEN1/LMAN2/POTEE/SLMO2-ATP5E/CERT1/MRPL41/SPDYE1/LOC105372726/LENG1/LOC105373948/CRELD2/ANG/FAM174C/UXT/NDUFS7/LOC105376392/SCARNA22/PCOTH/LOC105373386/PWAR1/OIP5-AS1/ST3GAL3/KIAA2013/ZNF283/SNORD138/GLRX3/CD14/NINJ2/C2orf49-DT/LOC107986163/PPP1R35-AS1/ALX4/NAF1/LYPD5/CDKL3/EIF3G/WFIKKN1/PDCD11/RPL37A/LOC107985656/SNCG/LINC02021/THRA/LOC414300/RHOC/DOC2A/ARL6IP4/OAZ1/ANAPC15/LOC729732/ZNF124/RNU12/MANEAL/ZNHIT2/DNASE2/LOC107986406/LOC101928548/KLHL11/LRRC23/SLC25A25/VTI1B/NFU1/ANTKMT/LOC101929280/LOC102724273/LOC101929255/LINC02175/LOC112268050/CTAGE7P/TUBG1/ZNF776/SZRD1/PSMD13/NDUFS6/VSIG1/RHOA/CCDC6/MYDGF/FBXO38/POTEG/ZDHHC1/CEBPA-DT/EMC6/CMTM4/RBX1/UTP14C/MOV10/CHID1/REX1BD/PRDM6-AS1/ARHGDIA/USF2/EXOC3-AS1/SAT2/BBLN/VPS25/LOC105369199/COPS6/LINC01719/PSMD2/ZCCHC18/ZNF485/CCNC/PTOV1-AS2/C1QTNF2/TIMMDC1/LOC101928453/APEH/LOC101928234/PTGES2/C1orf122/MIER2/MCCC2/TWF2/CCDC167/ENDOG/TMED1/MED16/NME2/FUCA2/LINC02589/CDH26/OLA1/LOC105374843/ANXA7/AP4M1/ACOT9/MRPS15/MRPS34/LOC102724823/LSM7/FIS1/KRTCAP2/HCFC1-AS1/LOC105374378/RPS6KA4/GOLGA2/TMEM44-AS1/SMIM7/PSMD8/MIR1282/NDUFA13/NDUFAF3/ALG9/MPG/PIGW/NME1-NME2/KLC1/TMEM223/FER1L5/POFUT1/RBM42/PHF1/HMG20B/DDX59/CSK/BFAR/PFN1/GHITM/LOC105369961/LACAT1/MPP3/MIR186/CCDC30/FAM50A/LOC107986982/DCTN2/POLR2J/SWSAP1/NKRF/LAMTOR2/YAP1/ZNF649-AS1/CATSPERD/KLC2/ATP5F1E/GGT3P/LINC02193/ARL2/C1QTNF9/CD81/MDN1-AS1/TCTN1/BLOC1S1/CLIC1/MRPL34/SPART-AS1/COA8/GTF2A1/SRRM1/MRPS25/PSMC3/TMCO6/SELENOH/BCLAF3/ACTG1P20/TGIF2-RAB5IF/VDAC3/PSMA7/HGS/GET3/NCBP1/PET100/DGCR6L/ARPC2/KCNRG/ARL8B/ARFGEF2/TMEM64/SOD2-OT1/LOC102723348/HDAC3/INCA1/NDUFB7/TOR1A/AMY1A/OVCA2/LRTOMT/CYFIP1/SMIM20/PSMB2/FAM161B/LIX1L-AS1/PTOV1/RPS19BP1/TOMM20L/TSR3/PDZD11/CFL1/EIF4E2/SENP5/PCGF1/IBA57/SSNA1/DKKL1/NR1H2/LOC102724808/MSH4/MED18/CEL/RPS19/SH3GLB2/SLC25A38/SH3BGRL3/CRIPT/AMY1C/PIH1D2/UBAC1/HEATR4/AMY1B/PLOD3/CCM2/PRORSD1P/ANAPC11/SELENOO/ARPC4/PSMB1/ALG14/FAM3A/RNPEP/KCTD21/ACER3/PTRHD1/TUSC3/ZNF169/PARK7/PAK2/LSM14B/SNX32/MLX/MVB12A/ITGB1BP1/FKBP8/SHARPIN/ADSL/FAAP20/UBE2K/EFCAB10/LOC101929937/NAT14/CUEDC2/GADD45GIP1/METTL22/TOMM6/CHMP3-AS1/LOC105377691/AK2/HECTD3/ZDHHC16/MRPL51/MIR103A2/GNB2/PACSIN2/KCNJ13/CAPN1/DUS1L/BAD/TMEM147/TMUB1/MIR4657/MOSPD3/LOC107986372/LOC107986373/NTMT1/PRNCR1/SCARNA15/LOC105373796/SMARCC1/MRPS7/GGTLC1/DMAC2/KLC2-AS1/MLF1-DT/DHX33/LOC107985227/ZNF529/NDUFS3/ZKSCAN1/SREBF2-AS1/GMCL2/SH2D7/RNF40/EXOC3/NUFIP1/UXT-AS1/SNX22/TRMT112/TMEM203/BAG6/NCLN/PPP1CA/NMT1/NDUFAF2/XRCC6/PSMC2/ARGLU1/UBE2M/KCTD21-AS1/P3H4/MRPL24/CYP3A5/PPIB/KBTBD4/RFXANK/SERF2/NENF/TMED10/LOC100505728/WDR83OS/EIPR1/PIPSL/CHMP4A/DNAJB12/PUF60/CYB5B/PPP2R5E/UROD/GANAB/IDH3B/APTX/NDUFC2-KCTD14/HYPK/DDX41/MIR6877/TMED10P1/WDR83/NUBP2/SERF2-C15ORF63/FNTB/MED28/MRPL16/PSMD9/FBXW5/FKBP10/STT3A/SNX17/LOC105371207/ZNF426/SEC13/DDOST/SURF6/SMG1P6/MRFAP1/ARHGEF4/RAB11B/BLOC1S4/CHURC1-FNTB/MORC2/INTS11/TCF25/PDAP1/PTCD1/SRSF6/MRPS11/SETD4/HERC2P4/PSMC5* |

**Table S8 Correlation between hub genes of co-expression module and DOC2A**

| Gene | Correlation | AbsCor | Pvalue | FDR | Total_kWithin |
| --- | --- | --- | --- | --- | --- |
| Neuron | | | | | |
| *PSD2* | 0.6927753 | 0.6927753 | 9.13E-23 | 1.42E-21 | 591.7245 |
| *KCNJ9* | 0.6696138 | 0.6696138 | 7.38E-21 | 7.83E-20 | 575.5325 |
| *EVI5L* | 0.6730583 | 0.6730583 | 3.94E-21 | 4.34E-20 | 574.4138 |
| *DISP2* | 0.6654415 | 0.6654415 | 1.56E-20 | 1.58E-19 | 573.9285 |
| *STX1B* | 0.7486367 | 0.7486367 | 3.32E-28 | 2.11E-26 | 567.2839 |
| *STXBP1* | 0.6743772 | 0.6743772 | 3.09E-21 | 3.51E-20 | 564.9872 |
| *MAP3K10* | 0.7604657 | 0.7604657 | 1.52E-29 | 1.49E-27 | 563.8269 |
| *CDK5R2* | 0.6948087 | 0.6948087 | 6.09E-23 | 9.74E-22 | 558.7802 |
| *KIF3C* | 0.7059773 | 0.7059773 | 6.17E-24 | 1.21E-22 | 552.8951 |
| *CTIF* | 0.6594729 | 0.6594729 | 4.47E-20 | 4.11E-19 | 551.7829 |
| Astrocyte | | | | | |
| *TRMT112* | 0.4178007 | 0.4178007 | 0.015547842 | 0.04929517 | 57.22761 |
| *ZNF283* | -0.3211544 | 0.3211544 | 0.068398698 | 0.13902183 | 55.83649 |
| *CHID1* | 0.4908206 | 0.4908206 | 0.003729681 | 0.01860809 | 53.9563 |
| *FAM50A* | 0.3750757 | 0.3750757 | 0.031493388 | 0.08007823 | 51.78239 |
| *OAZ1* | 0.3970319 | 0.3970319 | 0.02215044 | 0.06342553 | 50.60427 |
| *GPSM3* | 0.4453111 | 0.4453111 | 0.00940557 | 0.03472536 | 49.84032 |
| *FKBP8* | 0.4692006 | 0.4692006 | 0.005877454 | 0.02571038 | 49.36957 |
| *MYDGF* | 0.382285 | 0.382285 | 0.028125042 | 0.07374834 | 48.97301 |
| *SH3BGRL3* | 0.3828015 | 0.3828015 | 0.027895498 | 0.07356441 | 48.64083 |
| *CD81* | 0.4425585 | 0.4425585 | 0.009908677 | 0.03558641 | 48.44948 |

**Table S9 KEGG enrichment results in neuron dataset**

| category | subcategory | ID | Description | zScore | pvalue | p.adjust | qvalue | geneID | Count |
| --- | --- | --- | --- | --- | --- | --- | --- | --- | --- |
| Organismal Systems | Nervous system | hsa04724 | Glutamatergic synapse | 9.251698353 | 1.12E-15 | 3.86E-13 | 3.19E-13 | *GRIN3A/GRM1/KCNJ3/GRIN1/GRM7/SLC17A8/GRM5/GRM4/ADCY8/SLC17A7/CACNA1D/GRIK1/SLC17A6/GRIA2/GRIK3/CACNA1C/GRIN2A/PLA2G4C/GRIK2/SLC1A1/PLCB2/GRM8/CACNA1A/GRIN2D/GRIA3/GNG8/ADCY1/SHANK1/SHANK2/GRIN2C/GLS2/ADCY5/SLC1A2/GRIK4/ADCY2/GNG3/GRIK5/PRKCG/LZTS3/PPP3CC/GNG7/GNB5/SLC38A3/GNAO1/GNAI1/NCDN/GNG2/DLG4/GNG4/PLD2/GNG5/PPP3CB/PRKACA/PPP3R2/GRK2/ADCY6/MAPK3* | 57 |
| Organismal Systems | Nervous system | hsa04727 | GABAergic synapse | 8.449860617 | 3.21E-13 | 5.51E-11 | 4.54E-11 | *GABRB1/GABRA2/GABRG3/GABRG2/GABRA3/ADCY8/GABRA5/CACNA1D/GABBR2/GAD1/PLCL1/SLC12A5/GABRA1/CACNA1C/SLC6A1/GABRQ/CACNA1A/GNG8/ADCY1/CACNA1B/GLS2/ADCY5/ADCY2/GNG3/PRKCG/GNG7/GABRG1/GNB5/GABRA6/GABRA4/GABRB3/SLC38A3/GNAO1/GNAI1/GABARAP/GNG2/NSF/GNG4/GNG5/GABARAPL1/PRKACA/SRC/ABAT/ADCY6/GABARAPL2* | 45 |
| Human Diseases | Substance dependence | hsa05032 | Morphine addiction | 7.981697335 | 4.30E-12 | 3.78E-10 | 3.12E-10 | *GABRB1/GABRA2/KCNJ3/GABRG3/GABRG2/OPRM1/GABRA3/ADCY8/GABRA5/KCNJ9/PDE4C/GABBR2/PDE11A/GABRA1/GABRQ/CACNA1A/PDE8B/GNG8/ADCY1/CACNA1B/PDE4A/ADCY5/ADCY2/GNG3/PDE2A/PRKCG/GNG7/PDE4D/PDE3A/GABRG1/GNB5/GABRA6/GABRA4/GABRB3/GRK4/GNAO1/GNAI1/GRK5/GNG2/GNG4/GNG5/PRKACA/GRK2/ADCY6* | 44 |
| Human Diseases | Substance dependence | hsa05033 | Nicotine addiction | 8.328670255 | 4.41E-12 | 3.78E-10 | 3.12E-10 | *CHRNA6/GABRB1/GABRA2/GRIN3A/GRIN1/GABRG3/SLC17A8/GABRG2/GABRA3/SLC17A7/GABRA5/SLC17A6/GRIA2/GABRA1/GRIN2A/GABRQ/CACNA1A/GRIN2D/GRIA3/CACNA1B/GRIN2C/CHRNA4/CHRNA7/GABRG1/GABRA6/GABRA4/GABRB3* | 27 |
| Organismal Systems | Environmental adaptation | hsa04713 | Circadian entrainment | 6.369828308 | 1.42E-08 | 9.71E-07 | 8.02E-07 | *KCNJ3/GRIN1/RYR2/ADCYAP1/CACNA1I/CAMK2B/ADCY8/CACNA1D/KCNJ9/GRIA2/CACNA1C/GRIN2A/GUCY1A1/PLCB2/GRIN2D/GRIA3/GNG8/ADCY1/GRIN2C/ADCY5/CAMK2A/ADCY2/GNG3/RASD1/PRKCG/GUCY1B1/GNG7/NOS1AP/GNB5/GNAO1/GNAI1/GNG2/GNG4/GNG5/CAMK2G/PRKACA/ADCY6/MAPK3/CALM1/CALM3* | 40 |
| Organismal Systems | Development and regeneration | hsa04360 | Axon guidance | 6.068872422 | 2.31E-08 | 1.32E-06 | 1.09E-06 | *ABLIM3/PAK6/PLXNA4/EPHA5/PLXNB3/CAMK2B/EPHA6/UNC5A/PAK5/NGEF/NTNG1/UNC5D/NTNG2/TRPC6/SSH3/SEMA4A/LRRC4C/EFNA1/ROBO2/CAMK2A/ABLIM2/PIK3CD/SEMA6D/EPHB4/EPHA1/PAK3/PPP3CC/PARD6A/TRPC5/SEMA4G/PLXNA2/EFNA3/L1CAM/DCC/EPHB1/PRKCZ/GNAI1/SSH2/SLIT1/ROBO1/SEMA6B/CXCR4/SEMA4F/SMO/PLXNA3/PPP3CB/ITGB1/SEMA6C/PAK4/RND1/EFNB3/CAMK2G/RAC3/PIK3R3/LIMK1/SRC/PPP3R2/NCK2/MAPK3/PLXNB2/ABL1/CDC42* | 62 |
| Organismal Systems | Nervous system | hsa04723 | Retrograde endocannabinoid signaling | 6.063713962 | 3.14E-08 | 1.54E-06 | 1.27E-06 | *GABRB1/GABRA2/GRM1/KCNJ3/GABRG3/SLC17A8/GRM5/GABRG2/GABRA3/ADCY8/SLC17A7/RIMS1/GABRA5/CACNA1D/KCNJ9/SLC17A6/GRIA2/NDUFA4L2/GABRA1/CACNA1C/PLCB2/GABRQ/CACNA1A/GRIA3/GNG8/ADCY1/CACNA1B/CNR1/ADCY5/ADCY2/GNG3/FAAH/DAGLA/PRKCG/GNG7/NDUFA7/ABHD6/GABRG1/GNB5/GABRA6/GABRA4/GABRB3/GNAO1/GNAI1/GNG2/GNG4/GNG5/MAPK8/PRKACA/NDUFA9/ADCY6/MAPK3/NDUFA10* | 53 |
| Environmental Information Processing | Signaling molecules and interaction | hsa04080 | Neuroactive ligand-receptor interaction | 5.78166931 | 4.07E-08 | 1.74E-06 | 1.44E-06 | *CHRNA6/GABRB1/GABRA2/GRIN3A/GRM1/LPAR6/ADRA1A/APELA/GRIN1/CHRM2/MC4R/GRM7/GLRA3/CHRNB3/GABRG3/GRM5/GABRG2/ADCYAP1/GPR83/GAL/NTS/TRH/OPRM1/NTSR1/HCRTR2/GABRA3/CHRM4/GRM4/P2RX7/GLRA2/APLN/GABRA5/SSTR1/NPY5R/CHRM3/GRID2/GABBR2/GRIK1/GRIA2/MLN/VIPR1/GRIK3/PENK/P2RX5/GABRA1/NPY1R/GRIN2A/GRID1/TAC1/GRIK2/TSHR/SSTR2/GABRQ/GRM8/GHRHR/C5AR1/OPRL1/HCRTR1/GRIN2D/UTS2B/UTS2/GRIA3/CCK/LYNX1/HTR2A/CHRM5/TACR2/GPR156/GRIN2C/RLN1/GLRB/CNR1/HTR1E/GPR35/INSL5/GRIK4/CHRNA4/PAQR9/CHRNA7/MCHR1/GIPR/P2RX6/NMBR/CORT/CGA/MC1R/NPFFR2/GRIK5/UCN/PTGER2/THRA/GABRG1/MLNR/CYSLTR2/GABRA6/GABRA4/LYPD6/GABRB3/HTR5A/RXFP2/PLG/P2RY11/NPFFR1/OGFR* | 104 |
| Environmental Information Processing | Signal transduction | hsa04024 | cAMP signaling pathway | 5.640119744 | 1.35E-07 | 5.16E-06 | 4.26E-06 | *GRIN3A/PPP1R1B/GRIN1/CHRM2/ATP2B3/RYR2/ADCYAP1/ATP2B2/CAMK2B/ADCY8/CACNA1D/SSTR1/PDE4C/GABBR2/GLI1/GRIA2/CNGB1/BDNF/NPY1R/CACNA1C/GRIN2A/TSHR/SSTR2/FXYD2/CREB3L1/GRIN2D/GRIA3/HCN2/TNNI3/ADCY1/GRIN2C/VAV3/PDE4A/NFATC1/ADCY5/CNGA3/HTR1E/CAMK2A/PIK3CD/ADCY2/GIPR/CGA/PTGER2/SOX9/ATP1A3/PDE4D/POPDC2/ATP1B2/PDE3A/ATP1B1/ORAI1/GNAI1/ACOX1/VAV1/ATP2B4/PLD2/HCAR2/MAPK8/CAMK2G/ACOX3/RAC3/PIK3R3/SLC9A1/PRKACA/PLN/ADCY6/MAPK3/CALM1/CALM3/ATP1B3* | 70 |
| Environmental Information Processing | Signal transduction | hsa04020 | Calcium signaling pathway | 5.36738221 | 4.15E-07 | 1.42E-05 | 1.18E-05 | *GRIN3A/GRM1/ADRA1A/GRIN1/CHRM2/ATP2B3/GRM5/RYR2/CACNA1I/NTSR1/ATP2B2/CAMK2B/P2RX7/CACNA1E/ADCY8/CACNA1D/CHRM3/KDR/P2RX5/CACNA1C/FGF19/GRIN2A/SLC8A3/PLCB2/CACNA1A/GRIN2D/MST1R/HTR2A/ADCY1/SLC8A2/SLC8A1/CHRM5/MYLK2/TACR2/CACNA1B/TGFA/GRIN2C/NFATC1/MYLK/FGF18/PHKA1/CAMK2A/CHRNA7/ADCY2/FGF22/P2RX6/GNAL/NTRK3/PRKCG/NTRK2/PPP3CC/CAMK1D/CYSLTR2/MCOLN1/HTR5A/ORAI1/FGF20/SLC25A4/STIM1/PTK2B/CXCR4/ATP2B4/ORAI2/PPP3CB/MCOLN3/ERBB2/CAMK2G/GNA11/FGFR1/PRKACA/PLN/PHKB/PPP3R2/CALM1/CALM3* | 75 |
| Organismal Systems | Nervous system | hsa04725 | Cholinergic synapse | 5.27471276 | 1.37E-06 | 4.27E-05 | 3.53E-05 | *CHRNA6/KCNJ3/CHRM2/CHRM4/CAMK2B/KCNQ3/SLC18A3/ADCY8/CACNA1D/CHRM3/CACNA1C/PLCB2/CACNA1A/CREB3L1/ACHE/GNG8/ADCY1/CHRM5/CACNA1B/ADCY5/CHRNA4/CAMK2A/PIK3CD/CHRNA7/ADCY2/GNG3/PRKCG/GNG7/GNB5/GNAO1/GNAI1/GNG2/GNG4/KCNQ2/GNG5/CAMK2G/GNA11/PIK3R3/PRKACA/ADCY6/MAPK3* | 41 |
| Organismal Systems | Endocrine system | hsa04911 | Insulin secretion | 5.28387007 | 1.83E-06 | 5.24E-05 | 4.32E-05 | *GCK/RYR2/ADCYAP1/CAMK2B/ADCY8/CACNA1D/CHRM3/CACNA1C/PLCB2/FXYD2/CREB3L1/CCK/ADCY1/ADCY5/CAMK2A/ADCY2/KCNJ11/KCNN3/RAB3A/STX1A/PRKCG/SNAP25/ATP1A3/TRPM4/ATP1B2/ATP1B1/VAMP2/KCNMB4/CAMK2G/GNA11/PRKACA/ADCY6/ATP1B3* | 33 |
| Organismal Systems | Nervous system | hsa04721 | Synaptic vesicle cycle | 5.268353783 | 2.14E-06 | 5.66E-05 | 4.67E-05 | *SLC17A8/UNC13C/SLC18A3/SLC17A7/DNM3/RIMS1/SLC17A6/SLC6A1/SLC1A1/CACNA1A/CACNA1B/SLC1A2/CPLX1/STX1B/UNC13A/RAB3A/SLC6A7/STX1A/STXBP1/SNAP25/SYT1/ATP6V1C2/CPLX2/VAMP2/ATP6V1A/ATP6V0E2/NSF/CLTB/ATP6V0A1/AP2A1/ATP6V1B2* | 31 |
| Organismal Systems | Nervous system | hsa04728 | Dopaminergic synapse | 4.779582998 | 8.70E-06 | 0.000213268 | 0.00017606 | *KCNJ3/PPP2R2C/PPP1R1B/CAMK2B/CACNA1D/KCNJ9/GRIA2/TH/MAOA/MAOB/CALY/CACNA1C/GRIN2A/DDC/PLCB2/CACNA1A/CREB3L1/GRIA3/GNG8/CACNA1B/ADCY5/CAMK2A/GNG3/GNAL/PRKCG/PPP3CC/GNG7/PPP2R5B/GNB5/KIF5A/GNAO1/GNAI1/GNG2/GNG4/GNG5/PPP3CB/MAPK8/CAMK2G/PPP2R3C/PRKACA/PPP2R2D/CALM1/CALM3* | 43 |
| Human Diseases | Substance dependence | hsa05031 | Amphetamine addiction | 4.896321308 | 1.02E-05 | 0.000222651 | 0.000183805 | *GRIN3A/PPP1R1B/GRIN1/CAMK2B/CACNA1D/GRIA2/TH/MAOA/MAOB/CACNA1C/GRIN2A/DDC/CREB3L1/GRIN2D/GRIA3/GRIN2C/ADCY5/CAMK2A/STX1A/PRKCG/PPP3CC/PPP3CB/CAMK2G/PRKACA/PPP3R2/CALM1/CALM3* | 27 |
| Organismal Systems | Circulatory system | hsa04261 | Adrenergic signaling in cardiomyocytes | 4.700839273 | 1.04E-05 | 0.000222651 | 0.000183805 | *PPP2R2C/ADRA1A/CACNG5/ATP2B3/RYR2/ATP2B2/CAMK2B/CACNG2/CACNA2D3/ADCY8/CACNA1D/CACNA1C/SLC8A3/PLCB2/CACNB2/FXYD2/CREB3L1/TNNI3/ADCY1/SLC8A2/SLC8A1/MYH7/ADCY5/CAMK2A/ADCY2/CACNA2D2/CACNB1/ATP1A3/POPDC2/PPP2R5B/ATP1B2/KCNE1/ATP1B1/GNAI1/ATP2B4/CAMK2G/PPP2R3C/SLC9A1/PRKACA/PLN/PPP2R2D/ADCY6/CACNG7/MAPK3/CALM1/CALM3/TPM3/ATP1B3* | 48 |
| Environmental Information Processing | Signal transduction | hsa04022 | cGMP-PKG signaling pathway | 4.312972848 | 4.22E-05 | 0.00085096 | 0.000702496 | *ADRA1A/ATP2B3/ATP2B2/ADCY8/CACNA1D/CNGB1/CACNA1C/GUCY1A1/MEF2C/SLC8A3/PLCB2/FXYD2/CREB3L1/ADCY1/SLC8A2/SLC8A1/MYLK2/TRPC6/MYH7/NFATC1/ADCY5/MYLK/ADCY2/NPR2/PDE2A/PRKCE/GUCY1B1/PPP3CC/ATP1A3/ATP1B2/PDE3A/ATP1B1/GNAI1/MEF2D/SLC25A4/KCNMB4/RGS2/ATP2B4/PPP3CB/SRF/GNA11/PLN/GNA12/PPP3R2/ADCY6/MAPK3/CALM1/CALM3/ATP1B3* | 49 |
| Organismal Systems | Endocrine system | hsa04925 | Aldosterone synthesis and secretion | 4.400589112 | 4.49E-05 | 0.000855017 | 0.000705845 | *KCNK9/NR4A2/ATP2B3/CACNA1I/ATP2B2/CAMK2B/ADCY8/CACNA1D/CACNA1C/KCNK3/PLCB2/CREB3L1/ADCY1/ADCY5/CAMK2A/ADCY2/PDE2A/DAGLA/PRKCG/PRKCE/CAMK1D/ATP1A3/ATP1B2/ATP1B1/ORAI1/ATP2B4/CAMK2G/GNA11/PRKACA/ADCY6/CALM1/CALM3/ATP1B3* | 33 |
| Organismal Systems | Endocrine system | hsa04921 | Oxytocin signaling pathway | 4.218123922 | 6.20E-05 | 0.001119922 | 0.000924533 | *KCNJ3/CACNG5/RYR2/CAMK2B/CACNG2/CACNA2D3/ADCY8/CACNA1D/KCNJ9/CACNA1C/PLA2G4C/GUCY1A1/MEF2C/PLCB2/CACNB2/ADCY1/MYLK2/NFATC1/ADCY5/MYLK/CAMK2A/ADCY2/NPR2/CACNA2D2/PRKCG/GUCY1B1/CACNB1/PPP3CC/CAMK1D/CCND1/PPP1R12B/GNAO1/GNAI1/RGS2/PPP3CB/MAP2K5/CAMK2G/PRKACA/SRC/MAPK7/PPP3R2/ADCY6/CACNG7/MAPK3/CALM1/CALM3* | 46 |
| Organismal Systems | Nervous system | hsa04720 | Long-term potentiation | 4.101150999 | 0.000163525 | 0.002804461 | 0.002315176 | *GRM1/GRIN1/GRM5/CAMK2B/ADCY8/GRIA2/CACNA1C/GRIN2A/PLCB2/GRIN2D/ADCY1/GRIN2C/CAMK2A/PRKCG/PPP3CC/RPS6KA2/PPP3CB/CAMK2G/PRKACA/PPP3R2/ARAF/MAPK3/CALM1/CALM3* | 24 |
| Organismal Systems | Digestive system | hsa04971 | Gastric acid secretion | 3.995300375 | 0.000210264 | 0.003434311 | 0.002835137 | *CAMK2B/ADCY8/CHRM3/SSTR2/PLCB2/ADCY1/MYLK2/ADCY5/MYLK/CAMK2A/ADCY2/PRKCG/ATP1A3/ATP1B2/ATP1B1/GNAI1/KCNJ1/CAMK2G/SLC9A1/PRKACA/EZR/ADCY6/CALM1/SLC9A4/CALM3/ATP1B3* | 26 |
| Human Diseases | Substance dependence | hsa05030 | Cocaine addiction | 4.055026515 | 0.000238414 | 0.003679464 | 0.00303752 | *GRIN3A/PPP1R1B/GRIN1/GRIA2/TH/MAOA/BDNF/MAOB/GRIN2A/DDC/RGS9/CREB3L1/GRIN2D/GRIN2C/ADCY5/CDK5R1/GNAI1/DLG4/PRKACA* | 19 |
| Cellular Processes | Transport and catabolism | hsa04144 | Endocytosis | 3.745788463 | 0.000246728 | 0.003679464 | 0.00303752 | *DNM3/IQSEC3/PSD2/WIPF3/DNAJC6/IQSEC2/SH3GL3/SH3GL2/IL2RB/LDLRAP1/EHD4/SNX32/HLA-E/ACAP1/WASHC1/AGAP2/PIP5KL1/EHD3/RAB11FIP4/PARD6A/VPS37D/PSD/AGAP1/SMAP2/GRK4/KIF5A/MVB12B/PRKCZ/NEDD4L/GRK5/CLTB/BIN1/VPS26B/CXCR4/SMAP1/CBLB/RAB11FIP5/PLD2/IQSEC1/PIP5K1C/ZFYVE27/EPN1/EPN2/RAB11B/AP2A1/GIT1/CHMP1B/SMAD3/SRC/WASHC3/GRK2/WIPF2/SH3GLB2/PML/ARPC5/CHMP7/RAB35/CDC42/CHMP1A/HGS/CHMP5/RAB4A/CYTH3/CHMP4B/RAB5B* | 65 |
| Organismal Systems | Nervous system | hsa04726 | Serotonergic synapse | 3.842094851 | 0.000266068 | 0.003802555 | 0.003139135 | *GABRB1/KCNJ3/KCND2/CACNA1D/KCNJ9/MAOA/MAOB/CACNA1C/PLA2G4C/DDC/CYP4X1/PLCB2/CACNA1A/TPH2/GNG8/HTR2A/CACNA1B/ADCY5/HTR1E/GNG3/CYP2D6/PRKCG/GNG7/GNB5/GABRB3/HTR5A/DUSP1/GNAO1/GNAI1/GNG2/GNG4/GNG5/PRKACA/ARAF/MAPK3* | 35 |
| Environmental Information Processing | Signal transduction | hsa04010 | MAPK signaling pathway | 3.570504514 | 0.000412507 | 0.005659593 | 0.004672182 | *RASGRF1/PTPRR/CACNG5/CACNA1I/CACNG2/CACNA1E/CACNA2D3/TRADD/CACNA1D/KDR/MAPT/BDNF/CACNA1C/DUSP8/FGF19/PLA2G4C/MEF2C/CACNB2/CACNA1A/ANGPT2/MAP3K5/RASGRP2/PTPN5/DUSP10/CACNA1B/TGFA/RASGRF2/NFATC1/EFNA1/FGF18/DUSP6/FGF22/TAOK3/MAP3K9/CACNA2D2/PRKCG/NTRK2/CACNB1/PPP3CC/MAPK8IP2/MAP3K10/PSPN/EFNA3/DUSP1/MRAS/DUSP7/FGF20/MAP3K13/NFKB2/MAPK8IP1/RPS6KA2/MAP3K12/PPP3CB/MAPK8/SRF/MAP2K5/ERBB2/MAP4K2/RAC3/FGFR1/PRKACA/MAPK7/GNA12/PPP3R2/MAPK8IP3/ARAF/LAMTOR3/MAP3K11/CACNG7/MAPK3/JUND/CDC42/TAB1/TRAF2* | 74 |
| Environmental Information Processing | Signal transduction | hsa04371 | Apelin signaling pathway | 3.654419153 | 0.000437014 | 0.005765219 | 0.00475938 | *APELA/RYR2/APLN/ADCY8/MEF2C/SLC8A3/PPARGC1A/PLCB2/GNG8/ADCY1/SLC8A2/SLC8A1/MYLK2/ADCY5/MYLK/MAP1LC3B2/ADCY2/GNG3/PRKCE/GNG7/PLIN1/GNB5/HDAC5/CCND1/MRAS/GNAI1/MEF2D/GNG2/GNG4/HDAC4/GNG5/SMAD3/NOTCH3/MAP1LC3B/SLC9A1/PRKACA/ADCY6/MAPK3/CALM1/CALM3* | 40 |
| Human Diseases | Cardiovascular disease | hsa05414 | Dilated cardiomyopathy | 3.41933784 | 0.001027361 | 0.01305129 | 0.010774274 | *CACNG5/RYR2/CACNG2/ITGA1/CACNA2D3/ADCY8/CACNA1D/LAMA2/SGCZ/CACNA1C/SLC8A3/CACNB2/ITGA10/TNNI3/ADCY1/SLC8A2/SLC8A1/MYH7/ADCY5/SGCG/ADCY2/CACNA2D2/SNTA1/CACNB1/ITGA5/ITGB1/PRKACA/PLN/ADCY6/CACNG7/TPM3* | 31 |
| Human Diseases | Endocrine and metabolic disease | hsa04930 | Type II diabetes mellitus | 3.494730914 | 0.001263979 | 0.015483738 | 0.01278234 | *GCK/CACNA1E/CACNA1D/CACNA1C/HK3/CACNA1A/CACNA1B/PIK3CD/SOCS1/KCNJ11/PRKCE/PRKCZ/HK1/MAPK8/PIK3R3/MAPK3/PKM* | 17 |
| Environmental Information Processing | Signal transduction | hsa04012 | ErbB signaling pathway | 3.266710019 | 0.001743419 | 0.020620433 | 0.017022852 | *PAK6/NRG2/CAMK2B/SHC4/PAK5/NRG3/TGFA/CAMK2A/PIK3CD/PRKCG/PAK3/NRG1/EIF4EBP1/CBLB/MAPK8/SHC2/PAK4/ERBB2/CAMK2G/PIK3R3/SRC/ARAF/STAT5B/NCK2/MAPK3/ABL1* | 26 |
| Organismal Systems | Excretory system | hsa04961 | Endocrine and other factor-regulated calcium reabsorption | 3.283692349 | 0.002101177 | 0.024023462 | 0.019832166 | *ATP2B3/ATP2B2/DNM3/SLC8A3/PLCB2/FXYD2/SLC8A2/SLC8A1/PRKCG/ATP1A3/ATP1B2/ATP1B1/CLTB/ATP2B4/AP2A1/PRKACA/ADCY6/ATP1B3* | 18 |
| Metabolism | Nucleotide metabolism | hsa00230 | Purine metabolism | 3.119699245 | 0.002234636 | 0.024725164 | 0.020411445 | *ENTPD2/ADCY8/PDE4C/PDE11A/ENTPD3/ENPP3/GUCY1A1/PDE8B/GDA/ADCY1/AK5/PDE4A/ADCY5/FHIT/AK8/ADCY2/NPR2/PDE2A/GUCY1B1/PGM2/PDE4D/PDE3A/AK9/NT5M/ALLC/PNP/AK1/APRT/ADA/HDDC3/ATIC/NME4/NT5C/ADCY6/PAPSS1* | 35 |
| Metabolism | Carbohydrate metabolism | hsa00520 | Amino sugar and nucleotide sugar metabolism | 3.251073056 | 0.002729131 | 0.02925287 | 0.024149216 | *GCK/HK3/FPGT/RENBP/PGM2/CHIT1/PMM2/GALK1/HK1/NAGK/GALK2/HEXA/UXS1/MPI* | 14 |
| Environmental Information Processing | Signal transduction | hsa04014 | Ras signaling pathway | 2.870007167 | 0.003681551 | 0.038265815 | 0.031589701 | *RASGRF1/KSR2/GRIN1/PAK6/PLA2G3/KDR/SHC4/PAK5/BDNF/FGF19/GRIN2A/PLA2G4C/RASSF5/RASA4/ANGPT2/RASGRP2/GNG8/PLA2G1B/TGFA/RASGRF2/EFNA1/FGF18/PIK3CD/FGF22/GNG3/PRKCG/NTRK2/PAK3/GNG7/PLA2G12A/GNB5/EFNA3/MRAS/GNG2/FGF20/GAB2/GNG4/KSR1/PLD2/GNG5/RGL2/BCL2L1/PLA2G6/MAPK8/SHC2/PAK4/RALGDS/RAC3/PIK3R3/FGFR1/PRKACA/MAPK3/ABL1/CALM1/CDC42/CALM3/RAB5B* | 57 |
| Organismal Systems | Circulatory system | hsa04260 | Cardiac muscle contraction | 2.912697254 | 0.004490227 | 0.045298466 | 0.037395388 | *CACNG5/RYR2/COX6B2/CACNG2/CACNA2D3/CACNA1D/CACNA1C/SLC8A3/CACNB2/FXYD2/TNNI3/SLC8A2/SLC8A1/SLC9A7/MYH7/CACNA2D2/CACNB1/ATP1A3/SLC9A6/ATP1B2/ATP1B1/SLC9A1/CACNG7/TPM3/ATP1B3* | 25 |
| Metabolism | Amino acid metabolism | hsa00350 | Tyrosine metabolism | 3.047127264 | 0.004678379 | 0.04584811 | 0.037849138 | *HPD/DCT/TYRP1/TH/MAOA/MAOB/DDC/DBH/AOC2/TPO/TAT/GSTZ1/GOT1* | 13 |
| Organismal Systems | Endocrine system | hsa04928 | Parathyroid hormone synthesis, secretion and action | 2.843594227 | 0.004878228 | 0.046478673 | 0.038369689 | *GATA3/NR4A2/ADCY8/PDE4C/MMP24/RXRG/MEF2C/PLCB2/CREB3L1/ADCY1/PDE4A/ADCY5/ADCY2/SGK1/PRKCG/WNK2/PDE4D/MMP17/GNAI1/MEF2D/PLD2/MMP15/RXRA/GNA11/FGFR1/PRKACA/GNA12/ARAF/ADCY6/MAPK3/JUND* | 31 |

**Table S10 GO enrichment results in neuron dataset**

| ONTOLOGY | ID | Description | zScore | pvalue | p.adjust | qvalue | geneID | Count |
| --- | --- | --- | --- | --- | --- | --- | --- | --- |
| CC | GO:0097060 | synaptic membrane | 14.62240049 | 1.30E-37 | 9.08E-35 | 7.49E-35 | *CHRNA6/PLPPR4/GABRB1/GABRA2/GRIN3A/GRM1/SCN2A/KCNJ3/GRIN1/CHRM2/CNTNAP4/GRM7/GLRA3/CHRNB3/GABRG3/CACNG5/APBA1/CDH9/KCNC2/CNTN5/ATP2B3/KCND2/SLITRK3/GRM5/DDN/GABRG2/CBLN1/LRRC7/UNC13C/ATP2B2/GABRA3/CHRM4/KCTD8/FOSL1/LRFN5/GLRA2/CACNG2/ADCY8/SORCS3/DNM3/RIMS1/GABRA5/KCNJ9/CHRM3/GRID2/GABBR2/SHC4/IQSEC3/CNTN1/GPR158/GRIK1/GRIA2/LRFN2/PPFIA2/GRIK3/COL13A1/CNTNAP2/GABRA1/GRIP2/CACNA1C/GRIN2A/SLC6A1/GRID1/NTNG1/GRIK2/SHISA8/RGS9/GABRQ/KCNA2/ANK1/CLSTN2/LRRTM3/LRRTM1/DNAJC6/NRXN1/SHISA7/SLITRK1/GRIN2D/GRIA3/LHFPL4/NTNG2/SNAP91/FAIM2/HTR2A/ADCY1/PTPRO/SHANK1/ADGRB3/DGKI/SHANK2/CHRM5/GRIN2C/ADAM11/GLRB/CNR1/LRRC4C/SLC1A2/PRRT1/FBXO2/LRRTM4/MAGEE1/KCNB1/GRIK4/CHRNA4/CHRNA7/ERC2/LIN7A/UNC13A/KCNJ11/LRFN1/SYT7/P2RX6/SYP/LIN7B/LRRTM2/PDE2A/NRXN2/SYDE1/DAGLA/SORCS2/SLC6A7/GRIK5/STX1A/PRKCG/SNTA1/TMEM240/STXBP1/NSG1/SNAP25/ANK2/RIMS4/ERC1/SYT1/ABHD6/GABRG1/PRR7/GPM6A/ITGA5/GABRA6/GABRA4/GABRB3/HTR5A/LRFN4/SNCAIP/DCC/ADGRL1/CSPG5/RIMS3/SLC4A8/CLTB/CLSTN3/DLG4/LRRC4B/HIP1R/ATP2B4/LRFN3/PPP1R9B/SEMA4F/CNIH2/PICK1/SYT11/ITGB1/PSEN2/ERBB2/EFNB3/FXYD6/ABHD17A/SRC/CLSTN1/CACNG7/RNF10/NLGN2/PTPRA/EXOC3* | 174 |
| CC | GO:0099572 | postsynaptic specialization | 13.02660319 | 2.25E-30 | 7.85E-28 | 6.47E-28 | *PLPPR4/GABRB1/GABRA2/GRIN3A/GRM1/GRIN1/CACNG5/KCND2/PAK6/SLITRK3/GRM5/LRRC7/ATP2B2/LRFN5/CACNG2/ADCY8/SORCS3/DNM3/GABRA5/GRID2/IQSEC3/GRIK1/GRIA2/PPFIA2/LYN/GRIK3/CPEB1/CPEB3/GRIP2/CACNA1C/GRIN2A/GRID1/GRIK2/SHISA8/RGS9/SLC8A3/CLSTN2/LRRTM3/LRRTM1/DNAJC6/RNF112/SHISA7/SLITRK1/GRIN2D/DRP2/GRIA3/LHFPL4/ADGRA1/SRCIN1/SYN1/DMTN/ADCY1/PTPRO/NEURL1/SHANK1/SLC8A2/DLGAP3/CABP1/ADGRB3/SLC8A1/DGKI/SHANK2/GRIN2C/SH3GL3/GLRB/LRRC4C/TMEM108/PRRT1/DCLK1/PRKN/ARHGAP44/ANKS1B/KCNB1/CPEB4/GRIK4/CAMK2A/LIN7A/LRFN1/P2RX6/GNG3/MAGI2/LIN7B/LRRTM2/ZDHHC15/DAGLA/SORCS2/SH2D5/GRIK5/PRNP/KALRN/PRKCG/RTN1/NTRK2/LZTS3/CDK5R1/SAMD14/PAK3/PSD/MAPK8IP2/NCS1/GAP43/PRR7/GABRA4/HTR5A/ADD2/LRFN4/DCC/PRKCZ/MPP2/DTNB/ARHGEF9/NSF/CLSTN3/DLG4/LRRC4B/NSMF/PTK2B/CTNND2/HIP1R/ABI3/LRFN3/PPP1R9B/SEMA4F/CADM1/IQSEC1/CNIH2/PICK1/SYT11/PALM/EFNB3/RTN4/GIT1/ABHD17A/RTN3/SRC/DVL1/MINK1/CLSTN1/TSC2/NCK2/CACNG7/ADD1/RNF10/PCBP1/NLGN2/DBNL* | 146 |
| CC | GO:0098984 | neuron to neuron synapse | 12.51664994 | 1.72E-28 | 4.00E-26 | 3.30E-26 | *PLPPR4/GRIN3A/GRM1/GRIN1/CHRM2/GRM7/CACNG5/PAK6/SLITRK3/GRM5/PLXNA4/LRRC7/NTSR1/ATP2B2/LRFN5/CACNG2/ADCY8/SORCS3/DNM3/GRID2/IQSEC3/GRIK1/GRIA2/GRIK3/CPEB1/PENK/CPEB3/GRIP2/CACNA1C/GRIN2A/GRID1/GRIK2/SLC1A1/SHISA8/RGS9/SLC8A3/CLSTN2/LRRTM3/DNAJC6/RNF112/SHISA7/SLITRK1/GRIN2D/DRP2/GRIA3/ADGRA1/SRCIN1/SYN1/DMTN/ADCY1/PTPRO/NEURL1/SHANK1/SLC8A2/DLGAP3/CABP1/ADGRB3/C1QL1/SLC8A1/DGKI/SHANK2/GRIN2C/SH3GL3/LRRC4C/TMEM108/PRRT1/DCLK1/PRKN/ARHGAP44/ANKS1B/CPEB4/GRIK4/CAMK2A/LIN7A/LRFN1/SYT7/GNG3/MAGI2/LIN7B/LRRTM2/PRKAR1B/ZDHHC15/DAGLA/SORCS2/SH2D5/GRIK5/PRNP/KALRN/PRKCG/RTN1/NTRK2/LZTS3/CDK5R1/SAMD14/PAK3/PSD/ATP1A3/SYT1/MAPK8IP2/NCS1/ATP1B2/GAP43/PRR7/ADD2/LRFN4/DCC/PRKCZ/SLC4A8/MPP2/DTNB/ARHGEF9/NSF/CLSTN3/DLG4/LRRC4B/NSMF/PTK2B/CTNND2/HIP1R/ABI3/LRFN3/PPP1R9B/SEMA4F/CADM1/IQSEC1/CNIH2/ROGDI/PICK1/SYT11/ITGB1/PALM/EFNB3/RTN4/GIT1/ABHD17A/SORT1/RTN3/DVL1/MINK1/CLSTN1/TSC2/NCK2/CACNG7/ADD1/RNF10/PCBP1/NLGN2/DBNL* | 148 |
| BP | GO:0050804 | modulation of chemical synaptic transmission | 12.60679011 | 1.30E-29 | 5.31E-26 | 4.71E-26 | *RASGRF1/CHRNA6/SLC4A10/PLPPR4/SCGN/GRIN3A/GRM1/ADRA1A/GRIN1/CHRM2/CNTNAP4/GRM7/CHRNB3/CACNG5/APBA1/SLC24A2/GRM5/CBLN1/TNR/NALCN/UNC13C/CAMK2B/KCNQ3/GRM4/CACNG2/CALB2/SLC18A3/ADCY8/SORCS3/RIMS1/NPY5R/SV2C/GRID2/GPR158/GRIK1/GRIA2/ACE/MAPT/LAMA2/LRFN2/PPFIA2/PLCL1/GRIK3/CBLN2/CPEB3/BDNF/GRIN2A/SLC6A1/GRID1/GUCY1A1/NTNG1/MEF2C/GRIK2/SLC1A1/CELF4/SHISA8/SLC8A3/FRRS1L/ROR2/GRM8/CLSTN2/CACNA1A/LRRTM1/NRXN1/RELN/SHISA7/GRIN2D/GRIA3/ACHE/NTNG2/SYN1/HTR2A/NRG3/ADCY1/NEURL1/SHANK1/SLC8A2/DLGAP3/DGKI/SHANK2/TACR2/APOE/CACNA1B/IQSEC2/GRIN2C/RASGRF2/JPH3/CNR1/BEGAIN/LRRC4C/SNCA/TMEM108/PRRT1/FBXO2/PRKN/ARHGAP44/CPLX1/KCNB1/GRIK4/CAMK2A/CHRNA7/ERC2/STX1B/UNC13A/SYT7/SYP/SYT4/LRRTM2/PRKAR1B/PRKAR2B/RAB3A/SORCS2/CACNA2D2/GRIK5/STX1A/PRNP/OPHN1/JPH4/KCTD13/PRKCG/CLN3/PXK/STXBP1/NSG1/SNAP25/PPFIA3/PRKCE/NTRK2/TBC1D24/INA/RIMS4/GUCY1B1/UCN/ERC1/SYT1/ABHD6/MAPK8IP2/SYBU/PINK1/BAIAP3/BTBD9/BRSK1/SNCAIP/DCC/ASIC1/EPHB1/PRKCZ/SYNGR1/CSPG5/CPLX2/VAMP2/RIMS3/CYFIP1/SLC4A8/NCDN/MPP2/SERPINE2/CLSTN3/DLG4/NSMF/PTK2B/KCNMB4/PLG/TMEM25/PICK1/PPP3CB/SYT11/ITGB1/SRF/FBXL20/DGKZ/EFNB3/PFN1/RTN4/GIT1/TUBB2B/PRKACA/DVL1/VPS18/CLSTN1/CACNG7/MAPK3/ABL1/NLGN2/PTPRA* | 185 |
| BP | GO:0050808 | synapse organization | 11.50428009 | 2.73E-25 | 5.55E-22 | 4.93E-22 | *DSCAM/GABRA2/PCDHB5/SLITRK6/ICAM5/GABRG3/SNCB/CDH9/CNTN5/ZNF804A/SLITRK3/GRM5/GABRG2/PLXNA4/CBLN1/TNR/LINGO2/UNC13C/IL10RA/GABRA3/CAMK2B/LRFN5/CACNG2/SLC18A3/DNM3/GABRA5/PPFIA4/NEGR1/GRID2/GPR158/MAPT/LRFN2/PPFIA2/SPTBN4/NGEF/CBLN2/ZNF365/BDNF/GABRA1/SLC6A1/GRID1/NTNG1/MEF2C/SLC1A1/SLC8A3/FRRS1L/CACNB2/ROR2/CLSTN2/PCDHB6/LRRTM3/C5AR1/LRRTM1/NRXN1/RELN/SHISA7/SLITRK1/PCDHB9/DRP2/LHFPL4/ACHE/NTNG2/PCDHB16/SRCIN1/SYN1/NRG3/SEZ6L/PTPRO/NEURL1/SHANK1/SLC8A2/DLGAP3/ADGRB3/C1QL1/SHANK2/APOE/SPOCK2/KIRREL3/FZD9/SEMA4A/GLRB/LRRC4C/EFNA1/SNCA/FRMPD4/TMEM108/PRRT1/ROBO2/ARHGAP44/LGI2/NPTX1/CHRNA7/ERC2/RAB29/PCDHB10/UNC13A/LRFN1/PCDHB14/RAB17/LRRTM2/NRXN2/RAB3A/ZDHHC15/CACNA2D2/PRNP/OPHN1/CLN3/SNTA1/SEZ6L2/SNAP25/PPFIA3/NTRK2/LZTS3/INA/CDK5R1/PAK3/CACNB1/THBS2/DNER/MDGA1/ERC1/NOS1AP/AMIGO1/GAP43/SYBU/GABRG1/GPM6A/GABRA6/GABRA4/GABRB3/L1CAM/ADD2/KIF1A/LRFN4/EPHB1/ADGRL1/CYFIP1/CTNNA2/PCDHB2/SDK1/ARHGEF9/CNTNAP1/CLSTN3/SLIT1/ARHGAP39/DLG4/LRRC4B/PTK2B/CTNND2/HIP1R/ABI3/LRFN3/DAB2IP/APBB2/PICK1/ITGB1/PALM/ERBB2/ARMCX5-GPRASP2/EFNB3/PFN1/ACTN1/RAC3/ABHD17A/SORT1/DVL1/ADGRF1/EZR/CLSTN1/MESD/PLXNB2/ABL1/TUBA1A/CDC42/NLGN2/DBNL* | 176 |
| CC | GO:0098978 | glutamatergic synapse | 9.991361193 | 1.31E-19 | 1.01E-17 | 8.35E-18 | *SLC6A17/PLPPR4/GRIN3A/GRM1/SCN2A/ABLIM3/CHRM2/ICAM5/CACNG5/APBA1/ATP2B3/KCND2/GRM5/PLXNA4/CBLN1/TNR/ATP2B2/LRFN5/CACNG2/ADCY8/SORCS3/DNM3/GRID2/PPFIA2/LYN/GRIK3/CBLN2/GRIP2/CALY/PSD2/GRIN2A/GRID1/HPCA/GUCY1A1/NTNG1/GRIK2/RGS9/ROR2/KCNA2/CLSTN2/LRRTM3/LRRTM1/SLITRK1/GRIN2D/DRP2/NTNG2/ADGRA1/SRCIN1/HTR2A/NRG3/ADCY1/PTPRO/SHANK1/DLGAP3/ADGRB3/C1QL1/SHANK2/APOE/GRIN2C/SH3GL3/SH3GL2/FZD9/CNR1/LRRC4C/NPTXR/SLC1A2/FBXO2/ARHGAP44/CPLX1/NPTX1/ERC2/KCNJ11/P2RX6/SYT4/RAB17/LRRTM2/PRKAR1B/NRXN2/PRKAR2B/CADPS/NAPB/OPHN1/STXBP1/NSG1/DOC2A/SNAP25/PPFIA3/ELAVL4/GUCY1B1/PAK3/PPP3CC/NOS1AP/SYT1/TSPOAP1/ABHD6/PRR7/GPM6A/ITGA5/BTBD9/SV2A/LRFN4/EPHB1/PRKCZ/CSPG5/CPLX2/SLC4A8/CNTNAP1/CLSTN3/BIN1/ARHGAP39/DLG4/LRRC4B/PTK2B/HIP1R/PLG/PORCN/ATP2B4/LRFN3/DGKQ/SEMA4F/PPP3CB/ITGB1/FBXL20/EFNB3/PFN1/RTN4/FXYD6/ACTN1/CPT1C/RAC3/ABHD17A/SORT1/PRKACA/SRC/DVL1/VPS18/CLSTN1/CACNG7/MAPK3/RNF10/AP3D1/CDC42/DBNL* | 143 |
| BP | GO:0007416 | synapse assembly | 8.864686737 | 3.09E-15 | 2.09E-12 | 1.85E-12 | *DSCAM/GABRA2/PCDHB5/SLITRK6/ICAM5/GABRG3/CDH9/CNTN5/SLITRK3/GABRG2/CBLN1/LINGO2/GABRA3/LRFN5/DNM3/GABRA5/NEGR1/GRID2/CBLN2/BDNF/GABRA1/MEF2C/CLSTN2/PCDHB6/LRRTM3/LRRTM1/NRXN1/SLITRK1/PCDHB9/LHFPL4/ACHE/NTNG2/PCDHB16/NRG3/ADGRB3/SHANK2/SPOCK2/KIRREL3/SEMA4A/SNCA/ROBO2/LGI2/NPTX1/RAB29/PCDHB10/LRFN1/PCDHB14/RAB17/LRRTM2/NRXN2/SNAP25/NTRK2/THBS2/DNER/MDGA1/AMIGO1/GAP43/GABRG1/GPM6A/GABRA6/GABRA4/GABRB3/ADD2/LRFN4/EPHB1/PCDHB2/SDK1/ARHGEF9/CLSTN3/SLIT1/LRRC4B/PTK2B/ABI3/LRFN3/ARMCX5-GPRASP2/EFNB3/DVL1/ADGRF1/CLSTN1/PLXNB2/NLGN2/DBNL* | 82 |
| BP | GO:0099003 | vesicle-mediated transport in synapse | 8.754734137 | 5.29E-15 | 3.23E-12 | 2.86E-12 | *RAB27B/SNCB/SLC17A8/PACSIN1/DOC2B/UNC13C/P2RX7/SLC17A7/DNM3/RIMS1/SV2C/SLC17A6/TH/PPFIA2/GRIP2/CALY/HPCA/DNAJC6/SYNJ2/SNAP91/SYN1/SYT5/SH3GL3/SH3GL2/SNPH/SNCA/PRKN/CPLX1/ERC2/STX1B/UNC13A/SYT7/SYP/SYT4/PRKAR1B/RAB3A/CADPS/GRIK5/STX1A/NAPB/OPHN1/PRKCG/STXBP1/NSG1/DOC2A/SNAP25/PPFIA3/TBC1D24/RIMS4/CDK5R1/PPP3CC/SYT1/AP3B2/SNAP23/BTBD9/BRSK1/SV2A/CSPG5/CPLX2/VAMP2/RIMS3/ATP6V1A/CYFIP1/SLC4A8/DNAJC5/BIN1/ATP6V0A1/DGKQ/PLD2/PPP3CB/PIP5K1C/SYT11/FBXL20/AP2A1/GIT1/DVL1/SCRN1/VPS18/CLSTN1/ATP6V1B2/AP3D1/GAK/NLGN2/VAC14/AP3M2* | 85 |
| BP | GO:0035249 | synaptic transmission, glutamatergic | 8.657946237 | 6.30E-14 | 2.74E-11 | 2.43E-11 | *PLPPR4/GRIN3A/GRM1/GRIN1/GRM7/CACNG5/SLC17A8/GRM5/TNR/UNC13C/GRM4/CACNG2/SLC17A7/GRID2/GRIK1/SLC17A6/GRIA2/GRIK3/GRIN2A/GRID1/MEF2C/GRIK2/FRRS1L/ROR2/GRM8/NRXN1/RELN/GRIN2D/GRIA3/HTR2A/DGKI/IQSEC2/GRIN2C/CNR1/PRKN/GRIK4/UNC13A/GRIK5/NAPB/OPHN1/CLN3/STXBP1/UCN/SLC1A4/SYT1/MAPK8IP2/SERPINE2/CLSTN3/PTK2B/DGKZ/EXT1/CACNG7/NLGN2* | 53 |
| CC | GO:0008328 | ionotropic glutamate receptor complex | 8.440075822 | 2.99E-12 | 9.94E-11 | 8.20E-11 | *GRIN3A/GRIN1/CACNG5/CACNG2/GRID2/GRIK1/GRIA2/GRIK3/GRIN2A/GRIK2/SHISA8/VWC2L/VWC2/SHISA7/GRIN2D/GRIA3/GRIN2C/GRIK4/GRIK5/OLFM2/ABHD6/DLG4/PTK2B/PORCN/CNIH2/CPT1C/CACNG7* | 27 |
| CC | GO:0098982 | GABA-ergic synapse | 8.094229049 | 3.94E-12 | 1.25E-10 | 1.03E-10 | *SLC6A17/GABRB1/GABRA2/GABRG3/KCNC2/CNTN5/ATP2B3/KCND2/SLITRK3/GABRG2/ATP2B2/LRFN5/GABRA5/NPY5R/IQSEC3/GAD1/GABRA1/SLC6A1/GRID1/GUCY1A1/LRRTM1/SLITRK1/LHFPL4/PTPRO/CDH13/GLRB/CNR1/ERC2/LRRTM2/MDGA1/ABHD6/GAP43/BAIAP3/GABRA4/SV2A/LRFN4/CSPG5/ARHGEF9/CLSTN3/NLGN2* | 40 |
| BP | GO:0006836 | neurotransmitter transport | 8.029522625 | 5.71E-13 | 2.05E-10 | 1.82E-10 | *SLC6A17/SLC17A8/DOC2B/UNC13C/GRM4/P2RX7/SLC18A3/SLC17A7/RIMS1/SV2C/GPR158/SLC17A6/TH/PPFIA2/SLC6A1/MEF2C/SLC1A1/GABRQ/NRXN1/SYN1/SYT5/TACR2/SYNGR3/SNPH/SNCA/SLC1A2/PRKN/CPLX1/SLC6A15/CAMK2A/ERC2/STX1B/LIN7A/UNC13A/SYT7/SYP/LIN7B/SYT4/NRXN2/RAB3A/CADPS/SLC6A7/GRIK5/STX1A/NAPB/PRKCG/STXBP1/DOC2A/SNAP25/PPFIA3/RIMS4/SYT1/BAIAP3/SNAP23/BRSK1/SV2A/SNCAIP/ASIC1/CSPG5/CPLX2/VAMP2/RIMS3/SLC4A8/DNAJC5/SLC29A1/KCNMB4/PIP5K1C/SYT11/ITGB1/SLC18B1/SLC29A2/FBXL20/SLC29A4/GIT1/DVL1/VPS18/PPT1* | 77 |
| BP | GO:0007269 | neurotransmitter secretion | 8.088711368 | 9.47E-13 | 3.04E-10 | 2.69E-10 | *DOC2B/UNC13C/GRM4/P2RX7/SLC18A3/RIMS1/SV2C/GPR158/PPFIA2/MEF2C/NRXN1/SYN1/SYT5/TACR2/SNPH/SNCA/PRKN/CPLX1/CAMK2A/ERC2/STX1B/LIN7A/UNC13A/SYT7/SYP/LIN7B/SYT4/NRXN2/RAB3A/CADPS/GRIK5/STX1A/NAPB/PRKCG/STXBP1/DOC2A/SNAP25/PPFIA3/RIMS4/SYT1/BAIAP3/SNAP23/BRSK1/SV2A/SNCAIP/ASIC1/CSPG5/CPLX2/VAMP2/RIMS3/SLC4A8/DNAJC5/KCNMB4/PIP5K1C/SYT11/FBXL20/GIT1/DVL1/VPS18/PPT1* | 60 |
| BP | GO:0050803 | regulation of synapse structure or activity | 7.798141986 | 1.49E-12 | 4.53E-10 | 4.02E-10 | *SLITRK6/ICAM5/ZNF804A/SLC17A8/SLITRK3/CBLN1/LINGO2/IL10RA/CAMK2B/LRFN5/SLC18A3/SLC17A7/DNM3/NEGR1/GRID2/GPR158/SLC17A6/LRFN2/PPFIA2/NGEF/CBLN2/BDNF/GRID1/MEF2C/ROR2/CLSTN2/LRRTM3/LRRTM1/NRXN1/RELN/SLITRK1/LHFPL4/NTNG2/SRCIN1/PTPRO/NEURL1/ADGRB3/C1QL1/SHANK2/APOE/SEMA4A/EFNA1/SNCA/FRMPD4/ROBO2/ARHGAP44/LRFN1/RAB17/LRRTM2/ZDHHC15/PRNP/CLN3/SNAP25/NTRK2/LZTS3/CDK5R1/PAK3/THBS2/MDGA1/AMIGO1/SYBU/GPM6A/KIF1A/LRFN4/EPHB1/ADGRL1/CYFIP1/CTNNA2/CNTNAP1/CLSTN3/SLIT1/LRRC4B/PTK2B/ABI3/LRFN3/DAB2IP/ITGB1/ARMCX5-GPRASP2/EFNB3/ABHD17A/DVL1/CLSTN1/PPT1/ABL1/TUBA1A/CDC42/NLGN2* | 87 |
| CC | GO:0098878 | neurotransmitter receptor complex | 7.638506941 | 1.48E-10 | 4.13E-09 | 3.40E-09 | *GRIN3A/GRIN1/CACNG5/CACNG2/GRID2/GRIK1/GRIA2/GRIK3/GRIN2A/GRIK2/SHISA8/VWC2L/VWC2/SHISA7/GRIN2D/GRIA3/GRIN2C/GRIK4/GRIK5/OLFM2/ABHD6/DLG4/PTK2B/PORCN/CNIH2/CPT1C/CACNG7* | 27 |
| MF | GO:0030594 | neurotransmitter receptor activity | 7.742632341 | 1.27E-11 | 5.01E-09 | 4.28E-09 | *CHRNA6/GABRB1/GABRA2/GRIN3A/GRM1/GRIN1/CHRM2/GLRA3/CHRNB3/GABRG3/GRM5/GABRG2/GABRA3/CHRM4/GLRA2/GABRA5/CHRM3/GRID2/GPR158/GRIK1/GRIA2/GRIK3/GABRA1/GRIN2A/GRID1/GRIK2/GABRQ/GRIN2D/GRIA3/HTR2A/CHRM5/GRIN2C/GLRB/HTR1E/GRIK4/CHRNA4/CHRNA7/GRIK5/ANXA9/GABRG1/GABRA6/GABRA4/GABRB3/HTR5A/PTK2B/P2RY11* | 46 |
| BP | GO:0007215 | glutamate receptor signaling pathway | 7.858656053 | 4.16E-11 | 1.15E-08 | 1.02E-08 | *GRIN3A/GRM1/GRIN1/GRM7/GRM5/GRM4/SSTR1/GRID2/GRIK1/GRIA2/GRIK3/GRIN2A/GRID1/GRIK2/SLC1A1/FRRS1L/GRM8/GRIN2D/GRIA3/GRIN2C/KCNB1/CPEB4/GRIK4/DAGLA/GRIK5/PRNP/CLN3/CDK5R1/PTK2B* | 29 |
| BP | GO:0050807 | regulation of synapse organization | 7.199144216 | 4.92E-11 | 1.30E-08 | 1.16E-08 | *SLITRK6/ICAM5/ZNF804A/SLITRK3/CBLN1/LINGO2/IL10RA/CAMK2B/LRFN5/SLC18A3/DNM3/NEGR1/GRID2/GPR158/LRFN2/PPFIA2/NGEF/CBLN2/BDNF/GRID1/MEF2C/ROR2/CLSTN2/LRRTM3/LRRTM1/NRXN1/RELN/SLITRK1/LHFPL4/NTNG2/SRCIN1/PTPRO/NEURL1/ADGRB3/C1QL1/SHANK2/APOE/SEMA4A/EFNA1/SNCA/FRMPD4/ROBO2/ARHGAP44/LRFN1/RAB17/LRRTM2/ZDHHC15/PRNP/CLN3/SNAP25/NTRK2/LZTS3/CDK5R1/PAK3/THBS2/MDGA1/AMIGO1/GPM6A/KIF1A/LRFN4/EPHB1/ADGRL1/CYFIP1/CTNNA2/CNTNAP1/CLSTN3/SLIT1/LRRC4B/PTK2B/ABI3/LRFN3/DAB2IP/ITGB1/ARMCX5-GPRASP2/EFNB3/ABHD17A/DVL1/CLSTN1/ABL1/TUBA1A/CDC42/NLGN2* | 82 |
| CC | GO:0048786 | presynaptic active zone | 7.076604123 | 8.15E-10 | 1.82E-08 | 1.50E-08 | *GABRB1/GRM7/APBA1/UNC13C/ATP2B2/ADCY8/SLC17A7/RIMS1/PPFIA4/GAD1/PPFIA2/NTNG1/GRIN2D/NTNG2/SYN1/SHANK2/ARHGAP44/ERC2/UNC13A/SYP/RAB3A/STX1A/STXBP1/SNAP25/PPFIA3/RIMS4/GUCY1B1/ERC1/GPM6A/BRSK1/SV2A/RIMS3/CNTNAP1/ATP2B4/LRFN3/SYT11* | 36 |
| CC | GO:0060076 | excitatory synapse | 7.146710561 | 8.33E-10 | 1.82E-08 | 1.50E-08 | *KCNJ3/GRIN1/ATP2B3/SLC17A8/PLXNA4/CBLN1/UNC13C/ATP2B2/CALB2/ADCY8/SLC17A7/KCNJ9/GRID2/SLC17A6/GRIA2/LRRTM1/GRIA3/SHANK1/ADGRB3/C1QL1/P2RX6/SYP/LRRTM2/STXBP1/SYT1/GPM6A/CYFIP1/DLG4/SYT11/ITGB1/SORT1/NLGN2* | 32 |
| MF | GO:0008066 | glutamate receptor activity | 7.779806783 | 1.52E-10 | 2.25E-08 | 1.92E-08 | *GRIN3A/GRM1/GRIN1/GRM7/GRM5/GRM4/GRID2/GRIK1/GRIA2/GRIK3/GRIN2A/GRID1/GRIK2/GRM8/GRIN2D/GRIA3/GRIN2C/GRIK4/GRIK5/PTK2B* | 20 |
| BP | GO:0006813 | potassium ion transport | 6.590436374 | 1.32E-09 | 2.60E-07 | 2.30E-07 | *KCNJ3/KCNK9/KCNC2/KCNA4/KCND2/GCK/SLC24A2/GAL/NALCN/KCNH3/KCNS2/KCNQ3/SLC17A7/CACNA1D/KCNJ9/SLC17A6/SLC24A3/RGS7/KCNB2/SLC12A5/KCNK3/KCNIP4/KCNS3/LRRC26/SLC9A9/KCNA2/FXYD2/KCNH5/HCN2/ABCC9/HTR2A/KCNG2/SLC9A7/KCNIP1/KCNB1/KCNJ11/RNF207/KCNK7/KCNN3/GJA5/DPP10/KCNG1/PRNP/KCNV1/AKAP6/SNAP25/ANK2/KCNIP2/KCNH2/LRRC55/ATP1A3/NOS1AP/AMIGO1/SLC9A6/ATP1B2/KCNAB2/SLC9A2/SLC12A6/KCNE1/TMEM175/ATP1B1/VAMP2/NEDD4L/NSF/TMEM38A/BIN1/KCNK15/PTK2B/KCNMB4/KCNQ2/ITGB1/ABCB8/KCNJ1/SLC9A1/SLC9A4/FHL1/TMCO3/ATP1B3* | 78 |
| BP | GO:0043269 | regulation of monoatomic ion transport | 5.860628269 | 2.42E-08 | 3.08E-06 | 2.73E-06 | *KCNJ3/GRIN1/KCNC2/GCK/GRM5/RYR2/PCSK9/GAL/NTSR1/CAMK2B/KCNS2/P2RX7/CACNA1D/KCNJ9/SCN2B/CNTN1/ACE/RGS7/STOM/SPTBN4/LYN/FGF11/FGF14/MAOB/P2RX5/HECW1/CACNA1C/GRIN2A/KCNIP4/HPCA/LRRC26/ABCB1/RGS9/BEST1/CACNB2/PTPN3/FXYD2/NKAIN2/TESC/NKAIN3/TF/RELN/GRIN2D/CTSS/PLA2G1B/HTR2A/SCN3B/SLC8A1/TACR2/KCNIP1/GRIN2C/TRPC6/JPH3/CNR1/MYLK/TCAF2/CAPN3/SNCA/GPR35/CAMK2A/MCHR1/KCNJ11/RNF207/GPD1L/UBASH3B/IL16/DPP10/KCNG1/PRNP/FXYD7/AKAP6/JPH4/SNTA1/PXK/ANK2/PRKCE/UCN/WNK2/KCNIP2/CACNB1/PPP3CC/KCNH2/LRRC55/PDE4D/NOS1AP/AMIGO1/PIRT/ATP1B2/KCNAB2/GNB5/KCNE1/ATP1B1/MLLT6/ORAI1/VAMP2/NEDD4L/SERPINE2/TMEM38A/BIN1/STIM1/GSTO1/PTK2B/FKBP1B/CXCR4/CBARP/ATP2B4/PPP3CB/ITGB1/KCNJ1/PSEN2/SARAF/RAB11B/FXYD6/CAMK2G/SLC9A1/PRKACA/PLN/PPP3R2/PML/ABL1/CALM1/FHL1/CALM3/ATP1B3/YWHAQ* | 125 |
| BP | GO:0098815 | modulation of excitatory postsynaptic potential | 6.466321553 | 3.18E-08 | 3.73E-06 | 3.31E-06 | *GRIN1/CBLN1/RIMS1/GRIN2A/GRIK2/CELF4/SLC8A3/NRXN1/RELN/GRIN2D/SHANK1/SLC8A2/GRIN2C/TMEM108/CHRNA7/STX1B/PRKAR1B/STX1A/TBC1D24/PRKCZ/DLG4/PTK2B/TMEM25/DVL1/NLGN2* | 25 |
| BP | GO:0035418 | protein localization to synapse | 6.0949567 | 7.65E-08 | 7.52E-06 | 6.67E-06 | *RAB27B/SLITRK3/LRRC7/CACNG2/MAPT/GRIP2/GRIN2A/NPHS1/LRRTM1/NRXN1/RELN/LHFPL4/SHANK1/IQSEC2/GRIN2C/ARHGAP44/CPLX1/NPTX1/STX1B/NRXN2/ZDHHC15/NSG1/SNAP25/KIF5A/PRKCZ/CLSTN3/DLG4/ERBB2/DVL1/MAPK8IP3/CLSTN1/CACNG7/NLGN2* | 33 |
| BP | GO:0051588 | regulation of neurotransmitter transport | 5.549205584 | 5.69E-07 | 4.23E-05 | 3.75E-05 | *SLC17A8/SLC18A3/RIMS1/SV2C/GPR158/PPFIA2/MEF2C/SYN1/TACR2/SYNGR3/SNCA/PRKN/CPLX1/CAMK2A/STX1B/SYP/SYT4/RAB3A/STX1A/PRKCG/STXBP1/RIMS4/SYT1/BAIAP3/SNCAIP/ASIC1/CSPG5/CPLX2/RIMS3/SLC4A8/KCNMB4/SYT11/ITGB1/FBXL20/GIT1/DVL1/VPS18* | 37 |
| BP | GO:0014059 | regulation of dopamine secretion | 5.671313277 | 1.26E-06 | 8.38E-05 | 7.43E-05 | *CHRNA6/KCNA2/SYT13/SYT3/HTR2A/SYT5/CNR1/SNCA/PRKN/CHRNA4/SYT7/SYT15/SYT4/SYT1/PINK1/SYT17/FGF20/SYT11/ABAT* | 19 |
| BP | GO:0048499 | synaptic vesicle membrane organization | 5.755612941 | 1.35E-06 | 8.87E-05 | 7.87E-05 | *DOC2B/CPLX1/ERC2/STX1B/SYP/RAB3A/GRIK5/STX1A/STXBP1/DOC2A/SNAP25/SYT1/SNAP23/SYNGR1/CPLX2/AP3D1* | 16 |
| BP | GO:0099601 | regulation of neurotransmitter receptor activity | 5.286249128 | 4.07E-06 | 0.000221369 | 0.000196388 | *CACNG5/OPRM1/CACNG2/PSCA/MEF2C/SHISA8/RELN/SHISA7/LYNX1/SHANK1/DLGAP3/BEGAIN/PRRT1/MAPK8IP2/DLG4/PTK2B/CNIH2/CAPN1/MINK1/CACNG7/NLGN2* | 21 |
| BP | GO:0006820 | monoatomic anion transport | 4.645191919 | 1.25E-05 | 0.000552679 | 0.000490312 | *SLC4A10/GABRB1/GABRA2/GLRA3/GABRG3/SLC17A8/GRM5/GABRG2/GABRA3/GLRA2/SLC17A7/GABRA5/SLC17A6/SLC26A1/SLC12A5/GABRA1/SLC6A1/OCA2/ABCB1/SLC1A1/GABRQ/BEST1/TG/ABCC9/GLRB/TCAF2/SLC1A2/CLCN4/SLC4A3/CLIC5/PRNP/SLC1A4/GABRG1/GABRA6/GABRA4/GABRB3/SLC12A6/UCP2/SLC4A8/SLC17A5/SLC26A8/SLC26A11/SLC39A14/SLC26A6/ABCC10/SLC26A9/RAB11B/CLCN7/SLC19A1/TTYH3/CLNS1A* | 51 |
| BP | GO:0006816 | calcium ion transport | 4.42743119 | 1.63E-05 | 0.00064779 | 0.000574689 | *GRIN3A/ADRA1A/GRIN1/CACNG5/ATP2B3/GCK/SLC24A2/RYR2/CACNA1I/NALCN/OPRM1/NTSR1/ATP2B2/CAMK2B/P2RX7/CACNG2/CACNA1E/CACNA2D3/CACNA1D/ACE/SLC24A3/LYN/TMEM37/P2RX5/CACNA1C/GRIN2A/HPCA/RGS9/SLC8A3/PLCB2/PLCH2/BEST1/CACNB2/CACNA1A/GRIN2D/PLA2G1B/HTR2A/SLC8A2/SLC8A1/CACNA1B/GRIN2C/TRPC6/JPH3/MYLK/CAPN3/SNCA/GPR35/CHRNA4/TRPA1/CAMK2A/CHRNA7/MCHR1/P2RX6/UBASH3B/NOL3/IL16/PKD1L1/CACNA2D2/PRNP/AKAP6/JPH4/ANK2/PRKCE/UCN/CACNB1/PPP3CC/TRPC5/PDE4D/NOS1AP/TRPM4/ANXA9/NCS1/GPM6A/GNB5/MCOLN1/ATP1B1/ASIC1/ORAI1/TMEM38A/BIN1/STIM1/GSTO1/PTK2B/FKBP1B/CXCR4/GAS6/CBARP/ATP2B4/ORAI2/PPP3CB/MCOLN3/SLC25A25/PSEN2/SARAF/CAMK2G/SLC9A1/PRKACA/PLN/SLC25A23/PPP3R2/CACNG7/PML/ABL1/CALM1/CALM3* | 105 |
| CC | GO:0098685 | Schaffer collateral - CA1 synapse | 4.303949349 | 6.79E-05 | 0.000816975 | 0.000673934 | *GABRB1/GRM1/APBA1/GRM5/TNR/CACNG2/ADCY8/LRFN2/NTNG1/SLC1A1/NTNG2/SYN1/ADCY1/SHANK1/IQSEC2/LRRC4C/CPLX1/SYP/LRRTM2/PRKAR1B/INA/DCC/PRKCZ/PTK2B/PLG/ITGB1/FBXL20/TUBB2B/DVL1/CDC42/PTPRA* | 31 |
| CC | GO:0098831 | presynaptic active zone cytoplasmic component | 4.417666528 | 0.000199635 | 0.002177274 | 0.001796062 | *UNC13C/RIMS1/ERC2/UNC13A/STXBP1/PPFIA3/RIMS4/GUCY1B1/ERC1/RIMS3* | 10 |
| MF | GO:0005313 | L-glutamate transmembrane transporter activity | 4.633269082 | 0.00012326 | 0.002396506 | 0.002046187 | *SLC17A8/SLC17A7/SLC17A6/SLC1A1/SLC1A2/SLC25A18/SLC25A13/SLC25A12/SLC25A22* | 9 |
| BP | GO:0099637 | neurotransmitter receptor transport | 4.567639601 | 8.87E-05 | 0.002549692 | 0.002261969 | *CACNG5/LRRC7/CACNG2/GRIP2/SLC1A1/ARHGAP44/CPLX1/STX1B/NSG1/SNAP25/CLSTN1/CACNG7/AP3D1* | 13 |
| BP | GO:0006814 | sodium ion transport | 4.042466653 | 9.28E-05 | 0.002629709 | 0.002332957 | *SLC6A17/SLC4A10/SCN2A/SLC17A8/SLC24A2/PCSK9/NALCN/ASIC4/P2RX7/SLC17A7/SCN9A/SCN2B/CNTN1/SLC17A6/SLC24A3/SPTBN4/FGF11/FGF14/HECW1/SLC6A1/SLC13A5/SLC9A9/SLC8A3/PTPN3/FXYD2/NKAIN2/TESC/NKAIN3/HCN2/SCN3B/SLC8A2/SLC8A1/SLC9A7/SCN3A/SLC9B1/SLC6A15/SGK1/GPD1L/SLC6A7/FXYD7/SNTA1/PRKCE/WNK2/ATP1A3/TRPM4/SLC9A6/ATP1B2/SLC9A2/SLC38A3/SLC9B2/ATP1B1/ASIC1/MLLT6/NEDD4L/SLC4A8/SERPINE2/ATP2B4/SLC20A1/FXYD6/SLC9A1/NDUFA9/SLC41A3/SLC9A4/ATP1B3* | 64 |
| BP | GO:1903539 | protein localization to postsynaptic membrane | 4.324278697 | 0.000116633 | 0.003117376 | 0.002765592 | *LRRC7/CACNG2/GRIP2/GRIN2A/LHFPL4/IQSEC2/GRIN2C/ARHGAP44/CPLX1/NPTX1/STX1B/NSG1/SNAP25/PRKCZ/DLG4/ERBB2/CLSTN1/CACNG7* | 18 |
| CC | GO:0043083 | synaptic cleft | 4.185700163 | 0.000324318 | 0.003280784 | 0.002706362 | *GRIN1/CBLN1/DNM3/PRSS12/LAMA2/SLC1A1/ACHE/ADGRB3/C1QL1/APOE/NPTX1* | 11 |
| BP | GO:0010821 | regulation of mitochondrion organization | 3.574397267 | 0.000560151 | 0.011155421 | 0.009896576 | *KDR/MAPT/HRK/PPARGC1A/MGARP/MOAP1/PID1/TMEM102/FZD9/PRKN/PDE2A/NOL3/PMAIP1/PLAUR/OMA1/PINK1/INF2/IRGM/CLU/ATP13A2/MIEF2/SLC35F6/FXN/TMEM14A/SLC25A4/MLLT11/HIP1R/TIGAR/CERS1/BCL2L11/BCL2L1/ACAA2/ZNF205/AMBRA1/VAT1/RAP1GDS1/USP30/TSC2/STAT2/PSMD10/MFF* | 41 |
| BP | GO:0072578 | neurotransmitter-gated ion channel clustering | 4.018204773 | 0.000654394 | 0.012540493 | 0.011125347 | *SLITRK3/FRRS1L/RELN/SHISA7/LHFPL4/APOE/GLRB/DLG4/NLGN2* | 9 |
| BP | GO:0098877 | neurotransmitter receptor transport to plasma membrane | 4.018204773 | 0.000654394 | 0.012540493 | 0.011125347 | *LRRC7/GRIP2/SLC1A1/ARHGAP44/CPLX1/STX1B/NSG1/SNAP25/CLSTN1* | 9 |
| BP | GO:0099072 | regulation of postsynaptic membrane neurotransmitter receptor levels | 3.541322666 | 0.000801948 | 0.014720093 | 0.013058988 | *CACNG5/LRRC7/CACNG2/DNM3/TFR2/GRIP2/CALY/HPCA/FRRS1L/LHFPL4/IQSEC2/ARHGAP44/CPLX1/NPTX1/STX1B/OPHN1/NSG1/SNAP25/PRKCZ/DLG4/PORCN/ERBB2/AP2A1/CPT1C/CLSTN1/CACNG7/VAC14* | 27 |
| MF | GO:0005326 | neurotransmitter transmembrane transporter activity | 3.658988604 | 0.001351486 | 0.018423707 | 0.015730546 | *SLC17A8/SLC18A3/SLC17A7/SLC17A6/GABRQ/CPLX1/SLC6A15/SLC29A1/SLC29A2/SLC29A4* | 10 |
| MF | GO:0099528 | G protein-coupled neurotransmitter receptor activity | 3.745027963 | 0.001664765 | 0.021696827 | 0.018525204 | *GABRB1/GRM1/CHRM2/CHRM4/CHRM3/GPR158/CHRM5* | 7 |
| CC | GO:0098845 | postsynaptic endosome | 3.338361719 | 0.004169892 | 0.030522158 | 0.025178134 | *SH3GL3/NSG1/PICK1/STX12/ABHD17A/CLSTN1/RAB4A* | 7 |
| BP | GO:0099560 | synaptic membrane adhesion | 3.362396648 | 0.002571092 | 0.037937618 | 0.033656505 | *CDH9/SLITRK3/LRFN5/NTNG1/SLITRK1/NTNG2/LRRC4C/MDGA1/LRFN4/LRRC4B/LRFN3* | 11 |
| BP | GO:0050769 | positive regulation of neurogenesis | 2.935252963 | 0.003091845 | 0.042822049 | 0.037989747 | *DSCAM/TNFRSF1B/DCT/GRM5/SHOX2/PLXNA4/OPRM1/PLXNB3/HAPLN2/CAMK2B/ACE/MAPT/CHODL/LYN/PRKCH/ISLR2/ZNF365/BDNF/HAPLN3/RELN/RNF112/SLITRK1/NEURL1/ZNF488/IL34/TTBK1/ROBO2/CDH4/ASCL1/NTRK2/TBC1D24/TRPC5/AMIGO1/PLXNA2/L1CAM/TENM4/CYFIP1/MAP3K13/SERPINE2/MEGF8/BIN1/ROBO1/CXCR4/NCAN/SMO/PLXNA3/ITGB1/ASPA/ZFYVE27/SRF/NUMBL/ARMCX5-GPRASP2/OBSL1/LIMK1/TRAK1/CUL7/GDI1/PLXNB2* | 58 |
| BP | GO:0007212 | G protein-coupled dopamine receptor signaling pathway | 3.118374526 | 0.003797803 | 0.049452589 | 0.043872056 | *CALY/SLC1A1/RGS9/RGS8/ADCY5/GNAL/NSG2/NSG1/GNB5/GNAO1/GNG2/PALM/GNA11/ADCY6* | 14 |

**Table S11 KEGG enrichment results in astrocyte dataset**

| category | subcategory | ID | Description | zScore | pvalue | p.adjust | qvalue | geneID | Count |
| --- | --- | --- | --- | --- | --- | --- | --- | --- | --- |
| Human Diseases | Neurodegenerative disease | hsa05014 | Amyotrophic lateral sclerosis | 8.177111931 | 2.12E-11 | 5.99E-09 | 5.72E-09 | *MAPK12/CASP9/NDUFA12/NDUFA4/COX7A2/ACTB/ATF6/NDUFB1/ACTG1/PSMB7/PSMB4/PSMA2/CCS/ATP5F1C/ACTR1B/NDUFAB1/ANG/NDUFS7/PSMD13/NDUFS6/PSMD2/ANXA7/PSMD8/NDUFA13/KLC1/PFN1/DCTN2/KLC2/ATP5F1E/PSMC3/PSMA7/NCBP1/NDUFB7/PSMB2/PSMB1/BAD/NDUFS3/PSMC2/NDUFC2-KCTD14/PSMD9/SEC13/PSMC5* | 42 |
| Human Diseases | Neurodegenerative disease | hsa05012 | Parkinson disease | 7.716108021 | 5.43E-10 | 7.69E-08 | 7.35E-08 | *CASP9/NDUFA12/NDUFA4/CALML4/COX7A2/ATF6/NDUFB1/PSMB7/PSMB4/PSMA2/ATP5F1C/NDUFAB1/NDUFS7/PSMD13/NDUFS6/PSMD2/PSMD8/NDUFA13/KLC1/KLC2/ATP5F1E/PSMC3/VDAC3/PSMA7/NDUFB7/PSMB2/PSMB1/PARK7/NDUFS3/PSMC2/NDUFC2-KCTD14/PSMD9/PSMC5* | 33 |
| Human Diseases | Neurodegenerative disease | hsa05020 | Prion disease | 7.539175072 | 1.06E-09 | 9.65E-08 | 9.23E-08 | *MAPK12/CASP9/NDUFA12/NDUFA4/CREB5/COX7A2/NDUFB1/PSMB7/PSMB4/PSMA2/ATP5F1C/NDUFAB1/NDUFS7/PSMD13/NDUFS6/PSMD2/PSMD8/NDUFA13/KLC1/KLC2/ATP5F1E/PSMC3/VDAC3/PSMA7/NDUFB7/PSMB2/PSMB1/BAD/NDUFS3/PSMC2/NDUFC2-KCTD14/PSMD9/PSMC5* | 33 |
| Human Diseases | Neurodegenerative disease | hsa05016 | Huntington disease | 7.395004114 | 1.36E-09 | 9.65E-08 | 9.23E-08 | *CASP9/NDUFA12/NDUFA4/CREB5/COX7A2/NDUFB1/POLR2I/PSMB7/PSMB4/PSMA2/ATP5F1C/ACTR1B/NDUFAB1/NDUFS7/PSMD13/NDUFS6/PSMD2/PSMD8/NDUFA13/KLC1/DCTN2/POLR2J/KLC2/ATP5F1E/PSMC3/VDAC3/PSMA7/NDUFB7/PSMB2/PSMB1/NDUFS3/PSMC2/NDUFC2-KCTD14/PSMD9/PSMC5* | 35 |
| Genetic Information Processing | Folding, sorting and degradation | hsa03050 | Proteasome | 9.013505992 | 4.52E-09 | 2.56E-07 | 2.44E-07 | *PSMB7/PSMB4/PSMA2/PSMD13/PSMD2/PSMD8/PSMC3/PSMA7/PSMB2/PSMB1/PSMC2/PSMD9/PSMC5* | 13 |
| Human Diseases | Neurodegenerative disease | hsa05010 | Alzheimer disease | 6.106189572 | 1.58E-07 | 7.43E-06 | 7.10E-06 | *CASP9/NDUFA12/ATP2A3/NDUFA4/GAPDH/CALML4/COX7A2/ATF6/NDUFB1/PSMB7/PSMB4/PSMA2/ATP5F1C/NDUFAB1/NDUFS7/PSMD13/NDUFS6/PSMD2/PSMD8/NDUFA13/KLC1/KLC2/ATP5F1E/PSMC3/VDAC3/PSMA7/NDUFB7/PSMB2/PSMB1/CAPN1/BAD/NDUFS3/PSMC2/NDUFC2-KCTD14/PSMD9/PSMC5* | 36 |
| Human Diseases | Neurodegenerative disease | hsa05022 | Pathways of neurodegeneration - multiple diseases | 5.70005685 | 5.24E-07 | 2.12E-05 | 2.03E-05 | *MAPK12/CASP9/NDUFA12/ATP2A3/NDUFA4/CALML4/COX7A2/ATF6/NDUFB1/PSMB7/PSMB4/PSMA2/CCS/ATP5F1C/ACTR1B/NDUFAB1/NDUFS7/PSMD13/NDUFS6/PSMD2/PSMD8/NDUFA13/KLC1/DCTN2/KLC2/ATP5F1E/PSMC3/VDAC3/PSMA7/NDUFB7/PSMB2/PSMB1/PARK7/CAPN1/BAD/NDUFS3/PSMC2/NDUFC2-KCTD14/PSMD9/PSMC5* | 40 |
| Organismal Systems | Environmental adaptation | hsa04714 | Thermogenesis | 5.173703822 | 1.14E-05 | 0.000404085 | 0.000386274 | *MGLL/MAPK12/PRKAA2/NDUFA12/COX17/NDUFA4/CREB5/COX7A2/ACTB/NDUFB1/ACTG1/ATP5F1C/NDUFAB1/NDUFS7/NDUFS6/NDUFA13/NDUFAF3/ATP5F1E/NDUFB7/SMARCC1/NDUFS3/NDUFAF2/NDUFC2-KCTD14* | 23 |
| Metabolism | Glycan biosynthesis and metabolism | hsa00510 | N-Glycan biosynthesis | 5.832004209 | 2.15E-05 | 0.000676485 | 0.000646668 | *ALG10B/DHRSX/ALG11/KRTCAP2/ALG9/ALG14/TUSC3/GANAB/STT3A/DDOST* | 10 |
| Human Diseases | Cardiovascular disease | hsa05415 | Diabetic cardiomyopathy | 4.799252188 | 4.50E-05 | 0.00127481 | 0.001218621 | *AGTR1/MAPK12/NDUFA12/ATP2A3/NDUFA4/GAPDH/COX7A2/REN/NDUFB1/ATP5F1C/NDUFAB1/NDUFS7/NDUFS6/NDUFA13/ATP5F1E/VDAC3/NDUFB7/NDUFS3/PPP1CA/NDUFC2-KCTD14* | 20 |
| Human Diseases | Neurodegenerative disease | hsa05017 | Spinocerebellar ataxia | 4.888795488 | 5.58E-05 | 0.001434834 | 0.001371591 | *ATP2A3/TWNK/PSMB7/PSMB4/PSMA2/PSMD13/PSMD2/PSMD8/PSMC3/VDAC3/PSMA7/PSMB2/PSMB1/PSMC2/PSMD9/PSMC5* | 16 |
| Metabolism | Glycan biosynthesis and metabolism | hsa00513 | Various types of N-glycan biosynthesis | 5.302395819 | 0.000122525 | 0.002889547 | 0.002762186 | *ALG11/ST3GAL3/KRTCAP2/ALG9/ALG14/TUSC3/STT3A/DDOST* | 8 |
| Organismal Systems | Nervous system | hsa04723 | Retrograde endocannabinoid signaling | 4.283680054 | 0.000287274 | 0.006253742 | 0.005978098 | *GABRE/MGLL/MAPK12/NDUFA12/NDUFA4/NAPEPLD/NDUFB1/NDUFAB1/NDUFS7/NDUFS6/NDUFA13/NDUFB7/GNB2/NDUFS3/NDUFC2-KCTD14* | 15 |
| Metabolism | Energy metabolism | hsa00190 | Oxidative phosphorylation | 4.169343333 | 0.000421158 | 0.008513415 | 0.008138173 | *NDUFA12/COX17/NDUFA4/COX7A2/NDUFB1/ATP5F1C/NDUFAB1/NDUFS7/NDUFS6/NDUFA13/ATP5F1E/NDUFB7/NDUFS3/NDUFC2-KCTD14* | 14 |
| Human Diseases | Endocrine and metabolic disease | hsa04932 | Non-alcoholic fatty liver disease | 3.617913934 | 0.001513805 | 0.02856046 | 0.027301611 | *MAPK12/PRKAA2/NDUFA12/NDUFA4/COX7A2/NDUFB1/NDUFAB1/NDUFS7/NDUFS6/NDUFA13/NDUFB7/MLX/NDUFS3/NDUFC2-KCTD14* | 14 |
| Human Diseases | Infectious disease: bacterial | hsa05132 | Salmonella infection | 3.436137311 | 0.001711793 | 0.030277342 | 0.028942819 | *PAK3/MAPK12/GAPDH/DYNLT1/ACTB/RAB9B/ACTG1/RRAS/ACTR1B/CD14/RHOA/KLC1/PFN1/DCTN2/KLC2/ARPC2/ARL8B/CYFIP1/ARPC4* | 19 |
| Cellular Processes | Transport and catabolism | hsa04142 | Lysosome | 3.390967243 | 0.002930381 | 0.048782217 | 0.046632062 | *MANBA/IDUA/CTSA/ARSA/GALNS/SCARB2/MEAK7/ABCA2/AP3B2/DNASE2/FUCA2/AP4M1* | 12 |

**Table S12 GO enrichment results in astrocyte dataset**

| ONTOLOGY | ID | Description | zScore | pvalue | p.adjust | qvalue | geneID | Count |
| --- | --- | --- | --- | --- | --- | --- | --- | --- |
| BP | GO:1901748 | leukotriene D4 metabolic process | 11.90098464 | 9.55E-10 | 3.88E-06 | 3.76E-06 | *GGT1/GGTA1/GGTLC2/DPEP1/GGT7/GGT2P/GGT3P/GGTLC1* | 8 |
| BP | GO:1901750 | leukotriene D4 biosynthetic process | 11.43680272 | 6.89E-09 | 1.40E-05 | 1.36E-05 | *GGT1/GGTA1/GGTLC2/GGT7/GGT2P/GGT3P/GGTLC1* | 7 |
| BP | GO:0006751 | glutathione catabolic process | 10.84737432 | 1.84E-08 | 2.49E-05 | 2.42E-05 | *GGT1/GGTLC2/DPEP1/GGT7/GGT2P/GGT3P/GGTLC1* | 7 |
| BP | GO:0009060 | aerobic respiration | 6.984830768 | 3.38E-08 | 3.33E-05 | 3.24E-05 | *NDUFA12/SURF1/NDUFA4/COX7A2/ABCD1/NDUFB1/UQCC2/SUCLG2/ATP5F1C/NDUFAB1/NDUFS7/ANTKMT/NDUFS6/RHOA/NDUFA13/GHITM/ATP5F1E/ARL2/BLOC1S1/NDUFB7/PARK7/ADSL/NDUFS3/IDH3B/NDUFC2-KCTD14* | 25 |
| BP | GO:0033108 | mitochondrial respiratory chain complex assembly | 7.30215485 | 7.61E-08 | 5.15E-05 | 5.00E-05 | *NDUFA12/COX17/SURF1/NDUFB1/UQCC2/NDUFAB1/NDUFS7/NDUFA13/NDUFAF3/TMEM223/COA8/PET100/NDUFB7/SMIM20/DMAC2/NDUFS3/NDUFAF2* | 17 |
| BP | GO:0032543 | mitochondrial translation | 6.912025632 | 1.37E-07 | 7.94E-05 | 7.71E-05 | *MRPL58/MRPL21/UQCC2/MRPL53/MRPL23/MRPL54/DAP3/MRPL41/MRPS15/MRPS34/MRPL34/MRPS25/GADD45GIP1/MRPL51/MRPS7/MRPL24/MRPL16/PTCD1/MRPS11* | 19 |
| BP | GO:0019370 | leukotriene biosynthetic process | 8.623949059 | 2.96E-07 | 0.000150443 | 0.000146074 | *GGT1/GGTA1/MGST2/GGTLC2/GGT7/GGT2P/GGT3P/GGTLC1* | 8 |
| BP | GO:0046034 | ATP metabolic process | 6.006680756 | 8.90E-07 | 0.000361452 | 0.000350956 | *PRKAA2/NDUFA12/GAPDH/AK1/GALK1/OGT/PFKP/NDUFB1/GPI/ATP5F1C/NDUFAB1/NDUFS7/SLC25A25/ANTKMT/NDUFS6/OLA1/FIS1/NDUFA13/ATP5F1E/ARL2/NDUFB7/AK2/BAD/NDUFS3* | 24 |
| BP | GO:0006119 | oxidative phosphorylation | 6.198100047 | 1.06E-06 | 0.000392839 | 0.000381432 | *NDUFA12/NDUFA4/COX7A2/ABCD1/NDUFB1/UQCC2/ATP5F1C/NDUFAB1/NDUFS7/ANTKMT/NDUFS6/RHOA/NDUFA13/GHITM/ATP5F1E/NDUFB7/PARK7/NDUFS3/NDUFC2-KCTD14* | 19 |
| BP | GO:0140053 | mitochondrial gene expression | 6.077818425 | 1.27E-06 | 0.00042974 | 0.000417262 | *TWNK/MRPL58/MRPL21/UQCC2/MRPL53/MRPL23/MRPL54/DAP3/MRPL41/MRPS15/MRPS34/MRPL34/MRPS25/GADD45GIP1/MRPL51/MRPS7/MRPL24/MRPL16/PTCD1/MRPS11* | 20 |
| BP | GO:0009205 | purine ribonucleoside triphosphate metabolic process | 5.762944876 | 1.70E-06 | 0.000523796 | 0.000508586 | *PRKAA2/NDUFA12/GAPDH/AK1/GALK1/OGT/PFKP/NDUFB1/GPI/ATP5F1C/NDUFAB1/NDUFS7/SLC25A25/ANTKMT/NDUFS6/NME2/OLA1/FIS1/NDUFA13/ATP5F1E/ARL2/NDUFB7/AK2/BAD/NDUFS3* | 25 |
| BP | GO:0006691 | leukotriene metabolic process | 7.28803602 | 1.81E-06 | 0.000523796 | 0.000508586 | *GGT1/GGTA1/MGST2/GGTLC2/DPEP1/GGT7/GGT2P/GGT3P/GGTLC1* | 9 |
| BP | GO:0044273 | sulfur compound catabolic process | 6.536315204 | 5.12E-06 | 0.001223699 | 0.001188165 | *GGT1/IDUA/GGTLC2/DPEP1/GGT7/ABCD1/GGT2P/SUCLG2/GGT3P/GGTLC1* | 10 |
| BP | GO:0043161 | proteasome-mediated ubiquitin-dependent protein catabolic process | 5.11634335 | 7.63E-06 | 0.001650467 | 0.001602541 | *RNF180/ARRB1/UBXN2B/STYX/OGT/RAD23A/PSMB7/DCAF13/PSMB4/PSMA2/ANAPC15/PSMD13/FBXO38/RBX1/PSMD2/PSMD8/BFAR/PSMC3/PSMA7/PSMB2/ANAPC11/PSMB1/PARK7/SHARPIN/UBE2K/HECTD3/SMARCC1/DMAC2/BAG6/PSMC2/FBXW5/PSMC5* | 32 |
| BP | GO:0009150 | purine ribonucleotide metabolic process | 5.046756105 | 8.13E-06 | 0.001650467 | 0.001602541 | *PRKAA2/NDUFA12/GAPDH/LRGUK/AK1/TPST1/GALK1/OGT/COASY/PFKP/ABCD1/NDUFB1/GPI/HINT1/GPAT4/SUCLG2/ATP5F1C/NDUFAB1/NDUFS7/SLC25A25/ANTKMT/NDUFS6/MCCC2/NME2/OLA1/ACOT9/FIS1/NDUFA13/ATP5F1E/ARL2/NDUFB7/ADSL/AK2/BAD/NDUFS3* | 35 |
| BP | GO:0098974 | postsynaptic actin cytoskeleton organization | 7.130172361 | 1.61E-05 | 0.002880171 | 0.002796536 | *POTEF/ACTB/ACTG1/POTEJ/POTEI/POTEE* | 6 |
| BP | GO:0019693 | ribose phosphate metabolic process | 4.821042158 | 1.63E-05 | 0.002880171 | 0.002796536 | *PRKAA2/TALDO1/NDUFA12/GAPDH/LRGUK/AK1/TPST1/GALK1/OGT/COASY/PFKP/ABCD1/NDUFB1/GPI/HINT1/GPAT4/SUCLG2/ATP5F1C/NDUFAB1/NDUFS7/SLC25A25/ANTKMT/NDUFS6/MCCC2/NME2/OLA1/ACOT9/FIS1/NDUFA13/ATP5F1E/ARL2/NDUFB7/ADSL/AK2/BAD/NDUFS3* | 36 |
| BP | GO:0042776 | proton motive force-driven mitochondrial ATP synthesis | 5.75754478 | 1.94E-05 | 0.003286445 | 0.003191013 | *NDUFA12/NDUFB1/ATP5F1C/NDUFAB1/NDUFS7/ANTKMT/NDUFS6/NDUFA13/ATP5F1E/NDUFB7/NDUFS3* | 11 |
| BP | GO:0031647 | regulation of protein stability | 4.42745318 | 8.95E-05 | 0.011726018 | 0.011385516 | *EPHA4/MT3/C10orf90/GLMP/GAPDH/CTSA/SERF1A/SERF1B/RAD23A/DAZAP2/PRKDC/UXT/PFN1/SWSAP1/CD81/COA8/HDAC3/PIH1D2/PARK7/BAG6/NCLN/PPIB/SERF2/APTX/HYPK/DDOST* | 26 |
| BP | GO:0042219 | cellular modified amino acid catabolic process | 5.655294181 | 9.86E-05 | 0.012505041 | 0.012141919 | *GGT1/GGTLC2/DPEP1/GGT7/GGT2P/GGT3P/GGTLC1* | 7 |
| BP | GO:0006486 | protein glycosylation | 4.417736684 | 0.000135642 | 0.016197286 | 0.015726947 | *GALNT10/POMT1/ALG10B/TMTC2/ABCA2/B3GALT1/OGT/ST6GALNAC6/RFT1/ALG11/ST3GAL3/KRTCAP2/GOLGA2/ALG9/POFUT1/PLOD3/ALG14/TUSC3/STT3A/DDOST* | 20 |
| BP | GO:0043413 | macromolecule glycosylation | 4.417736684 | 0.000135642 | 0.016197286 | 0.015726947 | *GALNT10/POMT1/ALG10B/TMTC2/ABCA2/B3GALT1/OGT/ST6GALNAC6/RFT1/ALG11/ST3GAL3/KRTCAP2/GOLGA2/ALG9/POFUT1/PLOD3/ALG14/TUSC3/STT3A/DDOST* | 20 |
| BP | GO:0006749 | glutathione metabolic process | 5.050735713 | 0.00015092 | 0.01750672 | 0.016998358 | *GGT1/MGST2/GGTLC2/DPEP1/GGT7/GGT2P/GGT3P/PARK7/GGTLC1* | 9 |
| BP | GO:0042398 | cellular modified amino acid biosynthetic process | 5.127420346 | 0.000175235 | 0.019762619 | 0.01918875 | *GGT1/SLC27A1/MGST2/GGT7/ALDH9A1/GGT3P/PLOD3/PARK7* | 8 |
| BP | GO:0009100 | glycoprotein metabolic process | 4.046637492 | 0.00024317 | 0.024079798 | 0.023380566 | *CYTL1/MANBA/PORCN/IDUA/CST3/GALNT10/POMT1/ALG10B/TMTC2/ABCA2/B3GALT1/OGT/ADAMTS13/ST6GALNAC6/RFT1/ALG11/ST3GAL3/KRTCAP2/GOLGA2/ALG9/POFUT1/PLOD3/ALG14/TUSC3/PARK7/GANAB/STT3A/DDOST* | 28 |
| BP | GO:0070085 | glycosylation | 4.072718345 | 0.000337964 | 0.032669882 | 0.03172121 | *GALNT10/POMT1/ALG10B/TMTC2/ABCA2/B3GALT1/OGT/ST6GALNAC6/RFT1/ALG11/ST3GAL3/KRTCAP2/GOLGA2/ALG9/POFUT1/PLOD3/ALG14/TUSC3/STT3A/DDOST* | 20 |
| BP | GO:1902414 | protein localization to cell junction | 4.310492678 | 0.000401874 | 0.037305037 | 0.03622177 | *GRIP2/GRIP1/C1QL3/HSPB1/IQSEC2/ZDHHC12/OGT/ACTB/HEG1/ACTG1/LAMTOR2/PAK2* | 12 |
| BP | GO:0043467 | regulation of generation of precursor metabolites and energy | 4.194373735 | 0.000410199 | 0.037305037 | 0.03622177 | *PRKAA2/COX17/NDUFA4/PPP1R3G/COX7A2/OGT/ABCD1/UQCC2/RHOA/GHITM/ARL2/COA8/PARK7/PPP1CA* | 14 |
| BP | GO:0016226 | iron-sulfur cluster assembly | 5.035893558 | 0.000431856 | 0.037305037 | 0.03622177 | *FDX2/NDUFAB1/GLRX3/NFU1/IBA57/NUBP2* | 6 |
| BP | GO:0016197 | endosomal transport | 3.826340092 | 0.000575475 | 0.044931283 | 0.043626563 | *GRIP2/GRIP1/GOLT1A/VPS29/AP5M1/STX8/SCARB2/RAB9B/WASHC3/CHMP2A/UBXN6/VTI1B/VPS25/BLOC1S1/HGS/ARL8B/SNX32/MVB12A/EIPR1/CHMP4A/SNX17/RAB11B* | 22 |
| BP | GO:0045048 | protein insertion into ER membrane | 4.802481712 | 0.000630684 | 0.046555932 | 0.045204035 | *EMC6/GET3/TMEM147/BAG6/NCLN/WDR83OS* | 6 |
| CC | GO:0005743 | mitochondrial inner membrane | 8.006116375 | 2.68E-11 | 1.32E-08 | 1.18E-08 | *NDUFA12/SLC27A1/SURF1/MTLN/NDUFA4/SLC25A27/TMEM14C/COX7A2/TWNK/MRPL58/SMDT1/NDUFB1/MRPL21/UQCC2/MRPL53/MRPL23/MRPL54/DAP3/ATP5F1C/NDUFAB1/MRPL41/NDUFS7/SLC25A25/NDUFS6/TIMMDC1/ENDOG/MRPS15/MRPS34/NDUFA13/NDUFAF3/TMEM223/GHITM/ATP5F1E/MRPL34/COA8/MRPS25/PET100/NDUFB7/SMIM20/SLC25A38/GADD45GIP1/MRPL51/MRPS7/DMAC2/NDUFS3/NDUFAF2/MRPL24/NDUFC2-KCTD14/MRPL16/MRPS11* | 50 |
| CC | GO:0098798 | mitochondrial protein-containing complex | 8.247432711 | 5.11E-11 | 1.32E-08 | 1.18E-08 | *NDUFA12/NDUFA4/COX7A2/MRPL58/SMDT1/NDUFB1/MRPL21/SUCLG2/MRPL53/MRPL23/MRPL54/DAP3/ATP5F1C/NDUFAB1/MRPL41/NDUFS7/NDUFS6/MCCC2/MRPS15/MRPS34/NDUFA13/ATP5F1E/MRPL34/MRPS25/NDUFB7/TOMM20L/GADD45GIP1/TOMM6/MRPL51/MRPS7/DMAC2/NDUFS3/MRPL24/IDH3B/NDUFC2-KCTD14/MRPL16/MRPS11* | 37 |
| CC | GO:1905369 | endopeptidase complex | 8.799480748 | 8.55E-10 | 1.47E-07 | 1.31E-07 | *CASP9/HSPB1/RAD23A/PSMB7/CAPNS1/PSMB4/PSMA2/PSMD13/PSMD2/PSMD8/PSMC3/PSMA7/PSMB2/PSMB1/CAPN1/PSMC2/PSMD9/PSMC5* | 18 |
| CC | GO:0000313 | organellar ribosome | 8.012412257 | 1.09E-08 | 9.41E-07 | 8.40E-07 | *MRPL58/MRPL21/MRPL53/MRPL23/MRPL54/DAP3/MRPL41/MRPS15/MRPS34/MRPL34/MRPS25/GADD45GIP1/MRPL51/MRPS7/MRPL24/MRPL16/MRPS11* | 17 |
| CC | GO:0005761 | mitochondrial ribosome | 8.012412257 | 1.09E-08 | 9.41E-07 | 8.40E-07 | *MRPL58/MRPL21/MRPL53/MRPL23/MRPL54/DAP3/MRPL41/MRPS15/MRPS34/MRPL34/MRPS25/GADD45GIP1/MRPL51/MRPS7/MRPL24/MRPL16/MRPS11* | 17 |
| CC | GO:0045271 | respiratory chain complex I | 7.018416022 | 1.21E-06 | 6.97E-05 | 6.22E-05 | *NDUFA12/NDUFA4/NDUFB1/NDUFAB1/NDUFS7/NDUFS6/NDUFA13/NDUFB7/DMAC2/NDUFS3/NDUFC2-KCTD14* | 11 |
| CC | GO:1904813 | ficolin-1-rich granule lumen | 5.703302053 | 7.12E-06 | 0.000262767 | 0.000234332 | *CST3/DYNLT1/GPI/PSMB7/PSMA2/ACTR1B/PSMD13/PSMD2/APEH/NME2/PSMC3/PSMB1/CAPN1/XRCC6/PSMC2/PDAP1* | 16 |
| CC | GO:1990204 | oxidoreductase complex | 4.956402559 | 5.69E-05 | 0.001547908 | 0.001380401 | *NDUFA12/CYB5R3/NDUFA4/HSD17B8/NDUFB1/NDUFAB1/NDUFS7/NDUFS6/NDUFA13/NDUFB7/DMAC2/NDUFS3/CYB5B/IDH3B/NDUFC2-KCTD14* | 15 |
| CC | GO:0035267 | NuA4 histone acetyltransferase complex | 4.689512102 | 0.000758273 | 0.012250843 | 0.010925113 | *POTEF/ACTB/ACTG1/POTEJ/POTEI/POTEE* | 6 |
| CC | GO:0008250 | oligosaccharyltransferase complex | 5.098297349 | 0.001135187 | 0.01630254 | 0.014538354 | *KRTCAP2/TUSC3/STT3A/DDOST* | 4 |
| CC | GO:0072379 | ER membrane insertion complex | 5.098297349 | 0.001135187 | 0.01630254 | 0.014538354 | *TMEM147/BAG6/NCLN/WDR83OS* | 4 |
| CC | GO:0036452 | ESCRT complex | 4.022096338 | 0.003091898 | 0.042066093 | 0.037513893 | *CHMP2A/VPS25/HGS/MVB12A/CHMP4A* | 5 |
| MF | GO:0098973 | structural constituent of postsynaptic actin cytoskeleton | 9.406465447 | 4.79E-07 | 0.000371383 | 0.000361673 | *POTEF/ACTB/ACTG1/POTEJ/POTEI/POTEE* | 6 |
| MF | GO:0008242 | omega peptidase activity | 7.427529145 | 9.73E-06 | 0.003771259 | 0.003672656 | *GGT1/GGTLC2/GGT7/APEH/GGT3P/GGTLC1* | 6 |
| MF | GO:0070003 | threonine-type peptidase activity | 6.419781657 | 4.64E-05 | 0.011996378 | 0.01168272 | *GGT1/GGTLC2/GGT7/PSMB7/GGT3P/GGTLC1* | 6 |
| MF | GO:0003735 | structural constituent of ribosome | 4.183224348 | 0.000314951 | 0.048817365 | 0.047540986 | *LINC01004/MRPL21/MRPL23/MRPL54/DAP3/MRPL41/RPL37A/MRPS15/MRPS34/MRPL34/MRPS25/RPS19/MRPL51/MRPS7/MRPL24/MRPL16/MRPS11* | 17 |

**Table S13 Molecular docking results of DOC2A with neuroactive compounds**

| Category | Drug Name | DrugBank ID | PubChem CID | Binding Energy, kcal/mol | Binding Residues |
| --- | --- | --- | --- | --- | --- |
| Prioritized candidates with transcriptomic relevance | Valproic acid | DB00313 | 3121 | -4.0 | K130, K142, K144, R176 |
|  | MDMA | DB01454 | 1615 | -5.1 | K130, H132, K142, R176 |
|  | Schisandrin B | - | 108130 | -5.7 | K130, H132, K142, K144 |
|  | Resveratrol | DB02709 | 445154 | -5.7 | K130, H132, K142, K144 |
| Prioritized candidates with  clinical references | Lamotrigine | DB00555 | 3878 | -4.7 | K130, A178, F190 |
|  | Carbamazepine | DB00564 | 2554 | -6.4 | K130, K142 |
| Pharmacological Controls | Levetiracetam | DB01202 | 5284583 | -3.7 | - |
|  | Topiramate | DB00273 | 5284627 | -4.5 | Y128, K142, K144 |
